# Supplementary material for: Overtemperature-protection intelligent molecular chiroptical photoswitches
Source: Nat Commun. 2021 May 10;12:2600. doi: 10.1038/s41467-021-22880-z (PMC8110520; doi:10.1038/s41467-021-22880-z)
Supplement: Supplementary file 1 — Supplementary Information [file 41467_2021_22880_MOESM1_ESM.pdf]

Supporting Information

Overtemperature-Protection Intelligent Molecular Chiroptical Photoswitches

Jiabin Yao,[1] Wanhua Wu, *,[1] Chao Xiao,[1] Dan Su,[1] Zhihui Zhong,[1] Tadashi Mori,[2] Cheng Yang*,[1]

[1] *Key Laboratory of Green Chemistry & Technology of Ministry of Education, College of Chemistry, State Key Laboratory of Biotherapy, and Healthy Food Evaluation Research Center, Sichuan University, Chengdu 610064, China*

[2] *Department of Applied Chemistry, Osaka University, Suita 565-0871, Japan*

Contents

1. Materials and Instruments 2

2. Synthesis and Characterization 3

3. NMR and HR-Mass Spectra 7

4. Isoabsorptive Point of MUJs 22

5. Chiral HPLC Analysis 24

6. CD Spectra 29

7. Solvent Effects on MUJs 33

8. Light-Driven Chirality Switching 35

9. Chirality Switching in Coating Film 46

10. Photoresponsive ^{1}H NMR Spectra of MUJs 48

11. Molecular Model Studies of **MUJ2** 50

12. VT CD Spectra of *trans*-(*in*-R_p/*out*-S_p)-**MUJ1** 51

13. VT CD Spectra of *trans*-(*in*-R_p/*out*-S_p)-**MUJ2** 58

14. VT CD Spectra of *trans*-(*in*-R_p/*out*-S_p)-**MUJ3** 65

15 Thermodynamic Parameters of the out-to-in Equilibrium 72

16. Temperature-Regulated Light-Driven Chirality Switching 76

17. Calculated CD Spectra of **MUJ1** 98

References 98

1. Materials and Instruments

p-Nitrophenol, triethylene glycol, tetraethylene glycol, 1-bromobutane, 1,4-diazabicyclo[2.2.2]octane and all solvents were used as supplied without further purification. Pillar[n]arenes **DEP5**[S1] and **DEP6**[S2] were synthesized according to literature procedures. ^{1}H and ^{13}C NMR spectra at 400 and 100 MHz, respectively, were measured at ambient temperature on a Bruker AVANCE III HD 400 MHz spectrometer, and all chemical shifts are reported in ppm relative to TMS. High-resolution mass spectra were obtained with a Waters-Q-TOF-Premier (ESI) and reported in units of mass to charge (*m/z*). UV-vis spectra were recorded on a JASCO V650 spectrometer. CD spectra were measured on a JASCO J-1500 spectrometer using a quartz cuvette with a 1 cm path length equipped with Peltier thermostatic cell holders.

2. Synthesis and Characterization

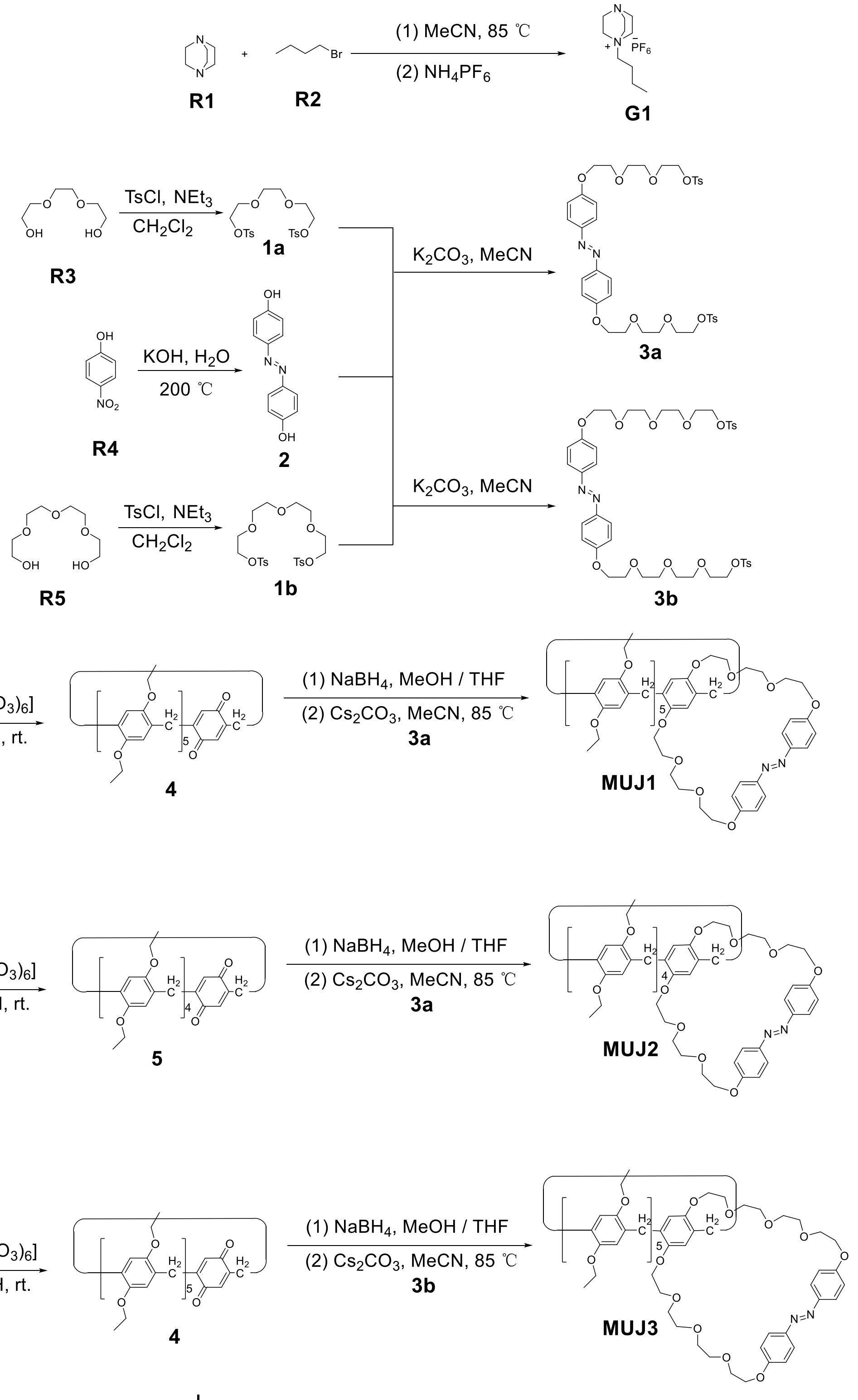

Supplementary Fig. 1 | Synthesis of **MUJ1**, **MUJ2** and **MUJ3**.

1a: To a solution of triethylene glycol (50 mmol) in dichloromethane (120 mL), 30 ml NEt_3 and TsCl (100 mmol) were added in batches. The suspension mixture was stirred at room temperature for 24 h. After removal of the solvent and NEt_3 under reduced pressure, the resulting solid was recrystallized in EtOH, and a white solid (13 g, 57%) was afforded after drying in a vacuum oven. ^{1}H NMR (400 MHz, $CDCl_3$): δ 7.79 (d, J = 8.3 Hz, 4H), 7.34 (d, J = 8.0 Hz, 4H), 4.16 – 4.12 (t, J = 4.1 Hz, 4H), 3.66 (t, J = 3.7 Hz, 4H), 3.53 (s, 4H), 2.45 (s, 6H). ^{13}C NMR (100 MHz, $CDCl_3$) δ 144.91, 132.92, 129.88, 127.97, 70.69, 69.24, 68.74, 21.67. HRMS (m/z): $[C_{20}H_{26}O_8S_2Na]^+$ calcd for $C_{20}H_{26}O_8S_2$, m/z = 481.0961; found, m/z = 481.0960.

2: Potassium hydroxide (30 g) was dissolved in 5.4 mL H_2O, and *p*-nitrophenol (5.2 g, 37.4 mmol) was added into the mixture and heated to 200 °C under vigorous stirring for 2 h. The mixture was cooled to room temperature, and the reaction mixture was acidized in diluted hydrochloric acid aqueous solution. After suction filtration, the resulting solid was washed with deionized water and dried in a vacuum oven. Compound **2** was afforded as a tawny solid (2.1 g, 52%). ^{1}H NMR (400 MHz, DMSO-d6): δ 10.14 (s, 2H), 7.76 – 7.67 (d, J = 7.7 Hz, 4H), 6.91 (d, J = 8.8 Hz, 4H). ^{13}C NMR (100 MHz, acetone-d6): δ 159.88, 146.30, 124.29, 115.69. HRMS (m/z): $[C_{12}H_9N_2O_2]^-$ calcd. for $C_{12}H_{10}N_2O_2$, 213.0670; found, 213.0656.

3a: A solution of **2** (700 mg, 3.3 mmol), compound **1a** (3.7 g, 8.0 mmol), and K_2CO_3 (3.4 g, 25 mmol) in 130 mL acetonitrile was heated to 85 °C for 10 h under a nitrogen atmosphere. Thereafter, the solvent was removed by rotary evaporation, the reaction mixture was dissolved in 250 mL CH_2Cl_2, and the organic phase was washed with 1 M HCl and water (250 mL × 3). The separated organic phase was dried over anhydrous sodium sulfate, and then, the solvent was removed after filtration. The mixture was purified by silica gel column chromatography using the eluent of dichloromethane/PE/ethyl acetate = 1 : 1 : 0.5, and compound **3a** was afforded as a yellow solid (800 mg, 34%). ^{1}H NMR (400 MHz, $CDCl_3$): δ 7.86 (d, J = 7.9 Hz, 4H), 7.79 (d, J = 7.8 Hz, 4H), 7.32 (d, J = 7.3 Hz, 4H), 7.00 (d, J = 7.0 Hz, 4H), 4.17 (m, 8H), 3.88 – 3.83 (t, J = 3.9 Hz, 4H), 3.73 – 3.65 (m, 8H), 3.65 – 3.60 (m, 4H), 2.42 (s, 6H). ^{13}C NMR (100 MHz, $CDCl_3$): δ 160.80, 147.15, 144.83, 133.02, 129.84, 127.98, 124.34, 114.82, 70.83, 69.72, 69.26, 68.77, 67.70, 21.64. HRMS (m/z): $[C_{38}H_{46}N_2O_{12}S_2Na]^+$ calcd. for $C_{38}H_{46}N_2O_{12}S_2$, 809.2384; found, 809.2395.

3b: A solution of **2** (1.05 g, 5 mmol), compound **1b** (10.8 g, 20 mmol), and Cs_2CO_3 (2.4 g, 7.5 mmol) in 120 mL acetonitrile was heated to 85 °C for 18 h under a nitrogen atmosphere. Thereafter, the solvent was removed by rotary evaporation, the reaction mixture was dissolved in 250 mL CH_2Cl_2, and the organic phase was washed with 1 M HCl and water (250 mL × 3). The separated organic phase was dried over anhydrous sodium sulfate, and then, the solvent was removed after filtration. The mixture was purified by silica gel column chromatography using the eluent of dichloromethane/PE/ethyl acetate = 1 : 1 : 1, and compound **3b** was afforded as a yellow solid (1.7 g, 40%). ^{1}H NMR (400 MHz, $CDCl_3$): δ 7.90 (d, J = 8.9 Hz, 4H), 7.79 (d, J = 8.3 Hz, 4H), 7.33 (d, J = 8.0 Hz, 4H), 7.01 (d, J = 9.0 Hz, 4H), 4.23 – 4.18 (t, J = 4.2 Hz, 4H), 4.15 (t, J = 4.15 Hz, 4H), 3.91 – 3.86 (t, J = 3.9 Hz, 4H), 3.75 – 3.71 (m, 4H), 3.70 – 3.65 (m, 8H), 3.60 (s, 8H), 2.43 (s, 6H). ^{13}C NMR (100 MHz, $CDCl_3$): δ 160.83, 146.96, 144.80, 132.91, 129.81, 127.94, 124.34, 114.80, 70.82, 70.72, 70.64, 70.53, 69.62, 69.25, 68.65, 67.68, 21.63. HRESI (m/z): $[C_{42}H_{54}N_2O_{14}S_2Na]^+$ calcd. for $C_{42}H_{54}N_2O_{14}S_2Na$, 897.2909; found, 897.2893.

G1: A solution of 1,4-diazabicyclo[2.2.2]octane (2.1 g, 18.7 mmol) and 1-bromobutane (0.53 mL, 4.9 mmol) in 50 mL acetonitrile was heated to 85 °C for 48 h under a nitrogen atmosphere. Thereafter, the solvent was removed by rotary evaporation, and the reaction mixture was dissolved in a 150 mL NH_4PF_6 saturated solution. The mixture was purified by a C18 chromatographic column using the eluent of H_2O and 50% EtOH aqueous solution, and after freeze-drying, compound **G1** was afforded as a white solid (1.5 g, 4.8 mmol, 98%). ^{1}H NMR (400 MHz, CD_3OD): δ 3.37 – 3.32 (t, J = 3.34 Hz, 6H), 3.20 (m, 8H), 1.78–1.67 (m, 2H), 1.40 (m, 2H), 1.01 (t, J = 7.4 Hz, 3H). ^{13}C NMR (100 MHz, CD_3OD): δ 64.21, 52.00, 44.63, 23.32, 19.37, 12.48. HRMS (m/z): $[C_{20}H_{42}N_4PF_6]^+$ calcd. for $C_{10}H_{21}N_2PF_6$, 483.3046; found, m/z = 483.3031.

4: To a solution of **DEP6** (1.5 g, 1.5 mmol) in dichloromethane (25 mL), 2 mL MeOH solution of $(NH_4)_2[Ce(NO_3)_6]$ (411 mg, 0.75 mmol) was added dropwise. After stirring overnight at room temperature, the solvent was removed, and the reaction mixture was dissolved in 250 mL CH_2Cl_2. Then, the organic phase was washed with deionized water, and the separated organic

phase was dried over anhydrous sodium sulfate. The mixture was purified by silica gel column chromatography after filtration using the eluent of dichloromethane/PE/EA = 50 : 100 : 3, affording compound **4** as a crimson solid (190 mg, 0.19 mmol, 13%) and **DEP6** (500 mg, 0.5 mmol, 33%). ^{1}H NMR (400 MHz, $CDCl_3$): δ 6.75 (s, 2H), 6.70 (s, 2H), 6.69 (s, 2H), 6.67 (s, 2H), 6.61 (s, 2H), 6.43 (s, 2H), 3.95 – 3.72 (m, 28H), 3.57 (s, 4H), 1.29 (m, 30H). ^{13}C NMR (100 MHz, $CDCl_3$): δ 188.33, 150.54, 150.41, 150.38, 146.54, 133.62, 129.26, 128.16, 127.89, 127.17, 122.57, 115.46, 115.21, 115.18, 115.07, 114.45, 64.19, 64.10, 64.06, 63.93, 63.52, 31.06, 30.61, 30.36, 15.22, 15.19, 15.13, 14.96. HRMS (m/z): ([$C_{62}H_{74}O_{12}Na$]$^{+}$) calcd. for $C_{62}H_{74}O_{12}$, 1033.5072; found, 1033.5142.

5: **DEP5** (2.87 g, 3.2 mmol) was dissolved in 150 mL CH_2Cl_2, and 15 mL $(NH_4)_2[Ce(NO_3)_6]$ (1.65 g, 3.2 mmol) solution in MeOH was added into the reaction mixture. After stirring overnight at room temperature, the solvent was removed, and the reaction mixture was dissolved in 250 mL CH_2Cl_2. Then, the organic phase was washed with deionized water, and the separated organic phase was dried over anhydrous sodium sulfate. The mixture was purified by silica gel column chromatography after filtration using the eluent of dichloromethane/petroleum ether/ethyl acetate = 40 : 160 : 3, and compound **5** was afforded as a crimson solid (1.0 g, 12 mmol, 37%). ^{1}H NMR (400 MHz, $CDCl_3$): δ 6.82 (s, 2H), 6.80 (s, 2H), 6.78 (s, 2H), 6.66 (s, 2H), 6.65 (s, 2H), 3.78-3.95 (m, 22H), 3.57 (s, 4H), 1.33-1.43 (m, 18H), 1.23-1.26 (t, J = 6.8 Hz, 6H). ^{13}C NMR (100 MHz, $CDCl_3$): δ 188.62, 150.16, 149.86, 149.81, 146.58, 133.35, 129.62, 128.63, 127.94, 123.35, 115.07, 114.97, 114.74, 114.47, 64.05, 63.75, 63.67, 63.38, 31.56, 29.53, 29.26, 28.21, 22.61, 15.28, 15.22, 15.13, 14.95, 14.13, 0.03. HRMS (m/z): ([$C_{51}H_{60}O_{10}Na$]$^{+}$) calcd. for $C_{51}H_{60}O_{10}$, 855.4079; found, 855.4080.

MUJ1: To a solution of compound **4** (200 mg, 0.2 mmol) in THF (20 mL) and MeOH (5 mL) was added $NaBH_4$ (300 mg, 7.9 mmol). The mixture was stirred at room temperature for 20 min. dichloromethane (150 mL) was added to the reaction mixture, which was then poured into diluted HCl aqueous solution. The separated organic layer was dried over anhydrous sodium sulfate and evaporated after filtration, affording white solid dihydroxylated pillar[6]arene (197 mg, 97%). To a solution of dihydroxylated pillar[6]arene (197 mg, 0.2 mmol) and compound **3a** (175 mg, 0.2 mmol) in acetonitrile (125 mL) was added anhydrous caesium carbonate (350 mg, 1.1 mmol) under a nitrogen atmosphere. The mixture was stirred at 85 °C for 48 h. After removal of the solvent under reduced pressure, the resulting solid was acidized and poured into CH_2Cl_2. The separated organic phase was washed with water and dried over anhydrous sodium sulfate, and then, the solvent was removed after filtration. The mixture was purified by silica gel column chromatography (petroleum ether/dichloromethane/ethyl acetate = 1 : 1 : 0.3) and dried in a vacuum oven to afford tawny solid **MUJ1** (50 mg, 0.035 mmol, 17%). ^{1}H NMR (400 MHz, $CDCl_3$): δ 7.40 (d, J = 7.8 Hz, 4H), 6.79 (s, 2H), 6.74 (s, 2H), 6.65 (m, 8H), 6.59 (s, 2H), 6.38 (s, 2H), 4.13 (m, 2H), 3.90 – 3.46 (m, 54H), 1.32 – 1.20 (m, 30H). ^{13}C NMR (100 MHz, $CDCl_3$): δ 159.39, 149.53, 149.48, 149.36, 149.28, 149.17, 149.10, 145.80, 126.53, 126.42, 126.20, 126.09, 122.93, 114.42, 114.33, 114.29, 114.13, 114.02, 113.77, 113.55, 70.14, 69.83, 69.36, 69.08, 66.56, 66.28, 62.82, 62.64, 62.58, 30.77, 30.22, 30.03, 28.68, 14.29, 14.27, 14.17. HRMS (m/z): [$C_{86}H_{106}O_{18}N_2H$]$^{+}$ calcd. for $C_{86}H_{106}O_{18}N_2$, 1455.7513; found, 1455.7511.

MUJ2: To a solution of compound **5** (300 mg, 0.36 mmol) in THF (20 mL) and MeOH (5 mL) was added $NaBH_4$ (300 mg, 7.9 mmol). The mixture was stirred at room temperature for 20 min. dichloromethane (150 mL) was added to the reaction mixture, which was then poured into diluted HCl aqueous solution. The separated organic layer was dried over anhydrous sodium sulfate and evaporated after filtration, affording white solid dihydroxylated pillar[5]arene (295 mg, 98%). To a solution of dihydroxylated pillar[5]arene (295 mg, 0.36 mmol) and compound **3a** (315 mg, 0.36 mmol) in acetonitrile (125 mL) was added anhydrous caesium carbonate (1.05 mg, 3.3 mmol) under a nitrogen atmosphere. The mixture was stirred at 85 °C for 48 h. After removal of the solvent under reduced pressure, the resulting solid was acidized and poured into CH_2Cl_2. The separated organic phase was washed with water and dried over anhydrous sodium sulfate, and then, the solvent was removed after filtration. The mixture was purified by silica gel column chromatography (petroleum ether/dichloromethane/ethyl acetate = 1 : 1 : 0.25) and dried in a vacuum oven to afford tawny solid **MUJ2** (32 mg, 0.025 mmol, 7%). ^{1}H NMR (400 MHz, $CDCl_3$): δ 7.72 (d, J = 8.7 Hz, 4H), 7.05 (d, J = 8.9 Hz, 4H), 6.70 (s, 2H), 6.69 (s, 2H), 6.68 (s, 2H), 6.67 (s, 2H), 6.53 (s, 2H), 4.33 (m, 4H), 3.87 – 3.45 (m, 46H), 1.24 (m, 24H). ^{13}C NMR (100 MHz, $CDCl_3$): δ 160.88,

149.90, 149.88, 149.82, 149.77, 149.74, 147.15, 128.62, 128.56, 128.40, 128.32, 124.22, 115.66, 115.24, 115.15, 115.10, 115.06, 115.01, 71.39, 70.97, 70.46, 70.37, 68.10, 67.75, 63.81, 63.73, 29.82, 29.72, 29.63, 15.06. HRMS (m/z): $[C_{75}H_{92}O_{16}N_2HK]^{2+}$ calcd. for $C_{75}H_{92}O_{16}N_2$, m/z = 658.3076; found, m/z = 658.2981.

MUJ3: To a solution of compound **4** (200 mg, 0.2 mmol) in THF (20 mL) and MeOH (5 mL) was added $NaBH_4$ (300 mg, 7.9 mmol). The mixture was stirred at room temperature for 20 min. dichloromethane (150 mL) was added to the reaction mixture, which was then poured into diluted HCl aqueous solution. The separated organic layer was dried over anhydrous sodium sulfate and evaporated after filtration, affording white solid dihydroxylated pillar[6]arene (197 mg, 97%). To a solution of dihydroxylated pillar[6]arene (197 mg, 0.2 mmol) and compound **3b** (175 mg, 0.2 mmol) in acetonitrile (125 mL) was added anhydrous caesium carbonate (350 mg, 1.1 mmol) under a nitrogen atmosphere. The mixture was stirred at 85 °C for 30 h. After removal of the solvent under reduced pressure, the resulting solid was acidized and poured into CH_2Cl_2. The separated organic phase was washed with water and dried over anhydrous sodium sulfate, and then, the solvent was removed after filtration. The mixture was purified by silica gel column chromatography (petroleum ether/dichloromethane/ethyl acetate = 1.5 : 1 : 1) and dried in a vacuum oven to afford tawny solid **MUJ3** (60 mg, 0.04 mmol, 20%). ^1H NMR (400 MHz, $CDCl_3$): δ 7.58 (d, J = 8.3 Hz, 4H), 6.75 (s, 2H), 6.72 (d, J = 8.3 Hz, 2H), 6.71 (s, 2H), 6.68 (s, 2H), 6.66 (s, 2H), 6.63 (s, 2H), 6.51 (s, 2H), 4.15 – 4.08 (m, 2H), 3.98 (m, 2H), 3.84 – 3.51 (m, 60H), 1.31 – 1.22 (m, 30H). ^{13}C NMR (100 MHz, $CDCl_3$): δ 159.49, 149.47, 149.43, 149.36, 149.29, 149.19, 145.80, 126.49, 126.44, 126.41, 126.34, 123.08, 114.34, 114.28, 114.14, 114.08, 113.80, 113.74, 69.95, 69.86, 69.77, 69.50, 69.15, 68.81, 66.71, 62.86, 62.77, 62.67, 62.64, 30.59, 30.06, 30.02, 14.25. HRMS (m/z): $[C_{90}H_{114}O_{20}N_2Na]^+$ calcd. for $C_{90}H_{114}O_{20}N_2$, 1565.7857; found, 1565.7873.

3. NMR and HR-Mass Spectra

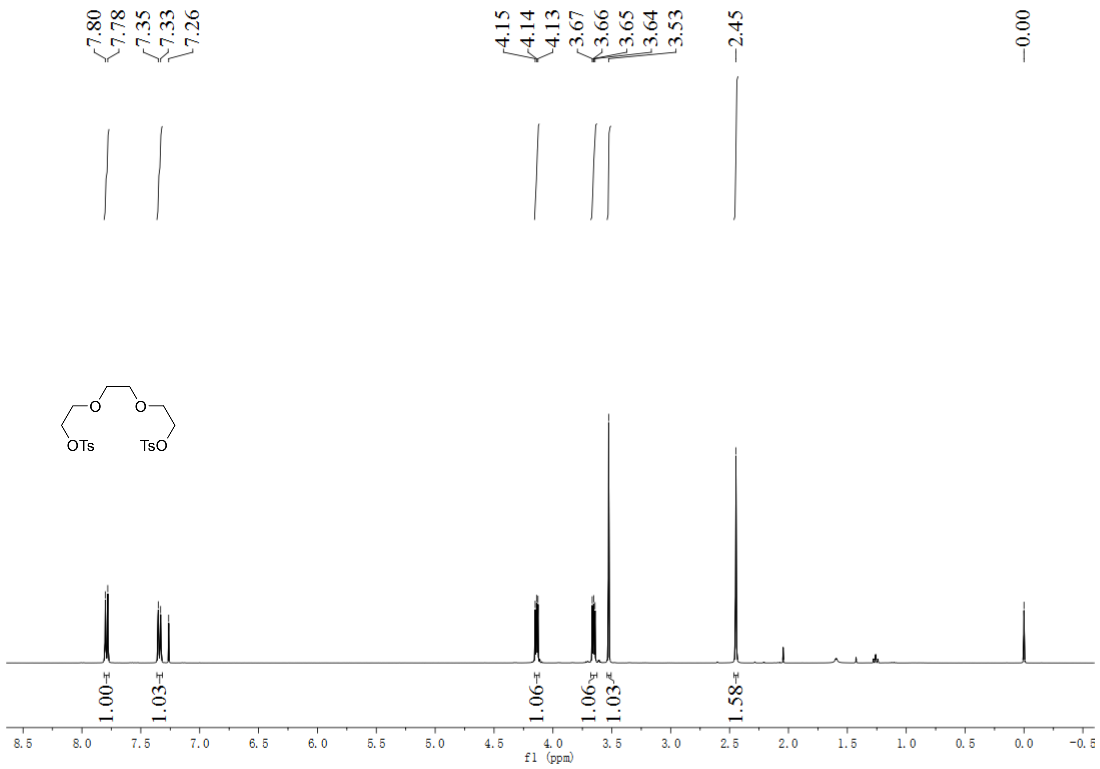

Supplementary Fig. 2 │ ^{1}H NMR spectrum of **1a** in chloroform-d at ambient temperature.

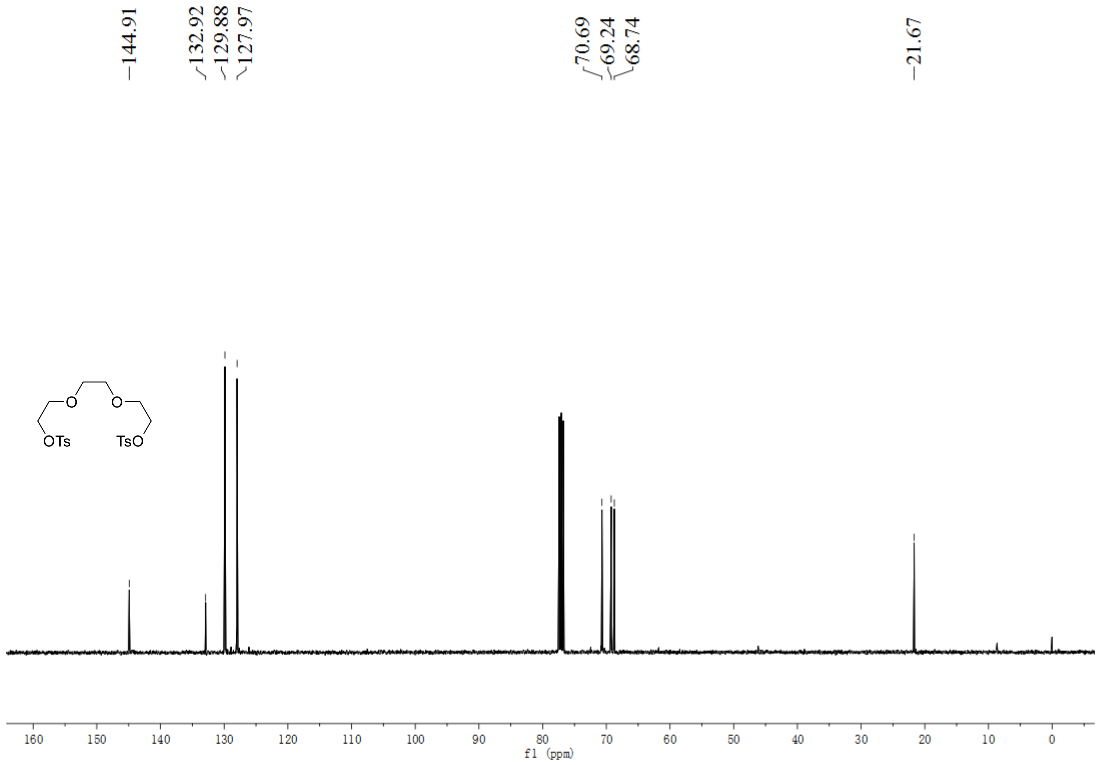

Supplementary Fig. 3 │ ^{13}C NMR spectrum of **1a** in chloroform-d at ambient temperature.

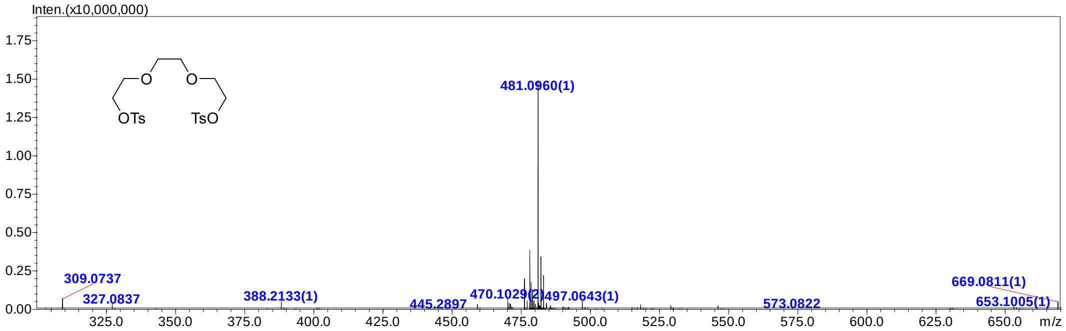

Supplementary Fig. 4 │ ESI-Mass spectrum of compound **1a**. ESI-MS: calcd ($[C_{20}H_{26}O_8S_2Na]^+$), *m/z* = 481.0961, found, *m/z* = 481.0960.

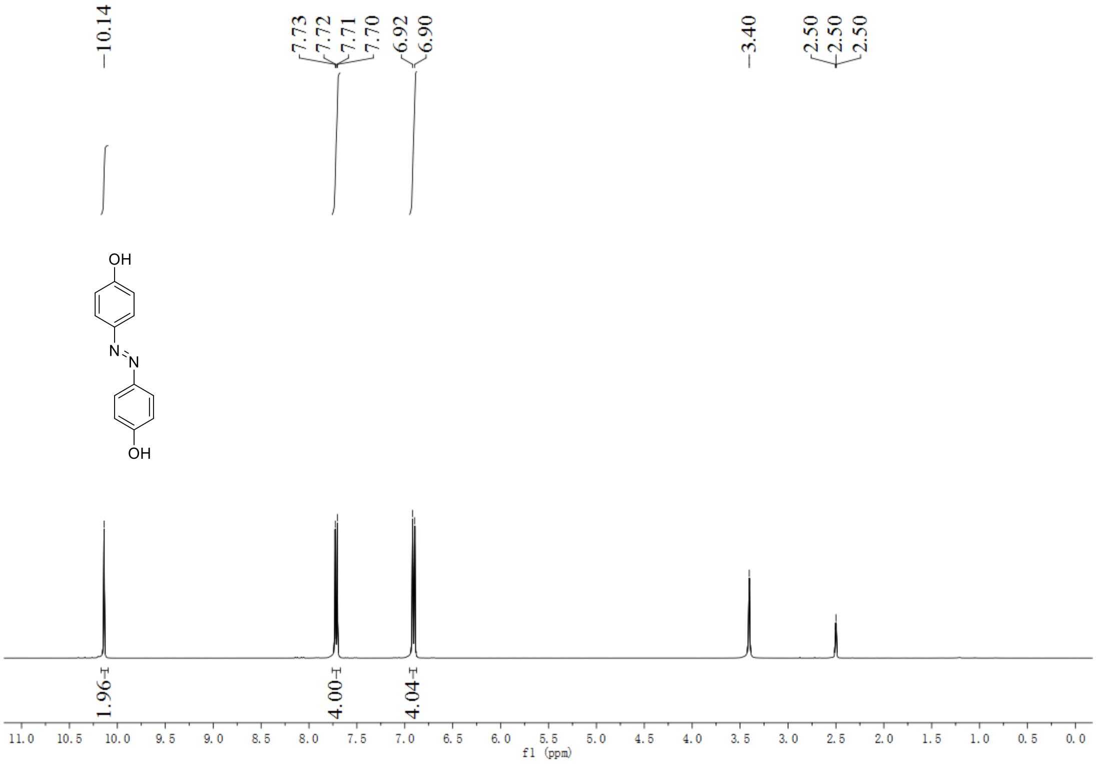

Supplementary Fig. 5 │ ^{1}H NMR spectrum of **2** in Methanol-d_4 at ambient temperature.

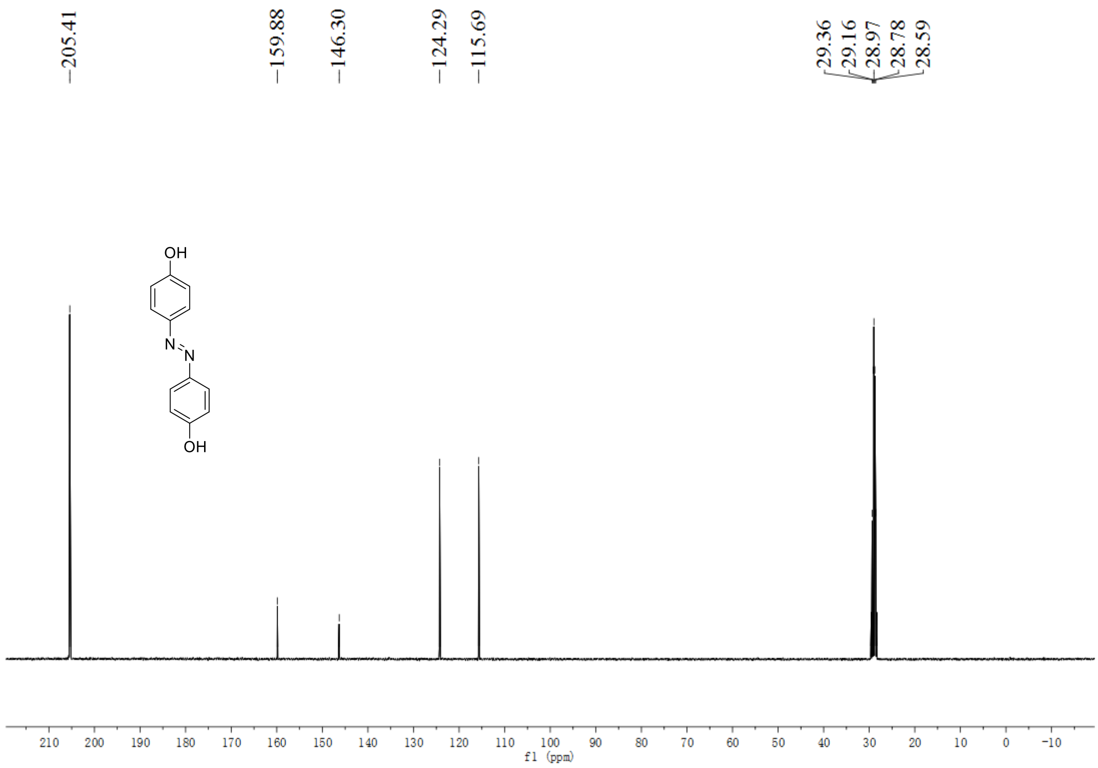

Supplementary Fig. 6 │ ^{13}C NMR spectrum of **2** in Methanol-d_4 at ambient temperature.

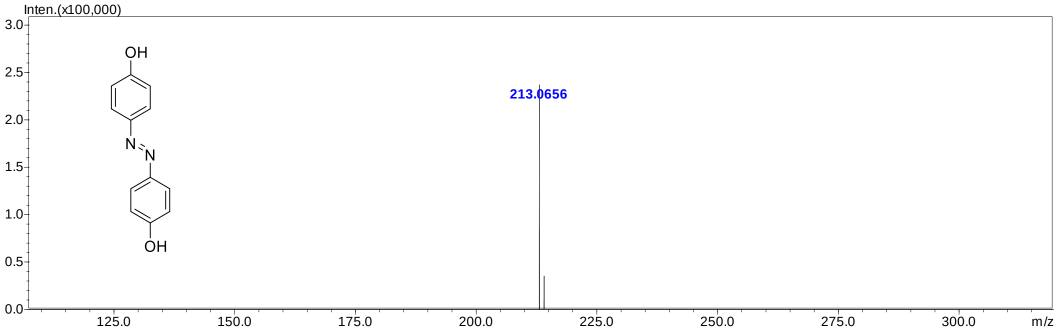

Supplementary Fig. 7 │ ESI-Mass spectrum of compound **2**. ESI-MS: calcd ($[C_{12}H_9N_2O_2]^-$), *m/z* = 213.0670, found, *m/z* = 213.0656.

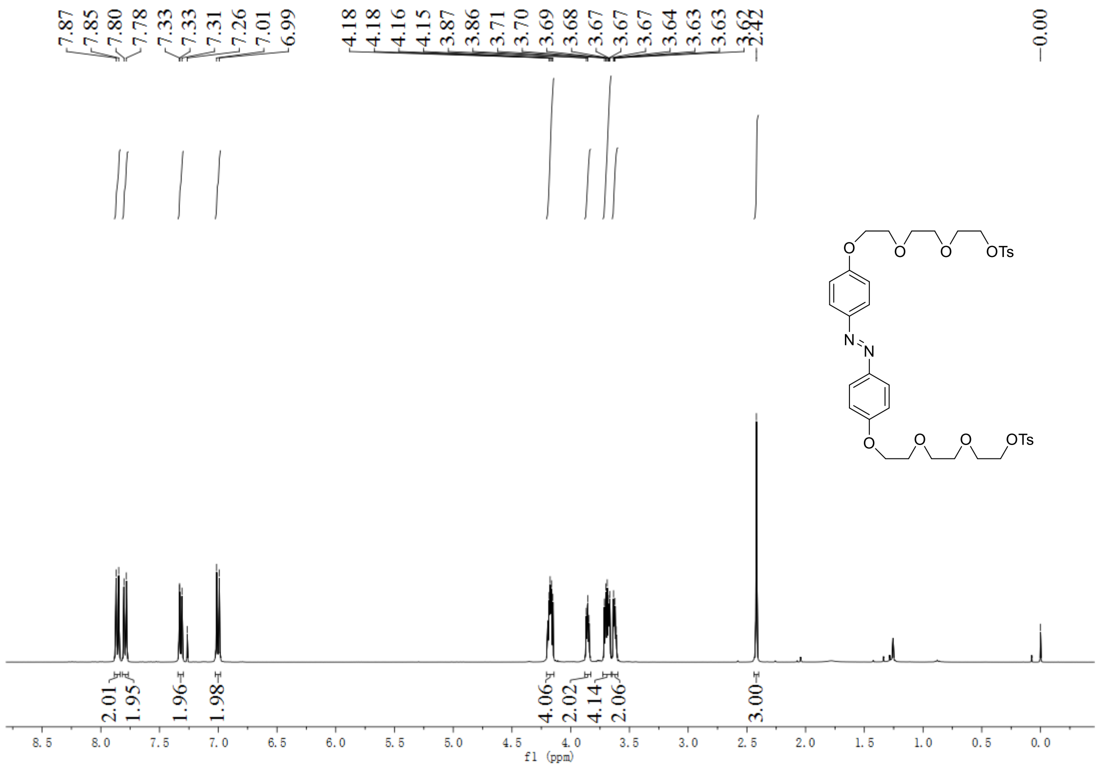

Supplementary Fig. 8 │ ^{1}H NMR spectrum of **3a** in chloroform-d at ambient temperature.

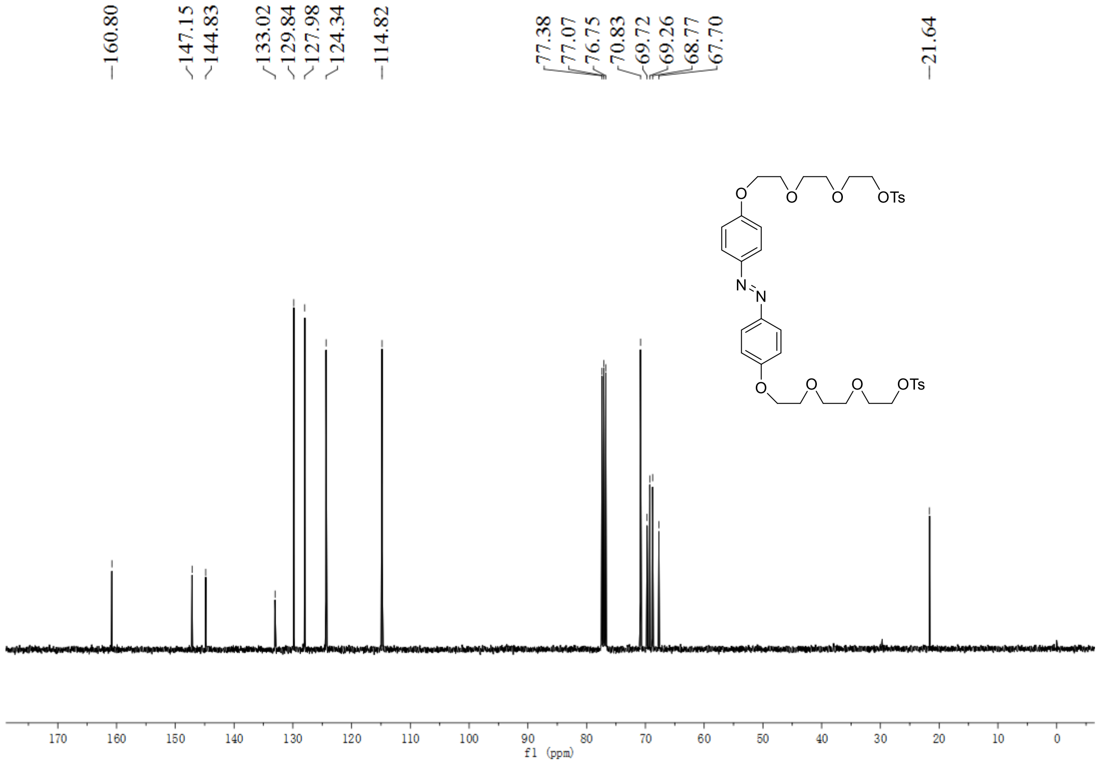

Supplementary Fig. 9 │ ^{13}C NMR spectrum of **3a** in chloroform-d at ambient temperature.

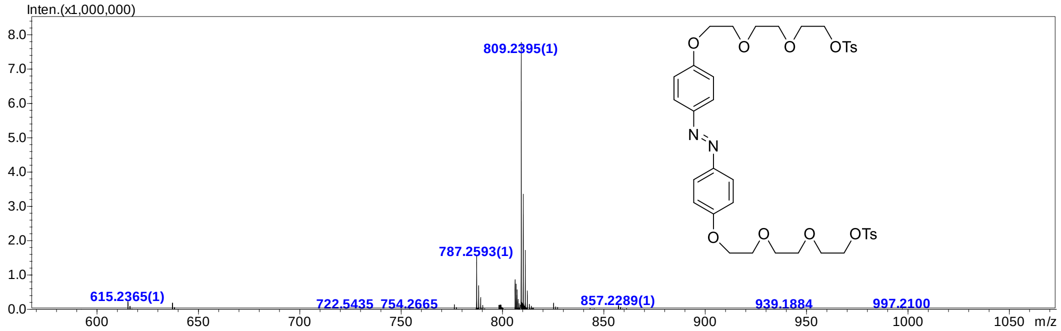

Supplementary Fig. 10 │ ESI-Mass spectrum of compound **3a**. ESI-MS: calcd ([$C_{38}H_{46}N_2O_{12}S_2Na$]$^+$), *m*/*z* = 809.2384, found, *m*/*z* = 809.2395.

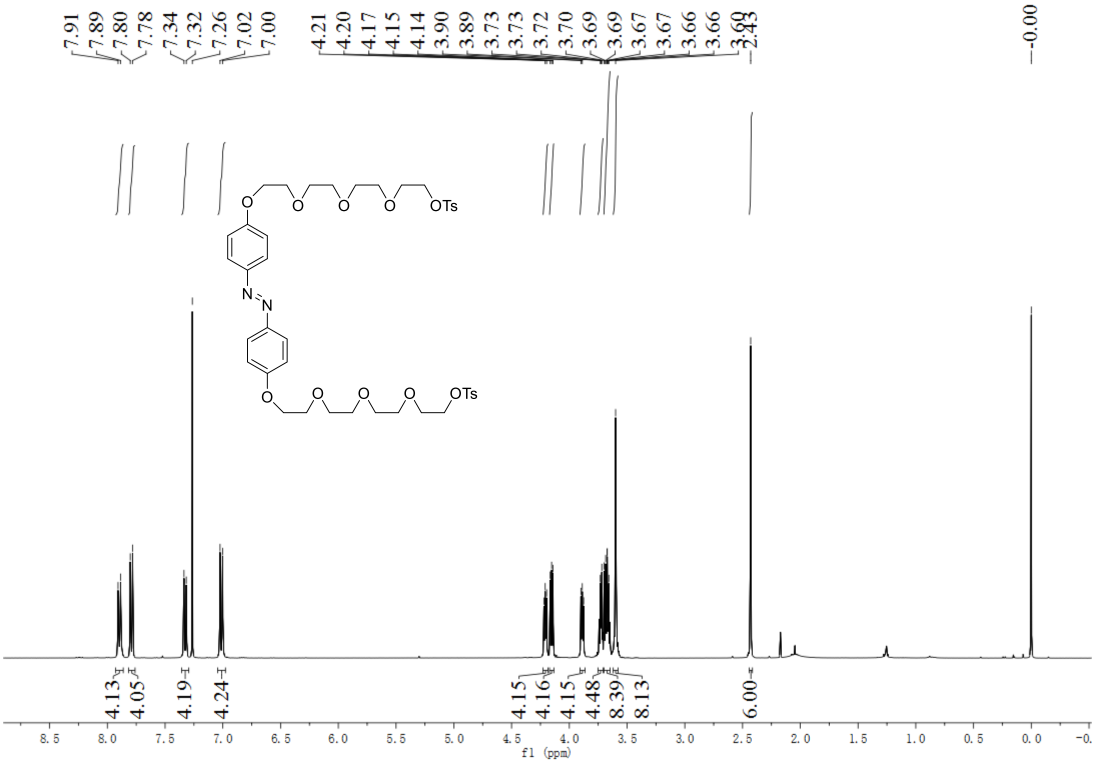

Supplementary Fig. 11 │ ^{1}H NMR spectrum of **3b** in chloroform-d at ambient temperature.

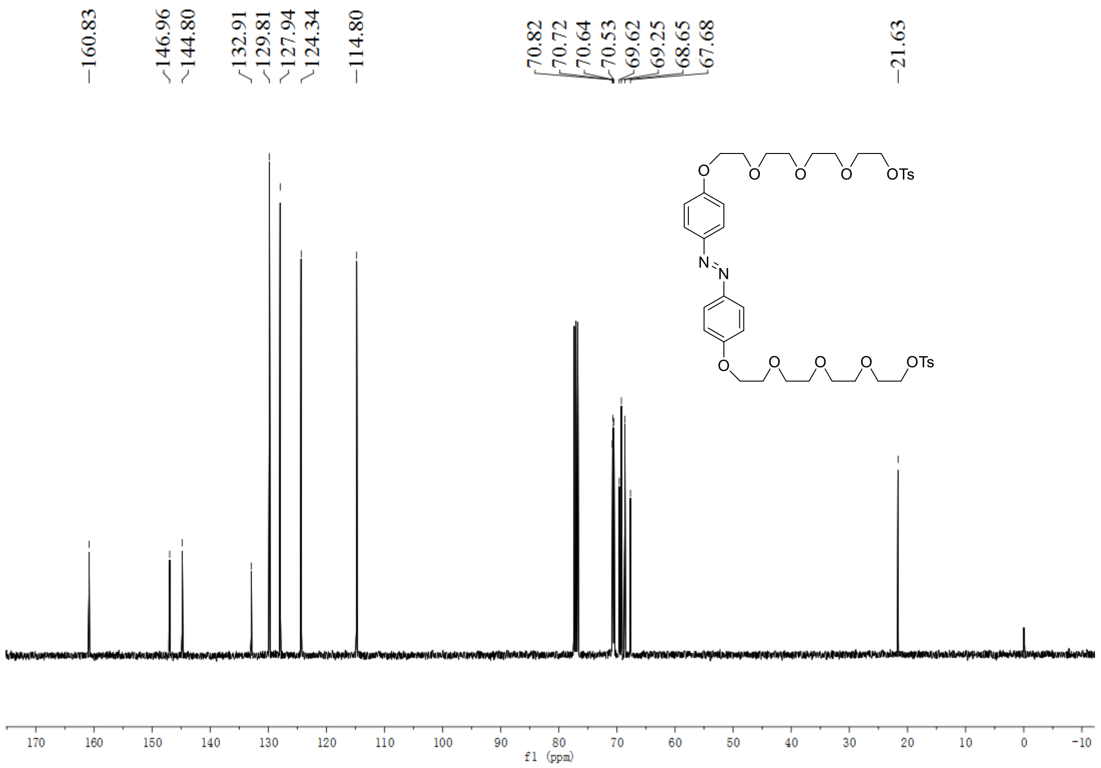

Supplementary Fig. 12 │ ^{13}C NMR spectrum of **3b** in chloroform-d at ambient temperature.

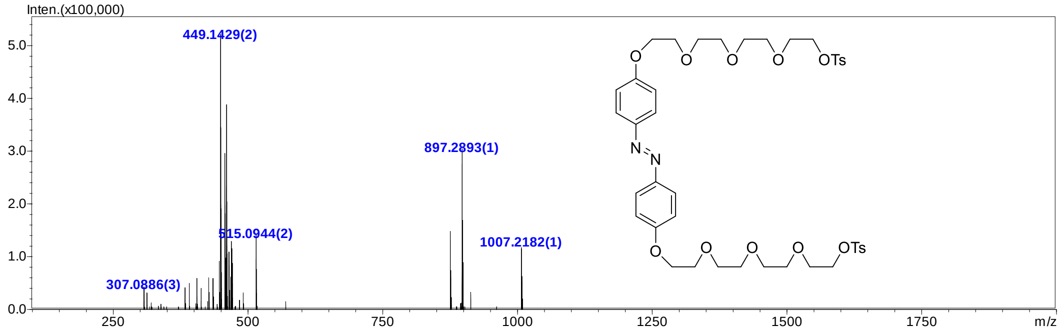

Supplementary Fig. 13 │ ESI-Mass spectrum of compound **3b**. ESI-MS: calcd ([$C_{42}H_{54}N_2O_{14}S_2Na$]$^+$), m/z = 897.2909, found, m/z = 897.2893.

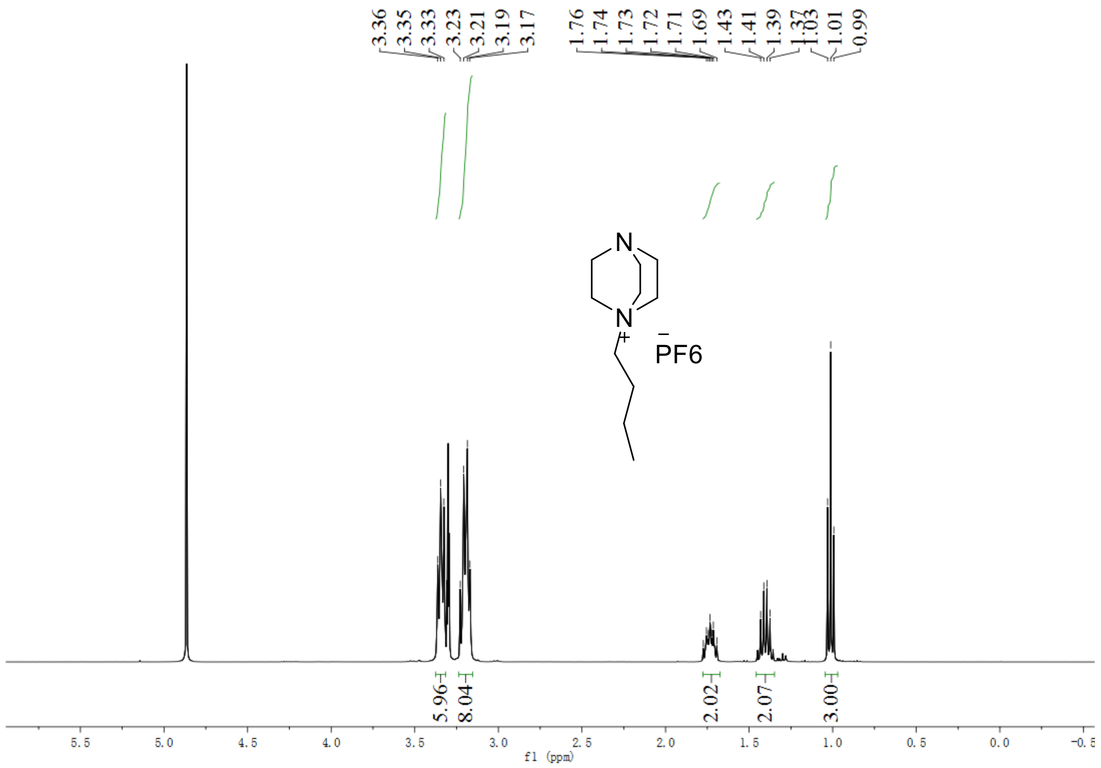

Supplementary Fig. 14 │ ^{1}H NMR spectrum of **G1** in chloroform-d at ambient temperature.

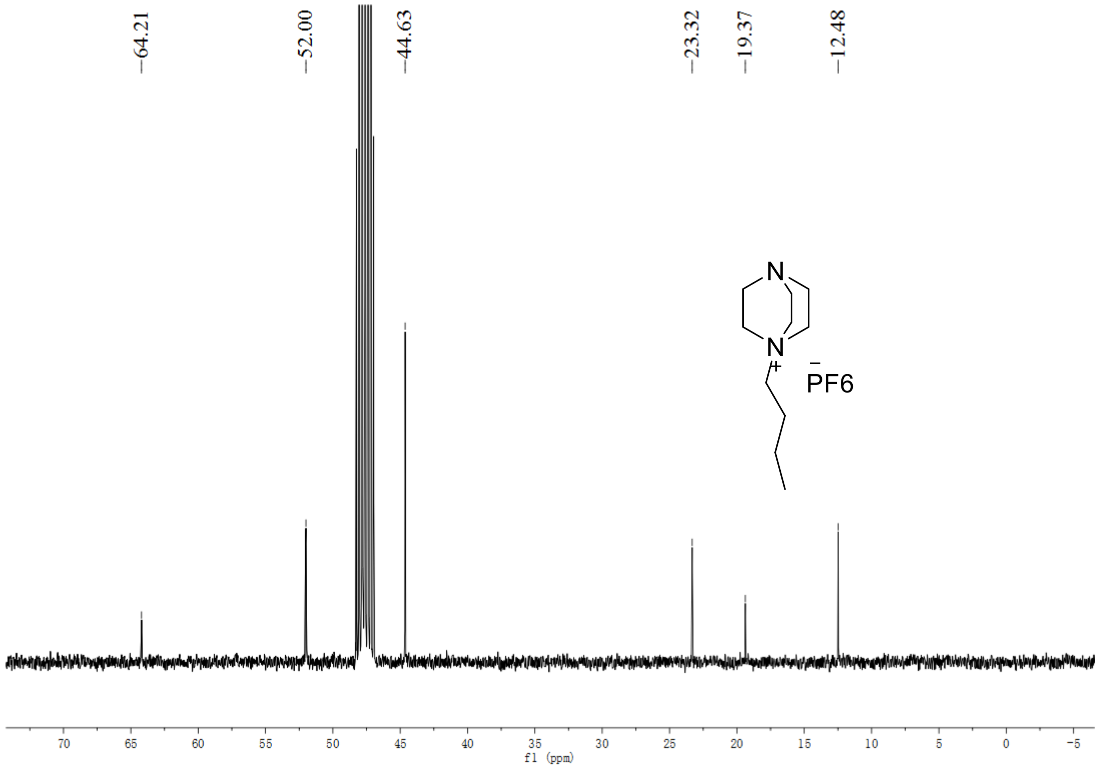

Supplementary Fig. 15 │ ^{13}C NMR spectrum of **G1** in chloroform-d at ambient temperature.

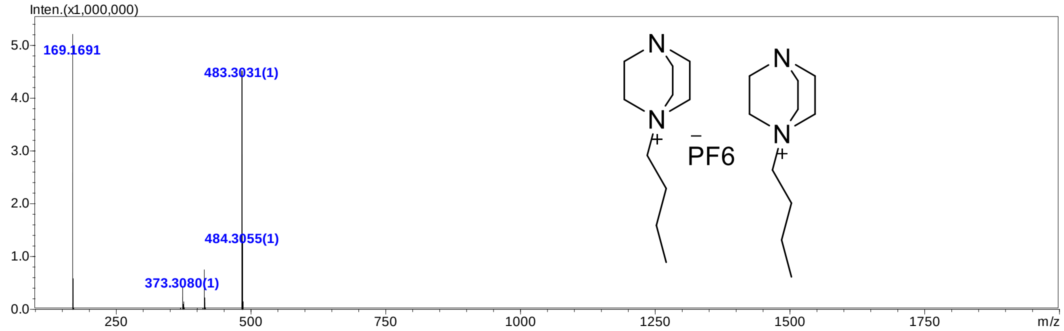

Supplementary Fig. 16 │ ESI-Mass spectrum of compound **G1**. ESI-MS: calcd ($[C_{20}H_{42}N_4FP_6]^+$), m/z = 483.3046, found, m/z = 483.3031.

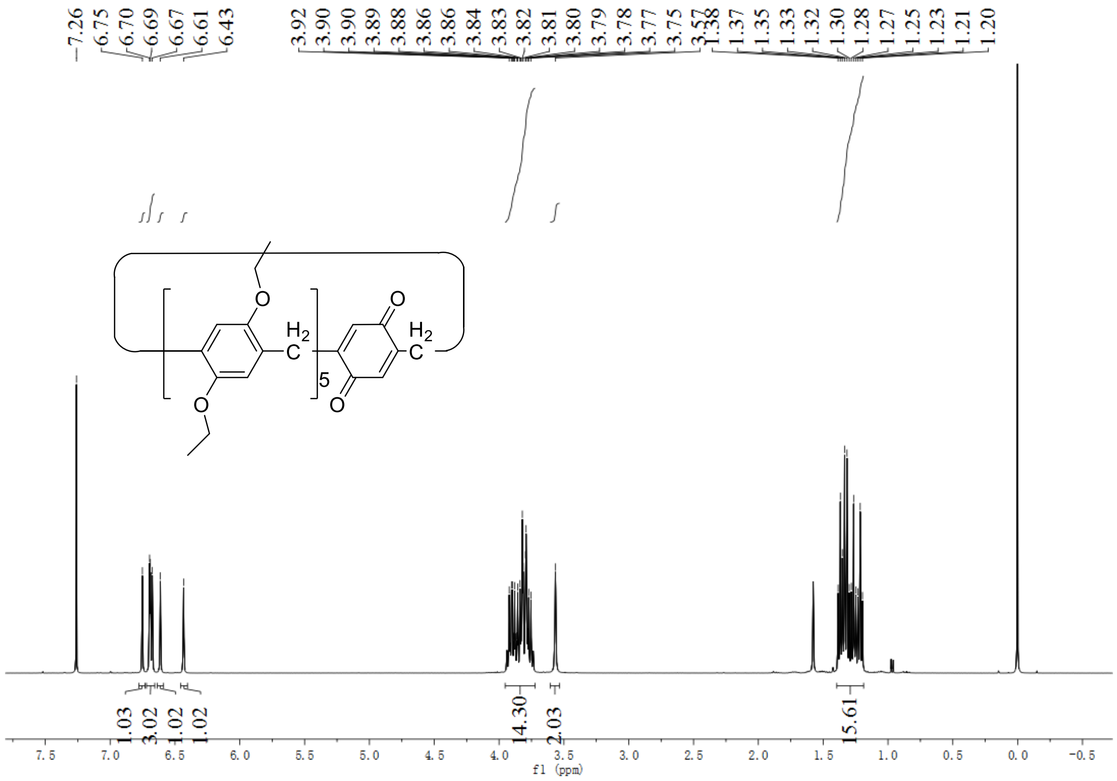

Supplementary Fig. 17 │ ^{1}H NMR spectrum of **4** in chloroform-d at ambient temperature.

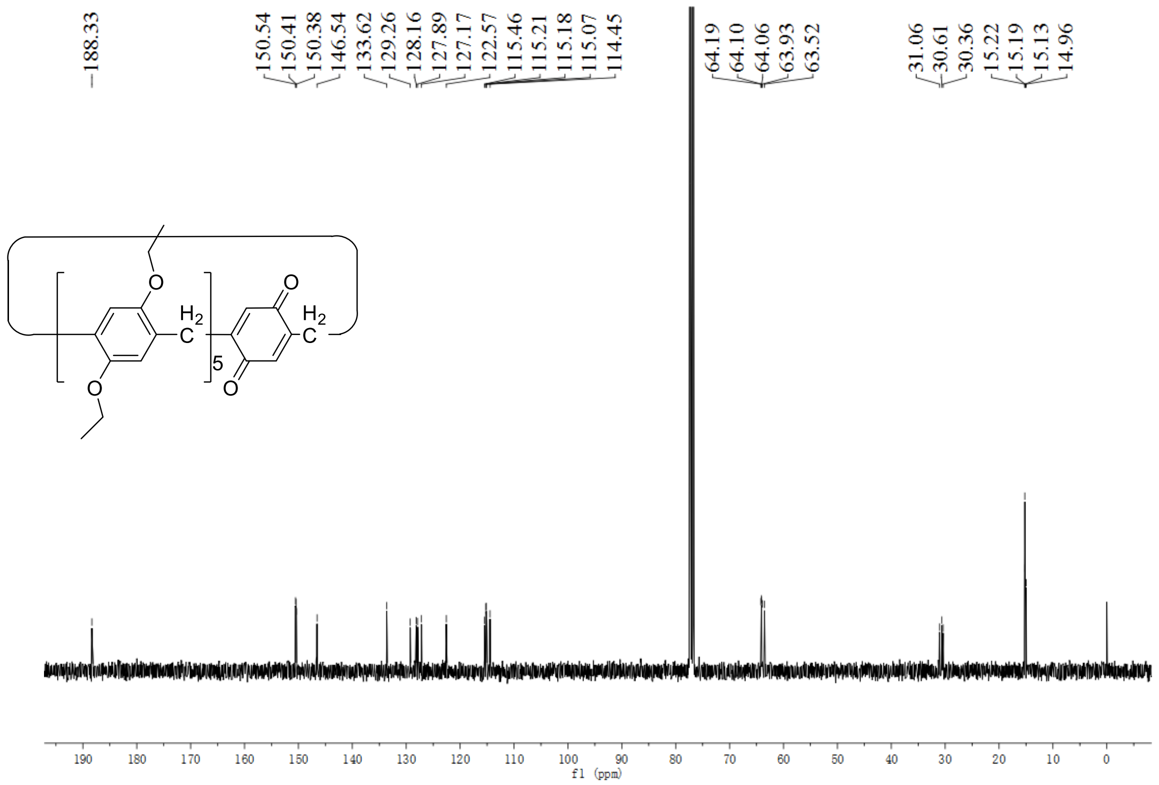

Supplementary Fig. 18 │ ^{13}C NMR spectrum of **4** in chloroform-d at ambient temperature.

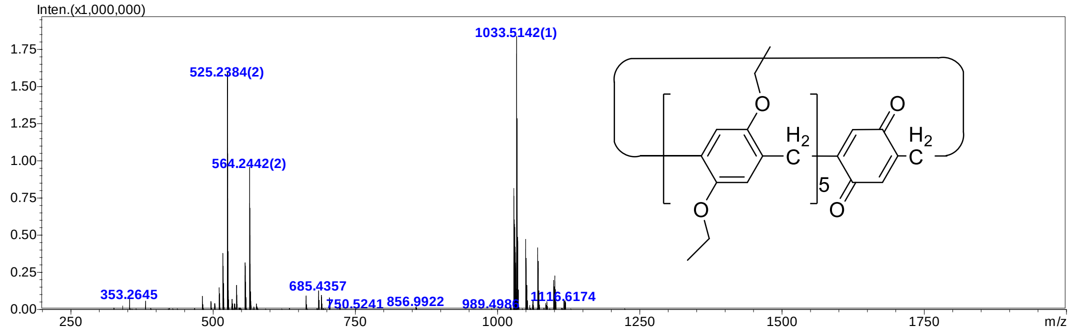

Supplementary Fig. 19 │ ESI-Mass spectrum of compound **4**. ESI-MS: calcd ($[C_{62}H_{74}O_{12}Na]^+$), *m/z* = 1033.5072, found, *m/z* = 1033.5142.

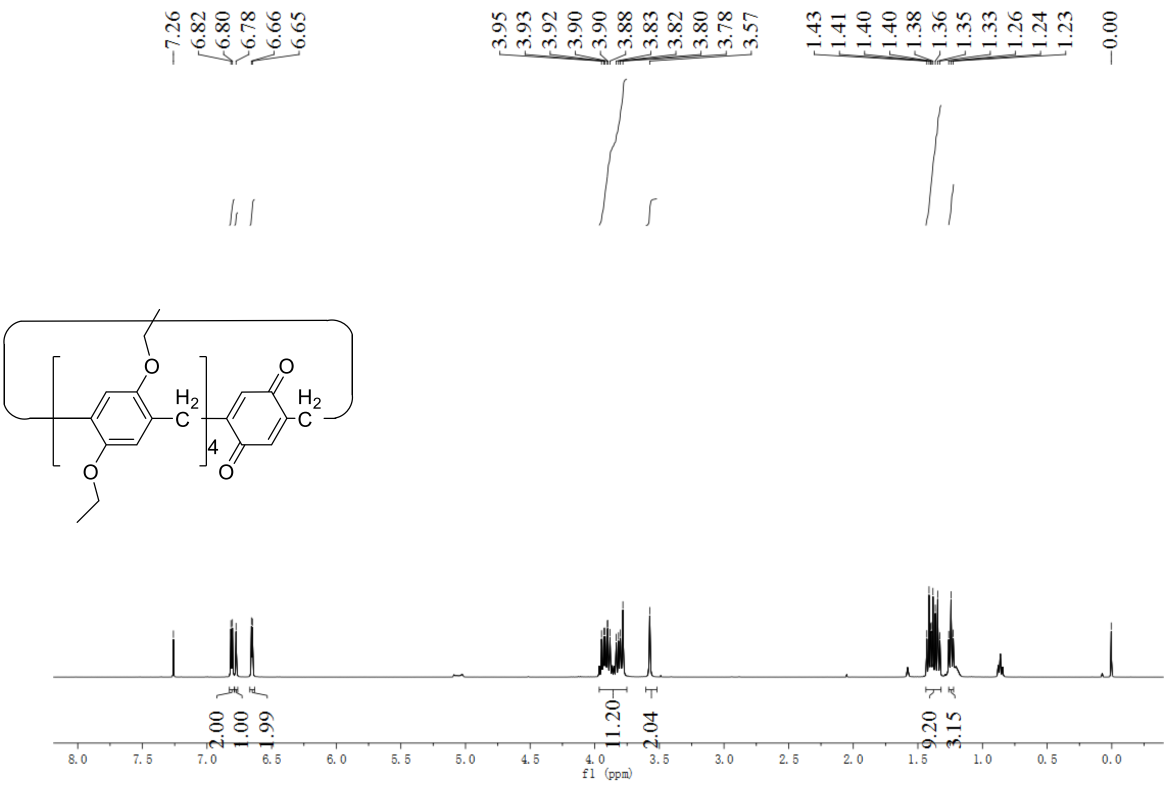

Supplementary Fig. 20 | **^{1}H NMR spectrum of **5 in $CDCl_3$ at ambient temperature.

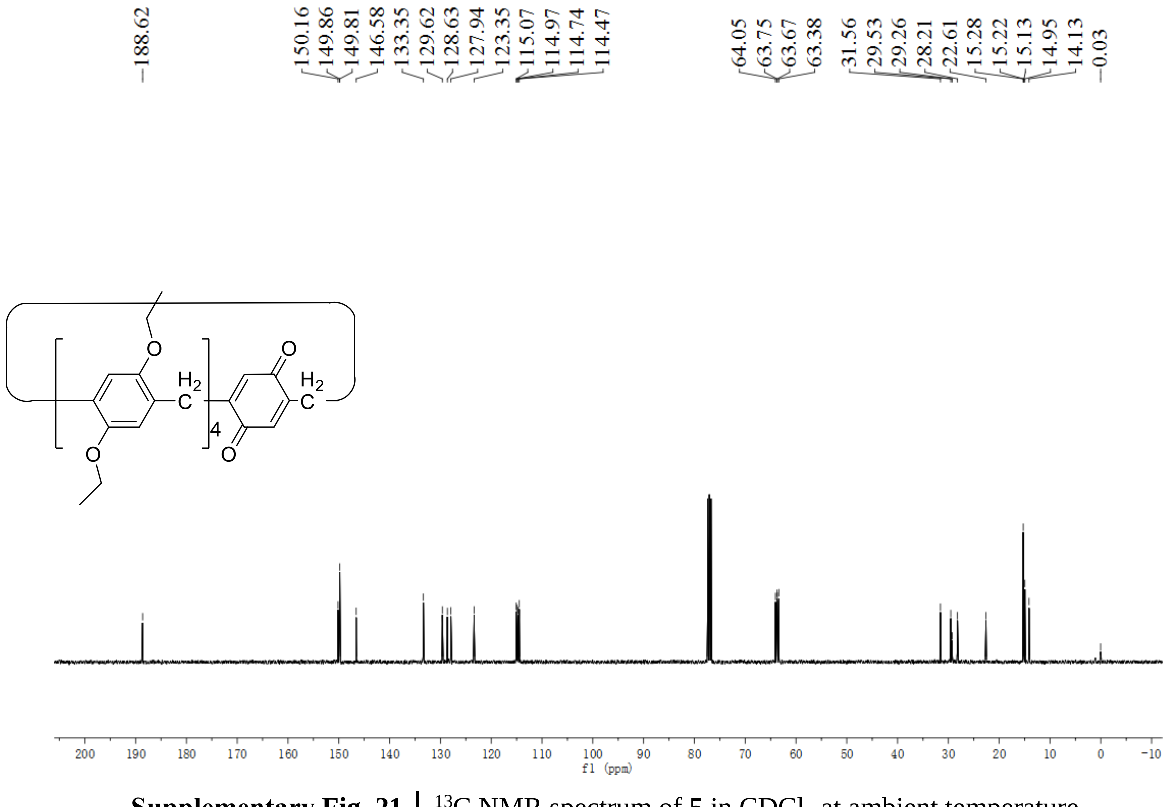

Supplementary Fig. 21 | **^{13}C NMR spectrum of **5 in $CDCl_3$ at ambient temperature.

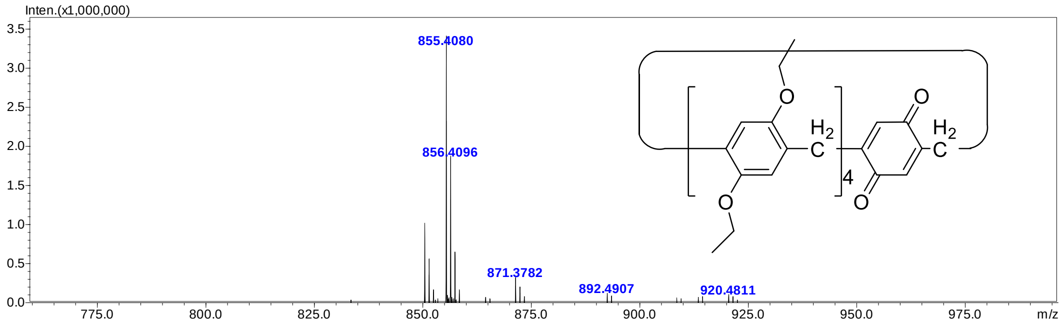

Supplementary Fig. 22 │ ESI-Mass spectrum of compound **5**. ESI-MS: calcd ($[C_{51}H_{60}O_{10}Na]^+$), *m*/*z* = 855.4079, found, *m*/*z* = 855.4080.

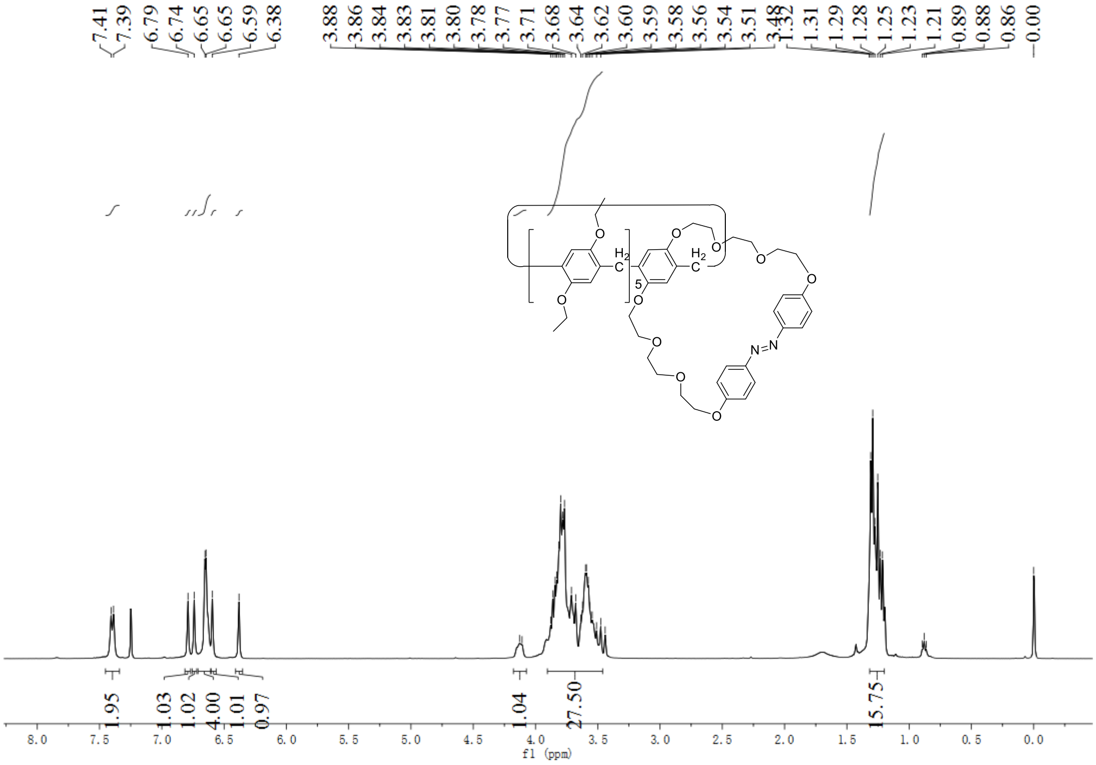

Supplementary Fig. 23 │ ^{1}H NMR spectrum of **MUJ1** in $CDCl_3$ at ambient temperature.

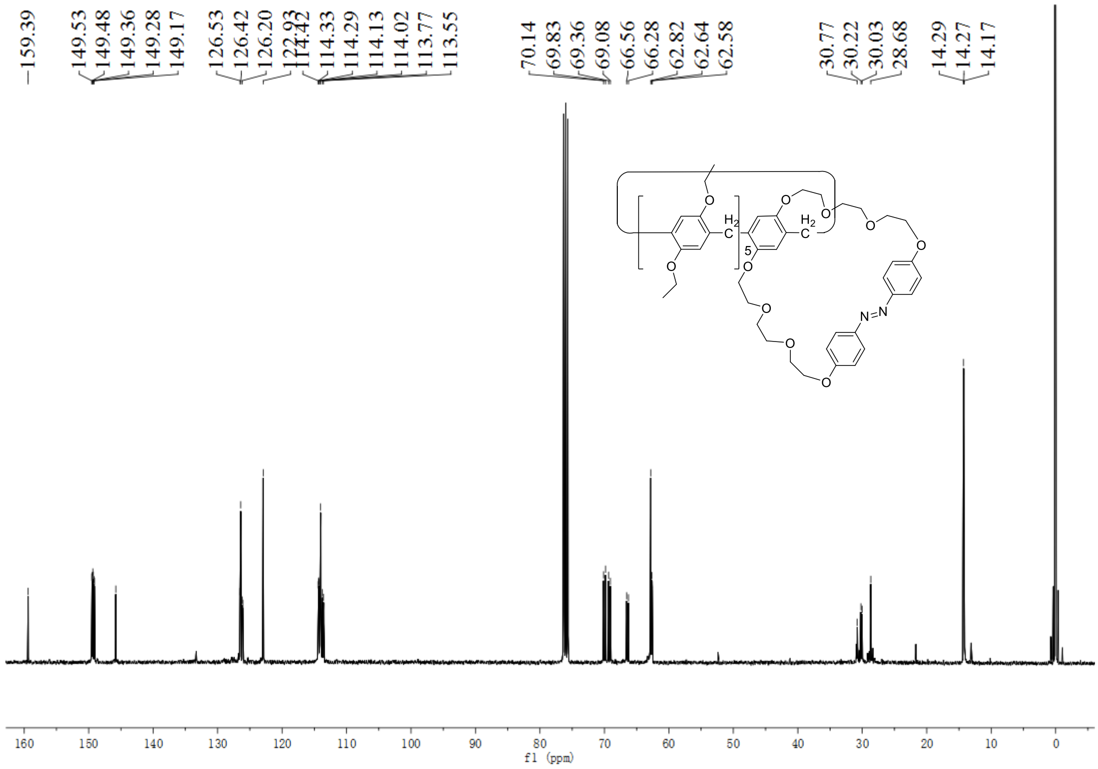

Supplementary Fig. 24 │ ^{13}C NMR spectrum of **MUJ1** in $CDCl_3$ at ambient temperature.

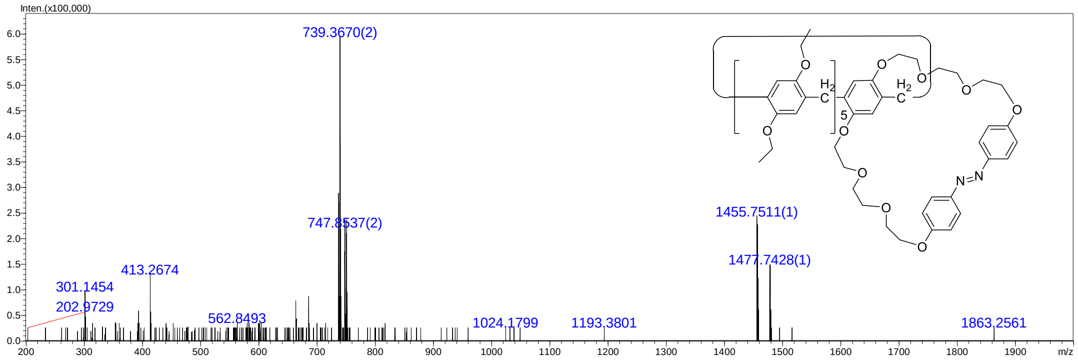

Supplementary Fig. 25 │ ESI-Mass spectrum of compound **MUJ1**. ESI-MS: calcd ($[C_{86}H_{106}O_{18}N_2H]^+$), m/z = 1455.7513, found, m/z = 1455.7511.

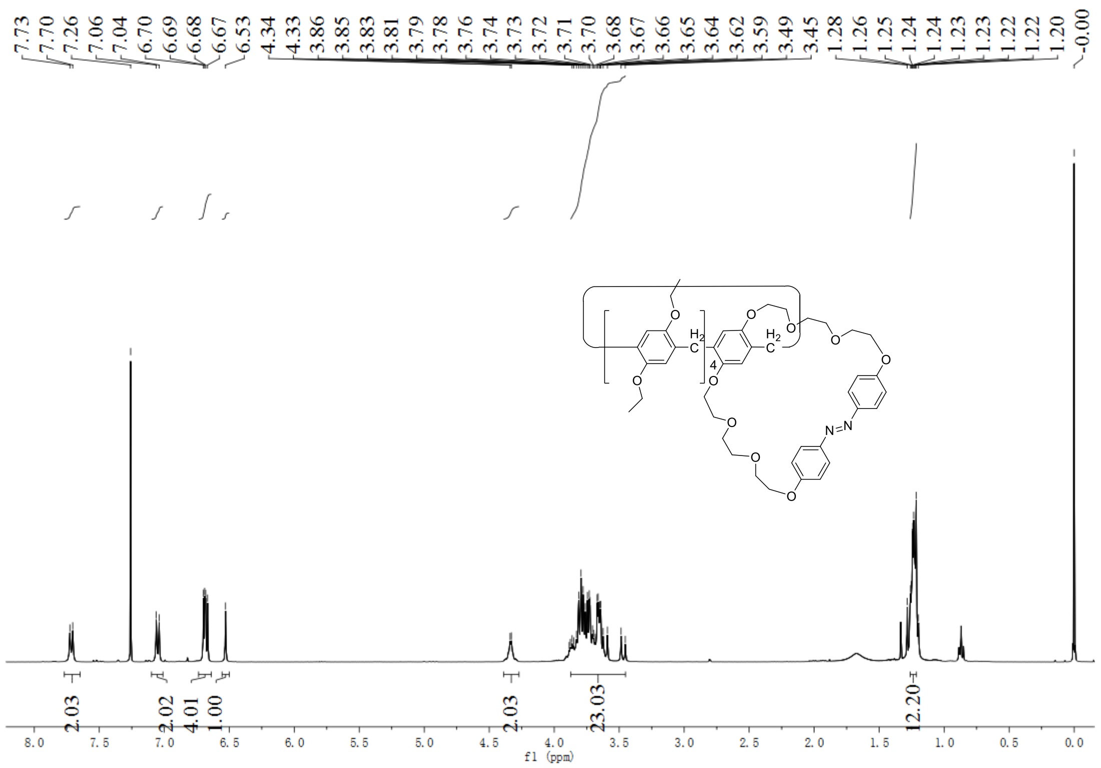

Supplementary Fig. 26 │ ^{1}H NMR spectrum of **MUJ2** in $CDCl_3$ at ambient temperature.

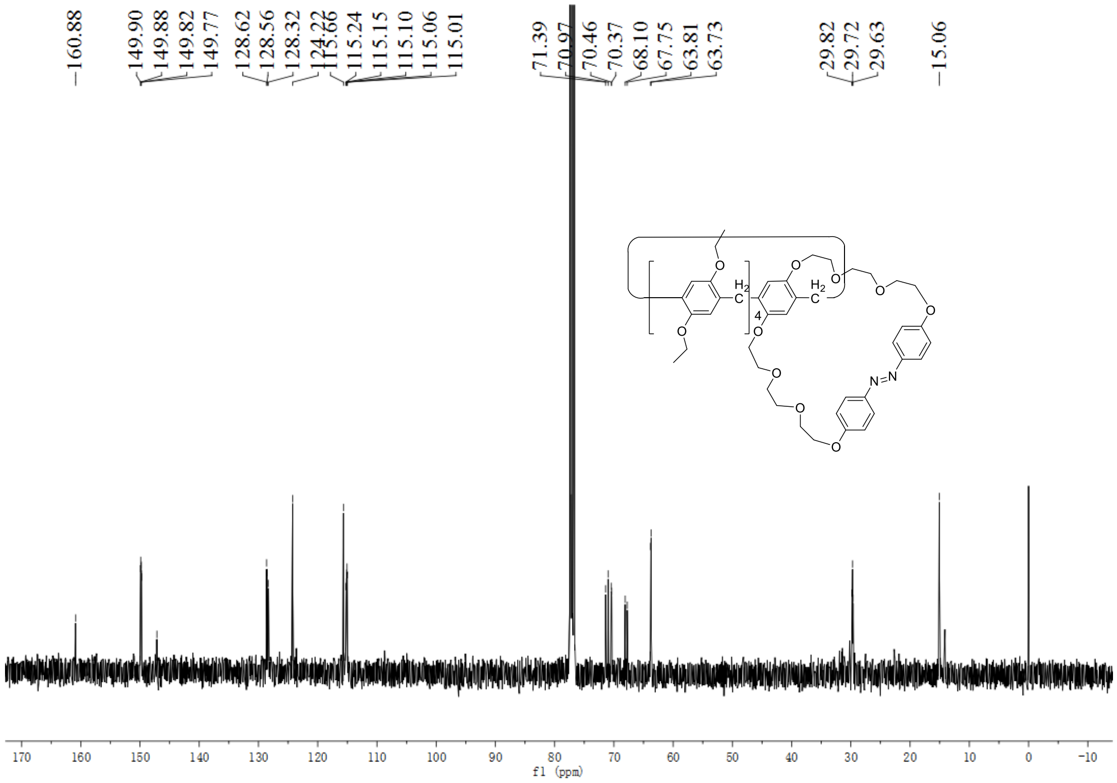

Supplementary Fig. 27 │ ^{13}C NMR spectrum of **MUJ2** in $CDCl_3$ at ambient temperature.

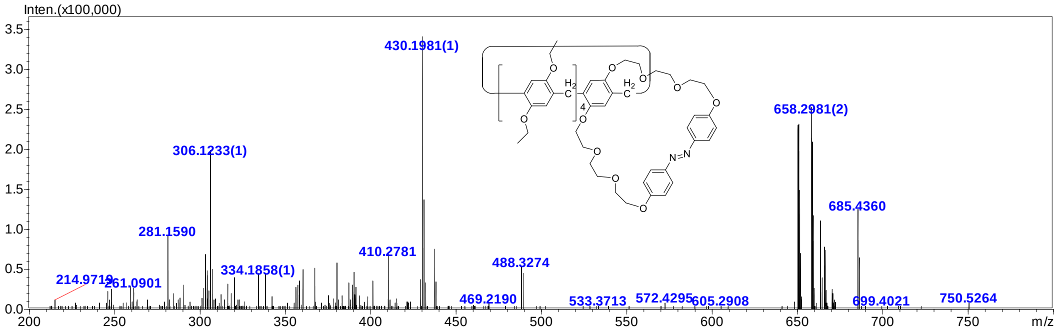

Supplementary Fig. 28 │ ESI-Mass spectrum of compound **MUJ2**. ESI-MS: calcd ($[C_{75}H_{92}O_{16}N_2HK]^{2+}$), *m*/*z* = 658.3076, found, *m*/*z* = 658.2981.

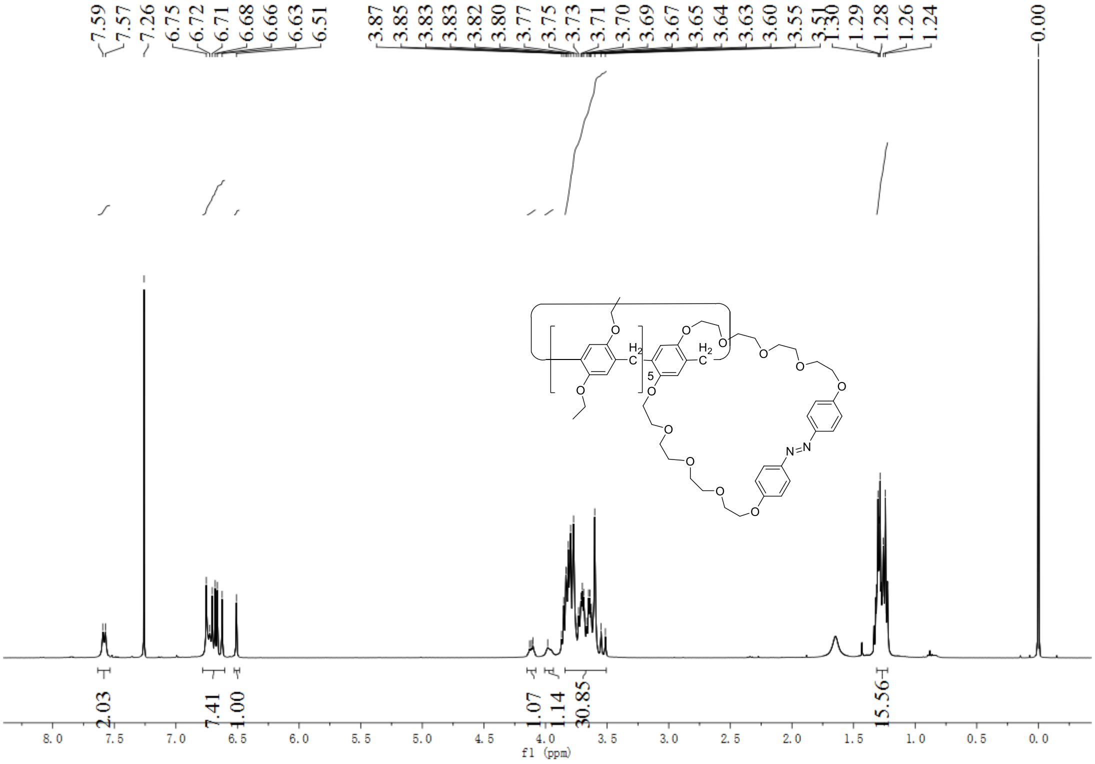

Supplementary Fig. 29 │ ^{1}H NMR spectrum of **MUJ3** in $CDCl_3$ at ambient temperature.

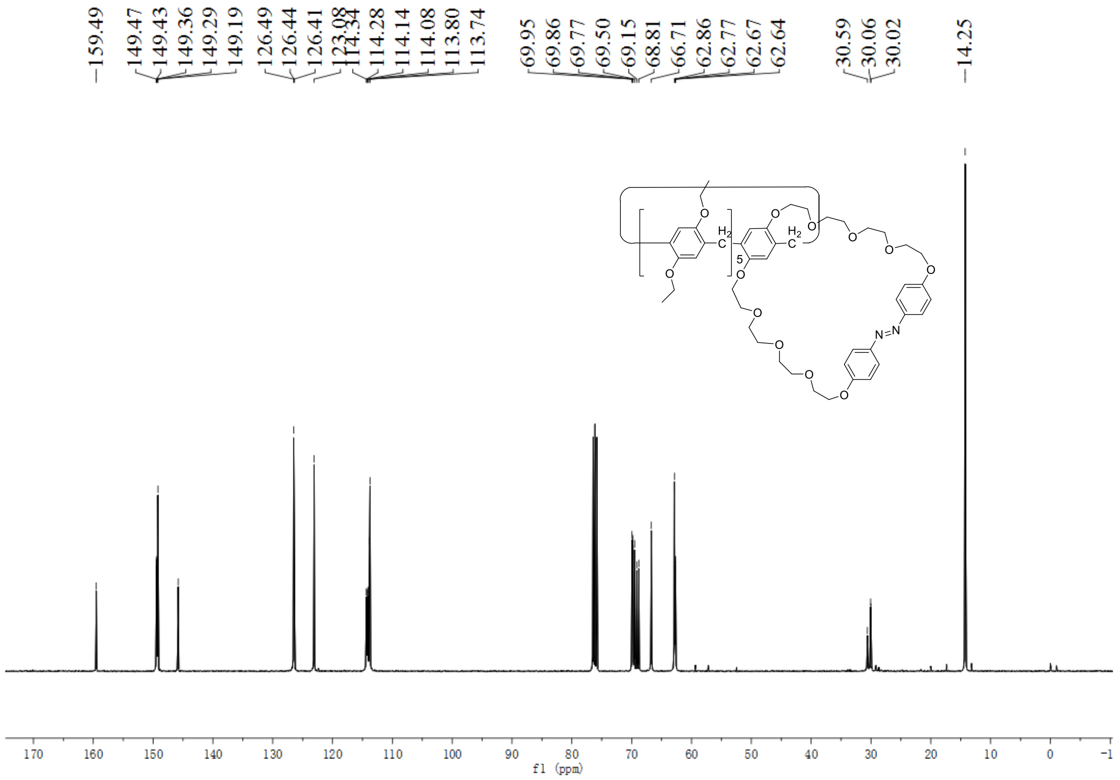

Supplementary Fig. 30 │ ^{13}C NMR spectrum of **MUJ3** in $CDCl_3$ at ambient temperature.

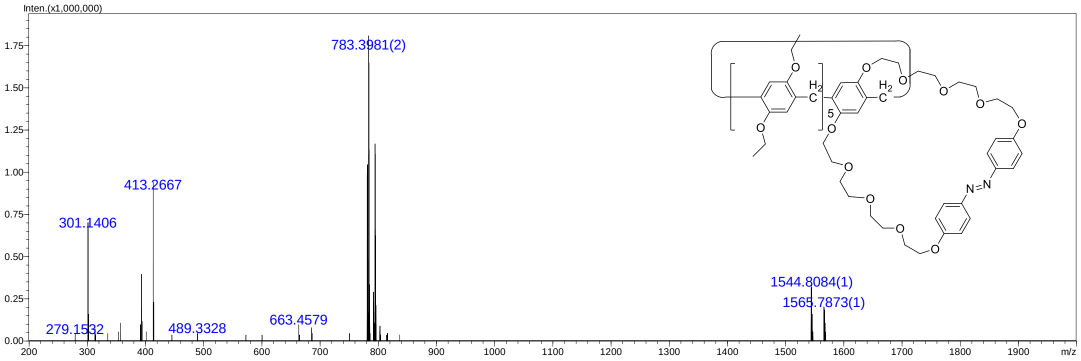

Supplementary Fig. 31 │ ESI-Mass spectrum of compound **MUJ3**. ESI-MS: calcd ($[C_{90}H_{114}O_{20}N_2Na]^+$), *m/z* = 1565.7857, found, *m/z* = 1565.7873.

4. Isoabsorptive Point of MUJs

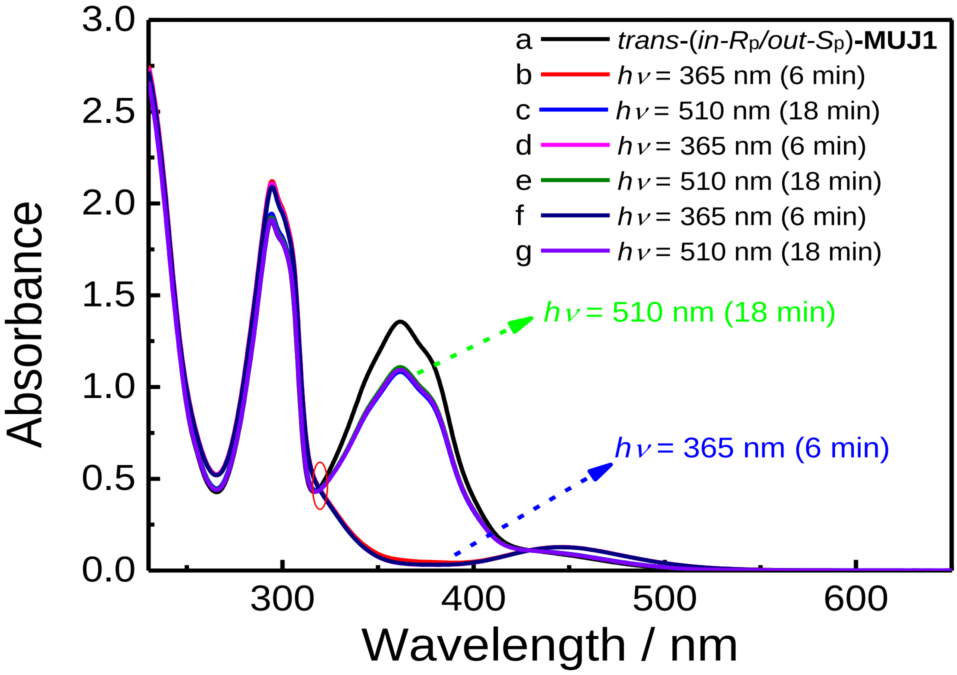

Supplementary Fig. 32 │ UV-vis absorption spectral changes (tetrahydrofuran : *n*-hexane = 1 : 4) of *trans*-(*in*-R_p/*out*-S_p)-**MUJ1** upon irradiation at 365 nm and 510 nm.

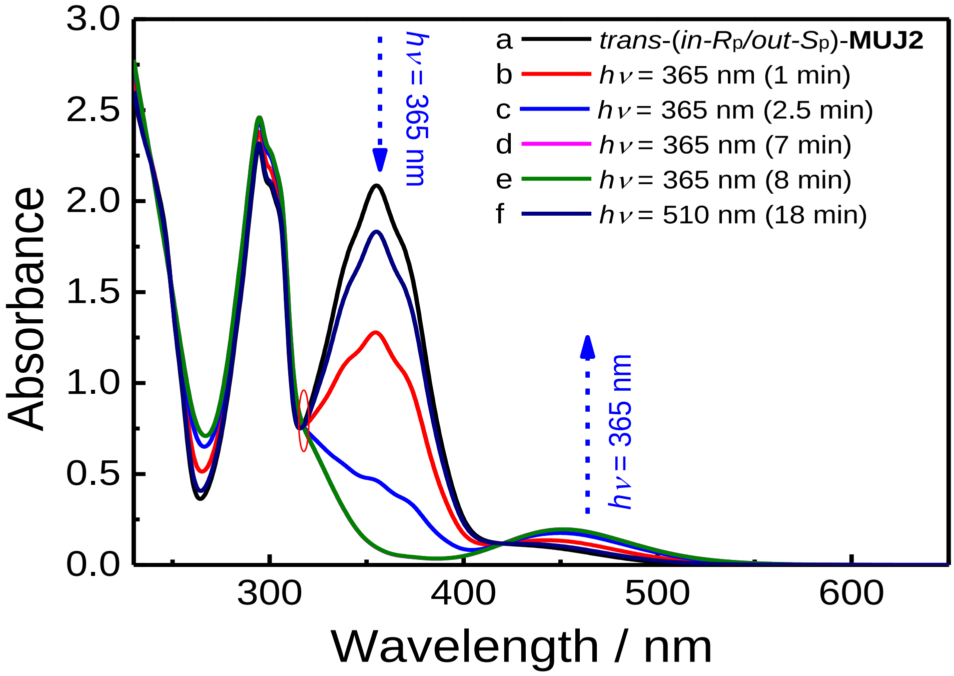

Supplementary Fig. 33 │ UV-vis absorption spectral changes (tetrahydrofuran : *n*-hexane = 3 : 17) of *trans*-(*in*-S_p/*out*-R_p)-**MUJ2** upon irradiation at 365 nm.

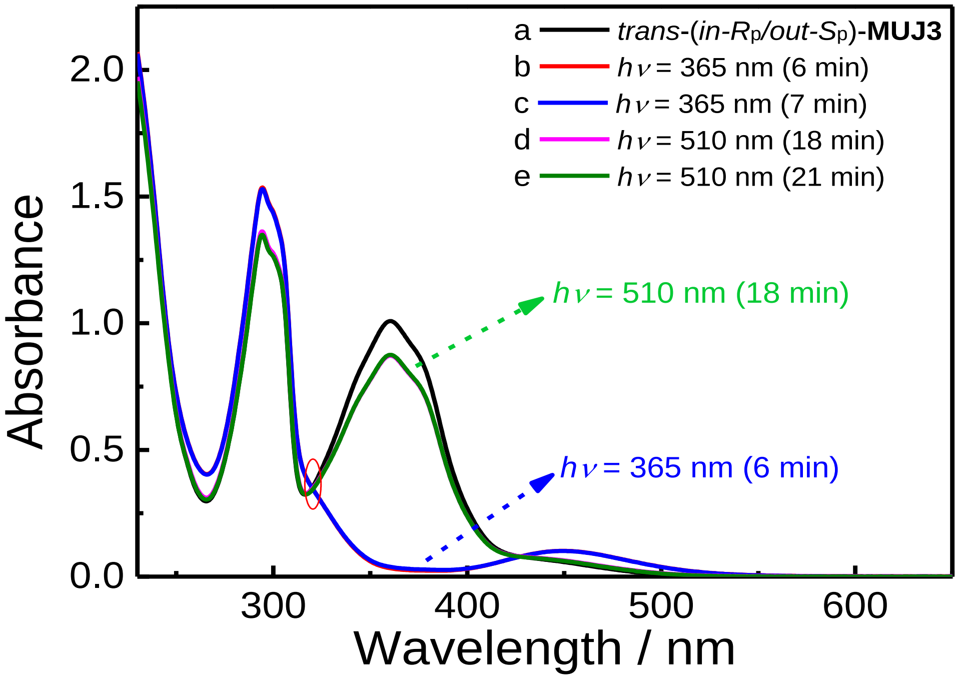

Supplementary Fig. 34 | UV-vis absorption spectral changes (tetrahydrofuran : *n*-hexane = 1 : 4) of *trans*-(*in*-R_p/*out*-S_p)-**MUJ3** upon irradiation at 365 nm and 510 nm.

5. Chiral HPLC Analysis

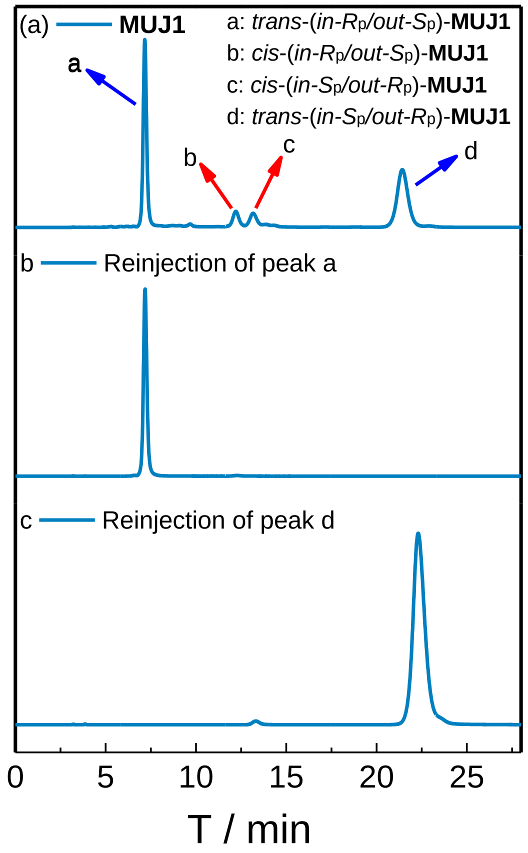

Supplementary Fig. 35 │ Chiral HPLC traces (*n*-hexane : tetrahydrofuran = 4 : 1, 319 nm) of (a) **MUJ1**, (b) *trans*-(*in*-R_p/*out*-S_p)-**MUJ1**, and (b) *trans*-(*in*-S_p/*out*-R_p)-**MUJ1**.

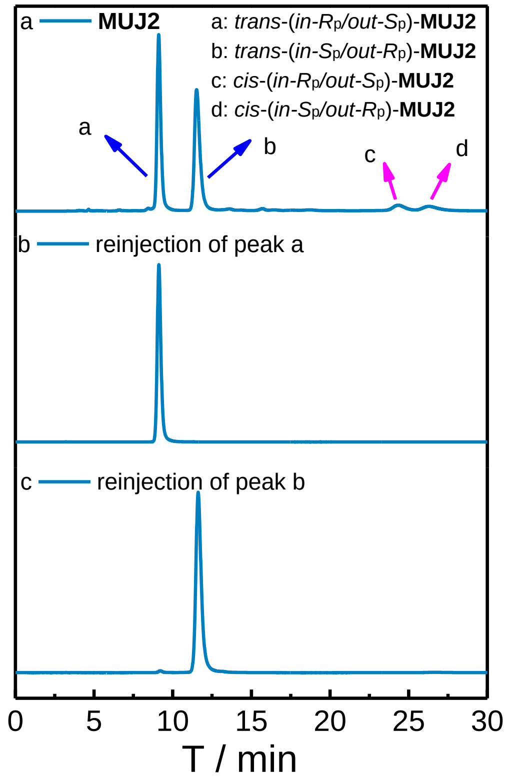

Supplementary Fig. 36 │ Chiral HPLC traces (*n*-hexane : tetrahydrofuran = 17 : 3, 317 nm) of (a) **MUJ2**, (b) *trans*-(*in*-R_p/*out*-S_p)-**MUJ2**, and (c) *trans*-(*in*-S_p/*out*-R_p)-**MUJ2**.

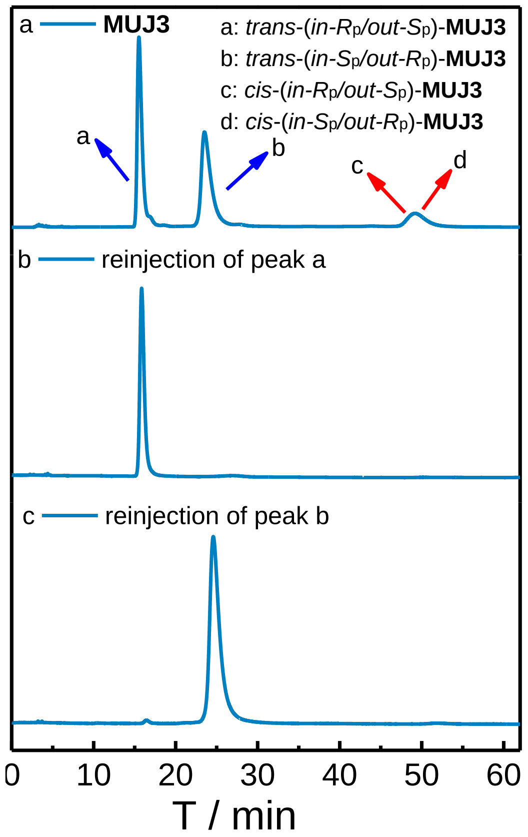

Supplementary Fig. 37 │ Chiral HPLC traces (*n*-hexane : tetrahydrofuran = 4 : 1, 320 nm) of (a) **MUJ3**, (b) *trans*-(*in*-R_p/*out*-S_p)-**MUJ3**, and (c) *trans*-(*in*-S_p/*out*-R_p)-**MUJ3**.

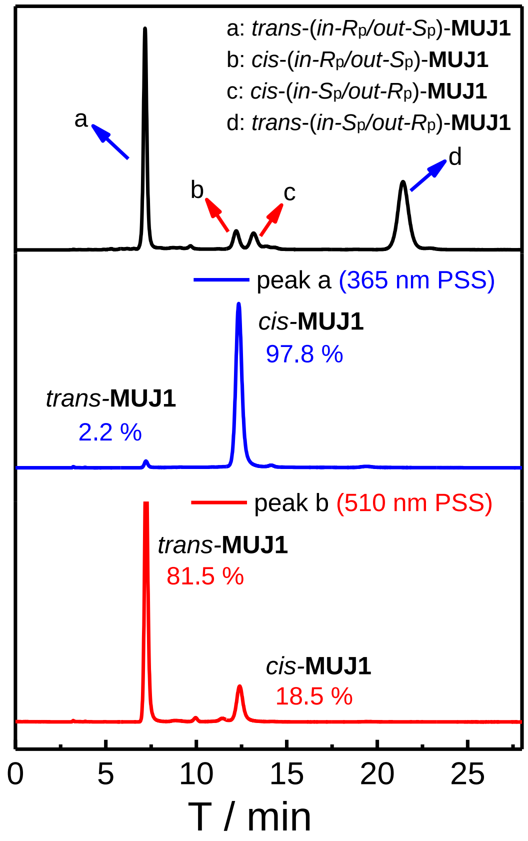

Supplementary Fig. 38 │ Chiral HPLC traces (tetrahydrofuran : *n*-hexane = 1 : 4, 319 nm) of **MUJ1** (top) and (*in*-R_p/*out*-S_p)-**MUJ1** in the PSS at 365 nm (middle) and 510 nm (bottom).

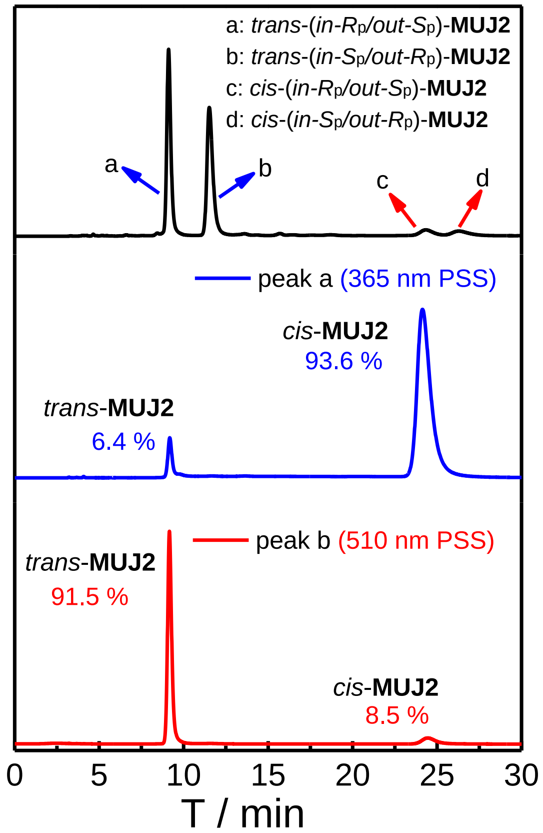

Supplementary Fig. 39 │ Chiral HPLC traces (*n*-hexane : tetrahydrofuran = 17 : 3, 317 nm) of **MUJ2** (top) and (*in*-R_p/*out*-S_p)-**MUJ2** in the PSS at 365 nm (middle) and 510 nm (bottom).

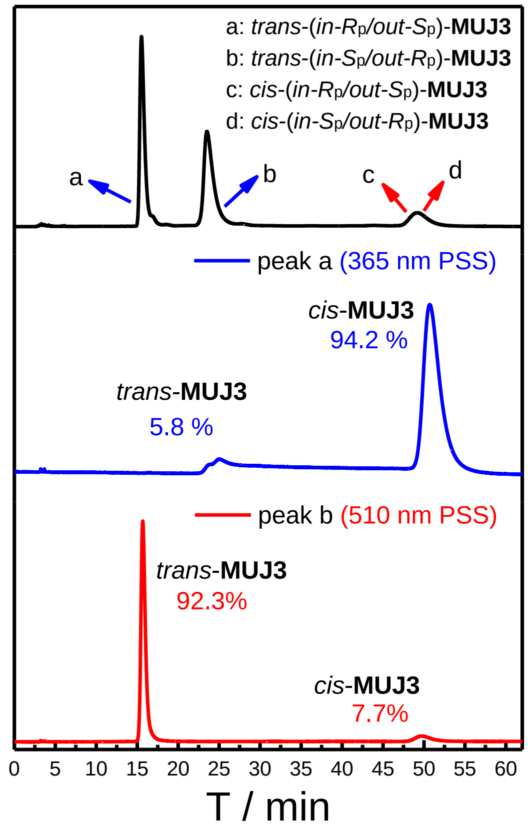

Supplementary Fig. 40 │ Chiral HPLC traces (*n*-hexane : tetrahydrofuran = 4 : 1, 320 nm) of **MUJ3** (top) and (*in*-R_p/*out*-S_p)-**MUJ3** in the PSS at 365 nm (middle) and 510 nm (bottom).

Supplementary Table 1 │ Percentage composition of *cis*- and *trans*-configurations of **MUJ1**, **MUJ2** and **MUJ3** in the PSS at 365 nm and 510 nm obtained by HPLC analysis (tetrahydrofuran : *n*-hexane = 1 : 4, 3 : 17 and 3 : 17, respectively).

Host	light wavelength	*cis*- (%)	*trans*- (%)
**MUJ1**	365 nm	97.8	2.2
	510 nm	18.5	81.5
**MUJ2**	365 nm	93.6	6.4
	510 nm	8.5	91.5
**MUJ3**	365 nm	94.2	5.8
	510 nm	7.7	92.3

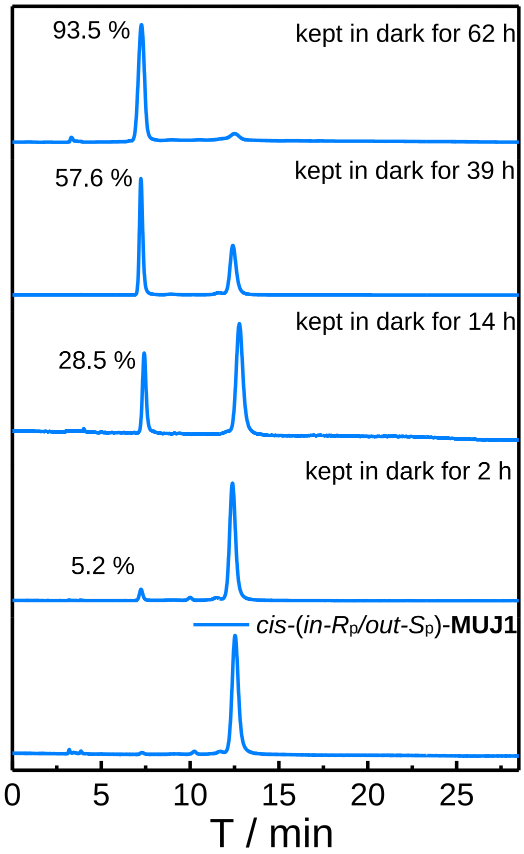

Supplementary Fig. 41 │ Chiral HPLC traces (tetrahydrofuran: *n*-hexane = 1: 4, 319 nm) of *cis*-(*in*-R_p/*out*-S_p)-**MUJ1** kept in the dark for different times at room temperature.

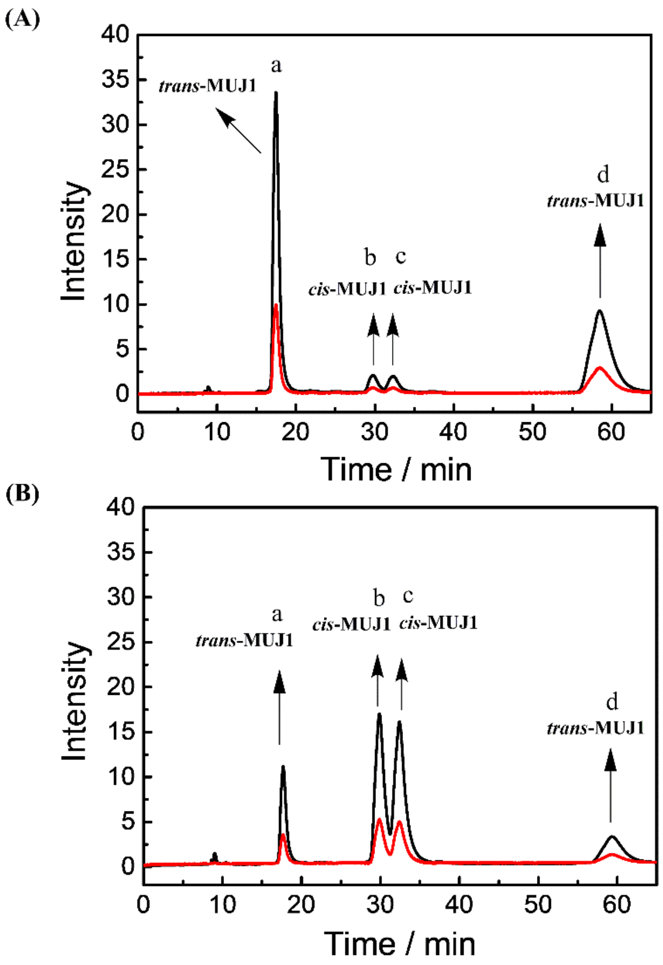

Supplementary Fig. 42 │ Chiral-phase HPLC traces of **MUJ1** (*n*-hexane : tetrahydrofuran = 4 : 1) before and after 365 nm photoirradiation (xenon grating spectrometer, 10 min), detecting at 295 nm (black) and 319 nm (red), respectively.

6. CD Spectra

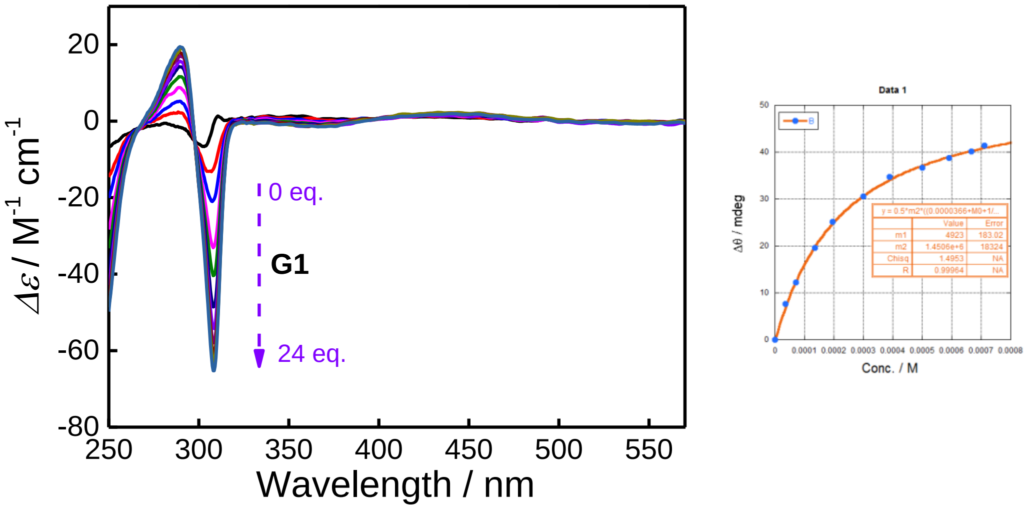

Supplementary Fig. 43 │ Left: CD spectra of *trans*-(*in*-R_p/*out*-S_p)-**MUJ1** (0.037 mM) in the presence of various amounts of **G1** (0~24 equivalents) in $CHCl_3$ at 25 °C. Right: Association constant of **MUJ1** with **G1** fitted based on CD titration.

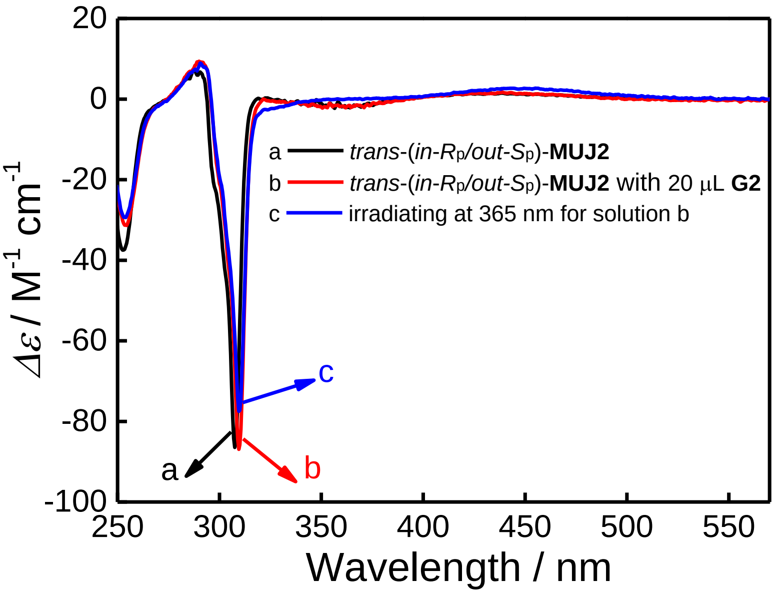

Supplementary Fig. 44 │ CD spectra (*n*-hexane : tetrahydrofuran = 17 : 3) of (a) *trans*-(*in*-R_p/*out*-S_p)-**MUJ2** (0.14 mM) (b) with excessive 1,4-dicyanobutane (**G2**) and (c) irradiated at 365 nm for 7 min at 25 °C.

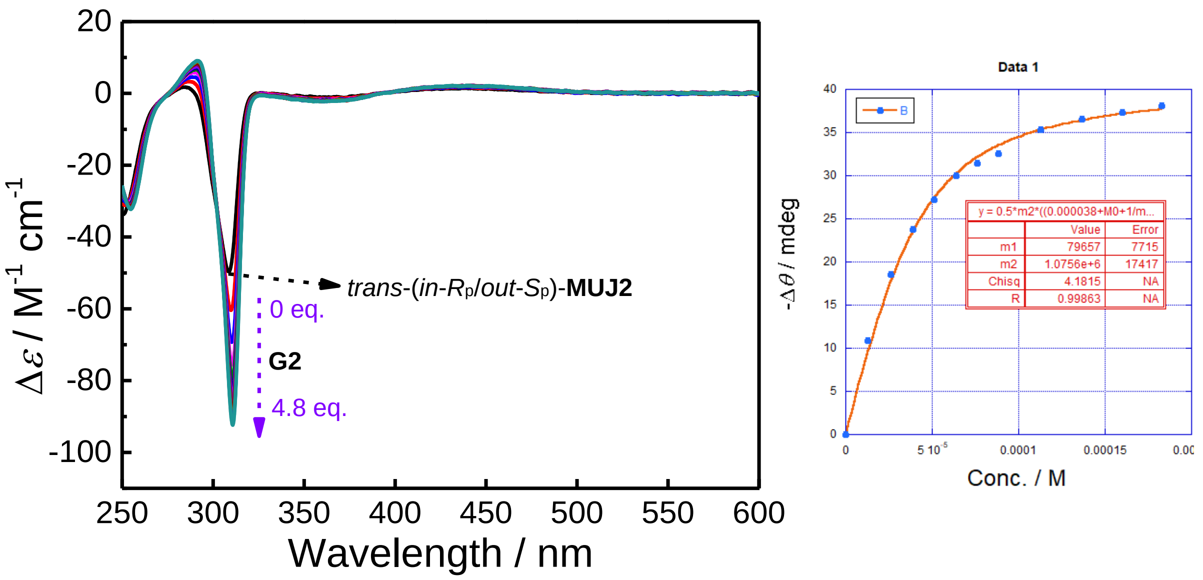

Supplementary Fig. 45 | Left: CD spectra of *trans*-(*in*-R_p/*out*-S_p)-**MUJ2** (0.038 mM) in the presence of various amounts of **G2** (0~4.8 equivalents) in $CHCl_3$ at 25 °C. Right: Association constant of **MUJ2** with **G2** fitted based on CD titration.

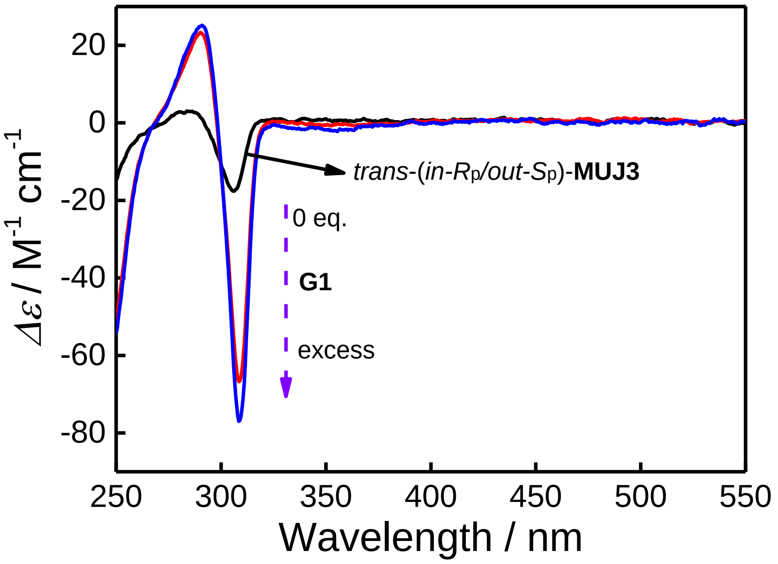

Supplementary Fig. 46 | CD spectra of *trans*-(*in*-R_p/*out*-S_p)-**MUJ3** (0.021 mM) with various amounts of **G1** in $CHCl_3$ at 25 °C.

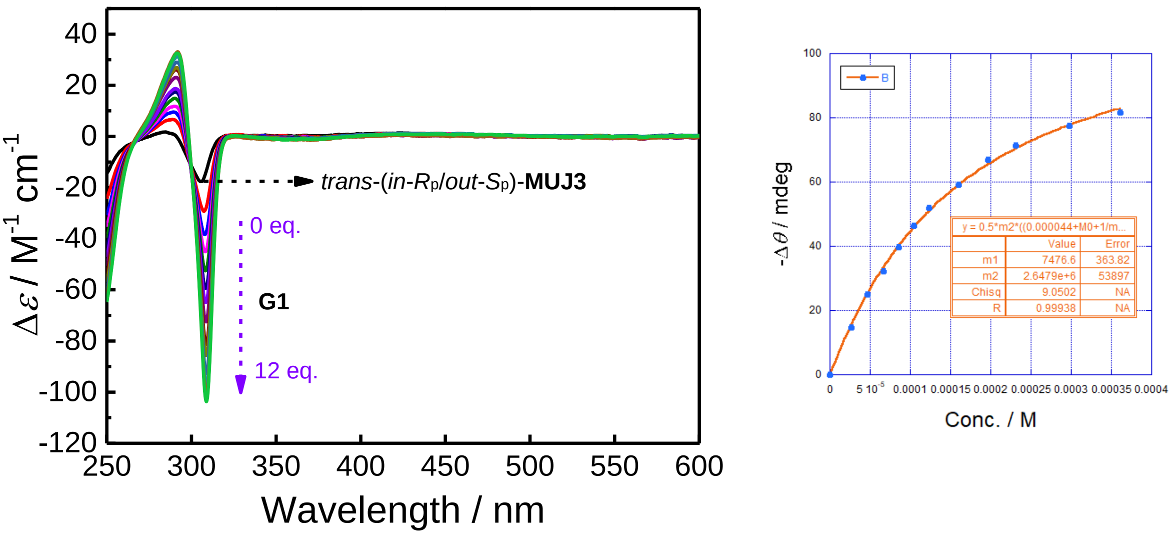

Supplementary Fig. 47 │ Left: CD spectra of *trans*-(*in*-R_p/*out*-S_p)-**MUJ3** (0.044 mM) in the presence of various amounts of **G2** (0~12.1 equivalents) in $CHCl_3$ at 25 °C. Right: Association constant of **MUJ3** with **G1** fitted based on CD titration.

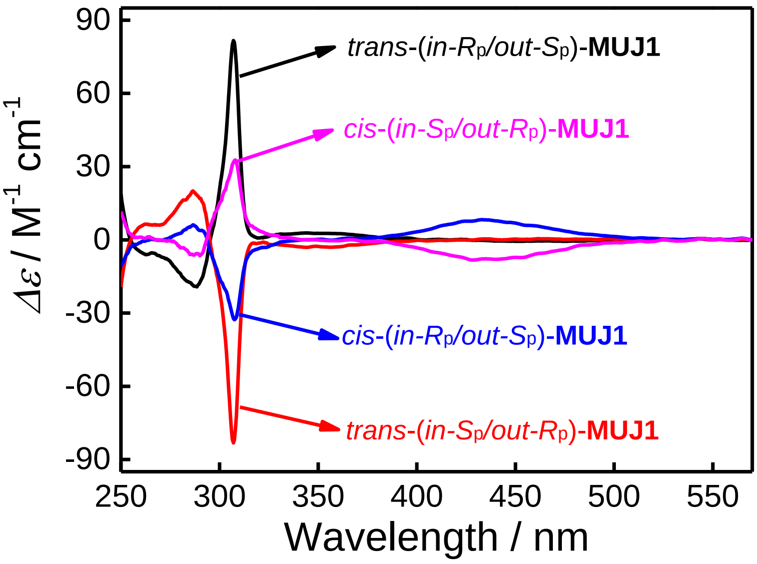

Supplementary Fig. 48 │ CD spectra of *trans*-(*in*-R_p/*out*-S_p)-**MUJ1** (black), *cis*-(*in*-R_p/*out*-S_p)-**MUJ1** (blue), *trans*-(*in*-S_p/*out*-R_p)-**MUJ1** (red) and *trans*-(*in*-S_p/*out*-R_p)-**MUJ1** (magenta) in tetrahydrofuran/*n*-hexane = 1 : 4 at 25 °C.

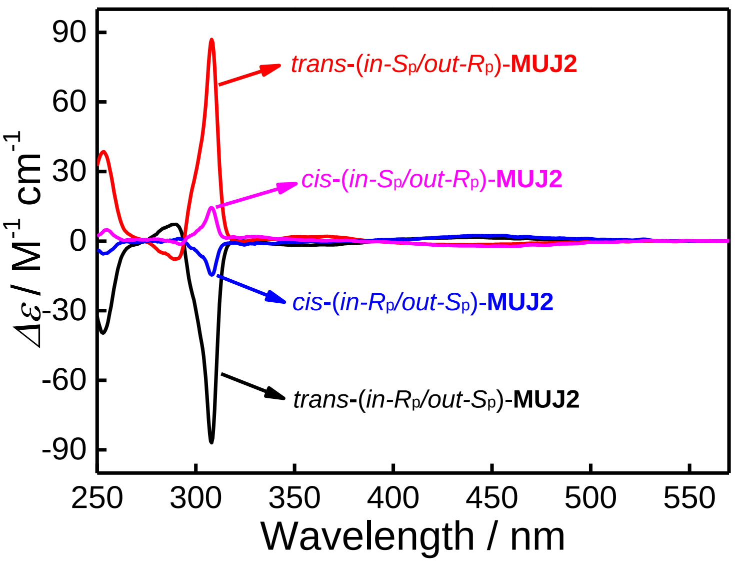

Supplementary Fig. 49 │ CD spectra of *trans*-(*in*-R_p/*out*-S_p)-**MUJ2** (black), *cis*-(*in*-R_p/*out*-S_p)-**MUJ2** (blue), *trans*-(*in*-S_p/*out*-R_p)-**MUJ2** (red) and *cis*-(*in*-S_p/*out*-R_p)-**MUJ2** (magenta) in tetrahydrofuran/*n*-hexane = 3 : 17 at 25 °C.

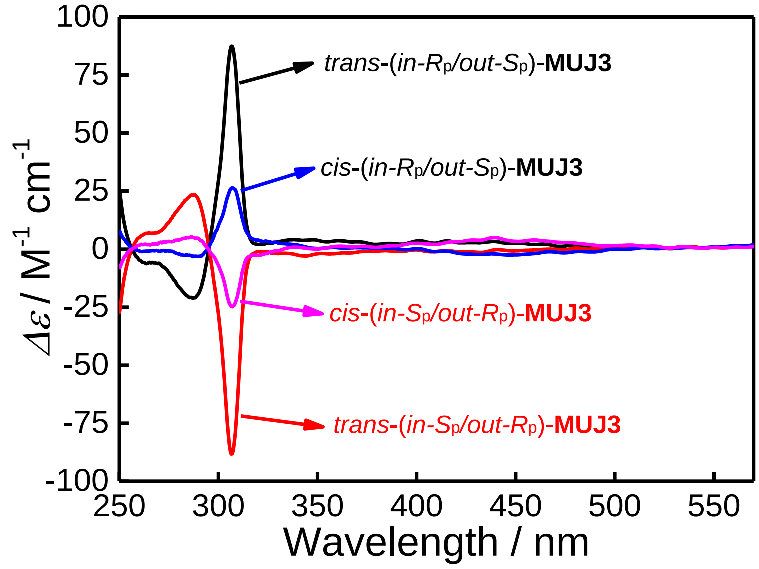

Supplementary Fig. 50 │ CD spectra of *trans*-(*in*-R_p/*out*-S_p)-**MUJ3** (black), *cis*-(*in*-R_p/*out*-S_p)-**MUJ3** (blue), *trans*-(*in*-S_p/*out*-R_p)-**MUJ3** (red) and *cis*-(*in*-S_p/*out*-R_p)-**MUJ3** (magenta) in tetrahydrofuran/*n*-hexane = 1 : 4 at 25 °C.

7. Solvent Effects on MUJs

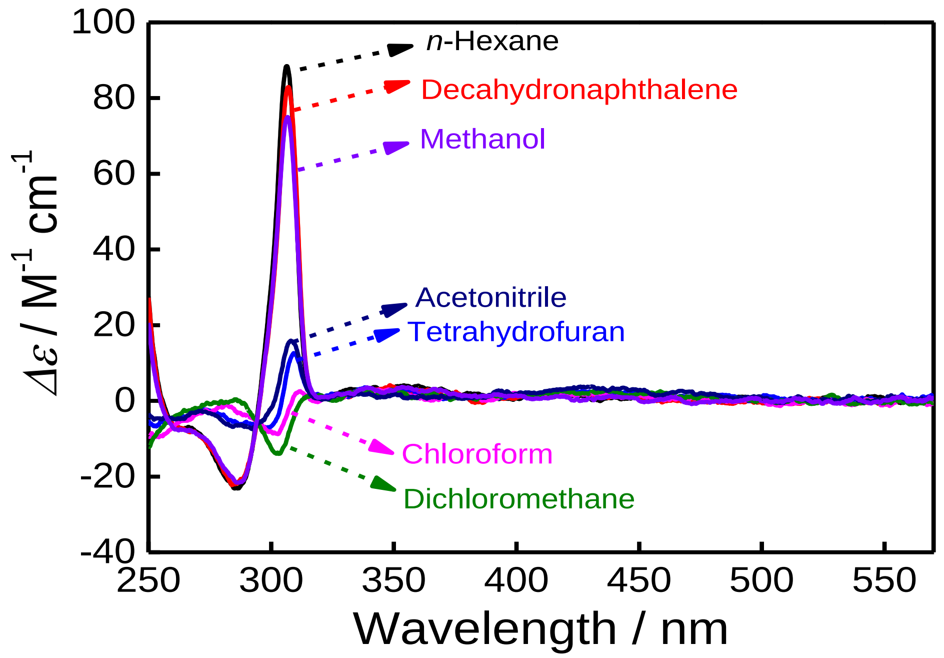

Supplementary Fig. 51 │ CD spectra of *trans*-(*in*-R_p/*out*-S_p)-**MUJ1** (0.03 mM) in various solvents at 25 °C.

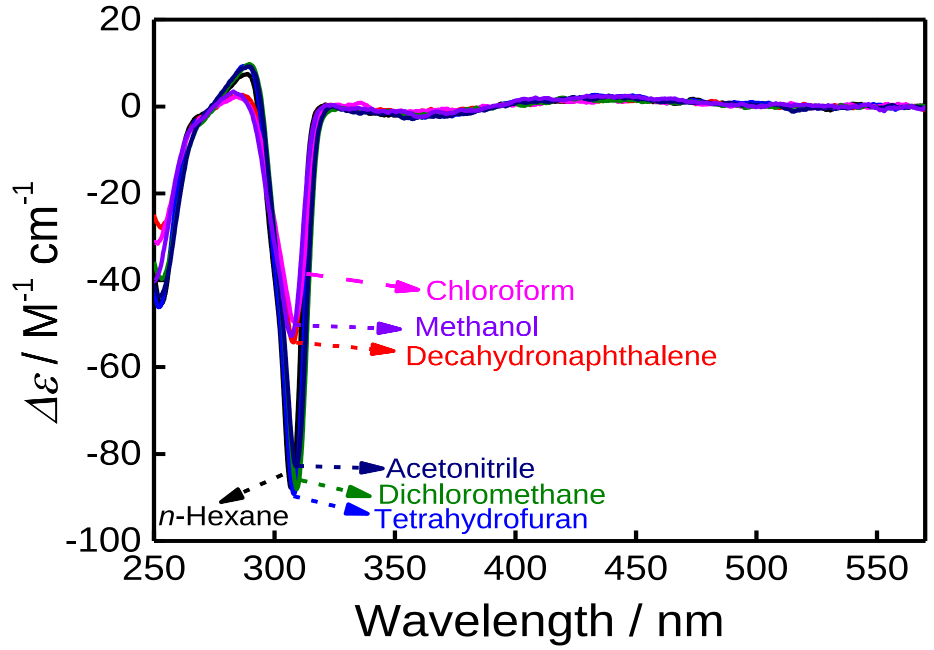

Supplementary Fig. 52 │ CD spectra of *trans*-(*in*-R_p/*out*-S_p)-**MUJ2** (0.046 mM) in various solvents at 25 °C.

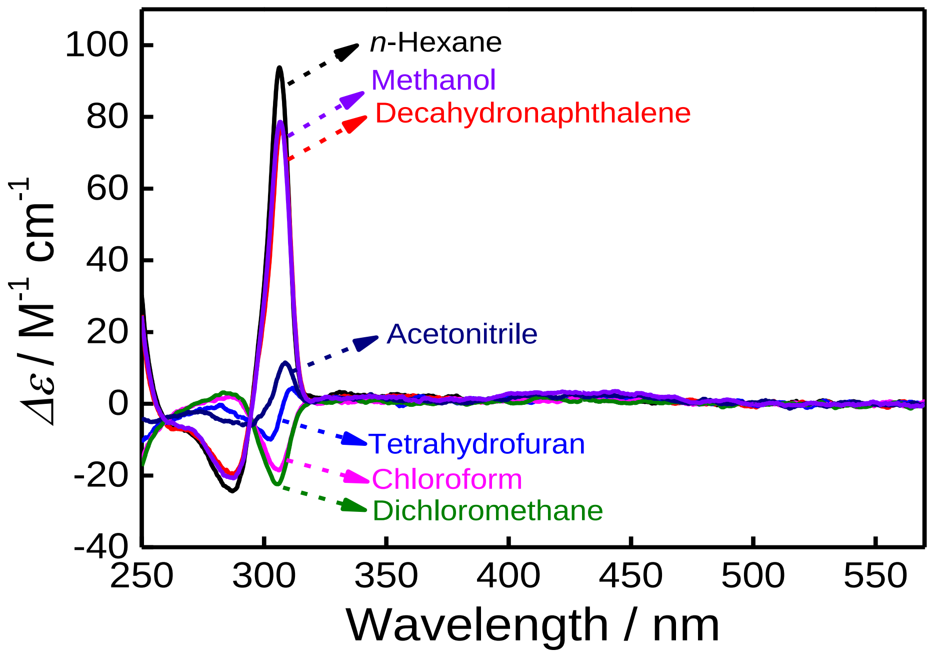

Supplementary Fig. 53 │ CD spectra of *trans*-(*in*-R_p/*out*-S_p)-**MUJ3** (0.03 mM) in various solvents at 25 °C.

8. Light-Driven Chirality Switching

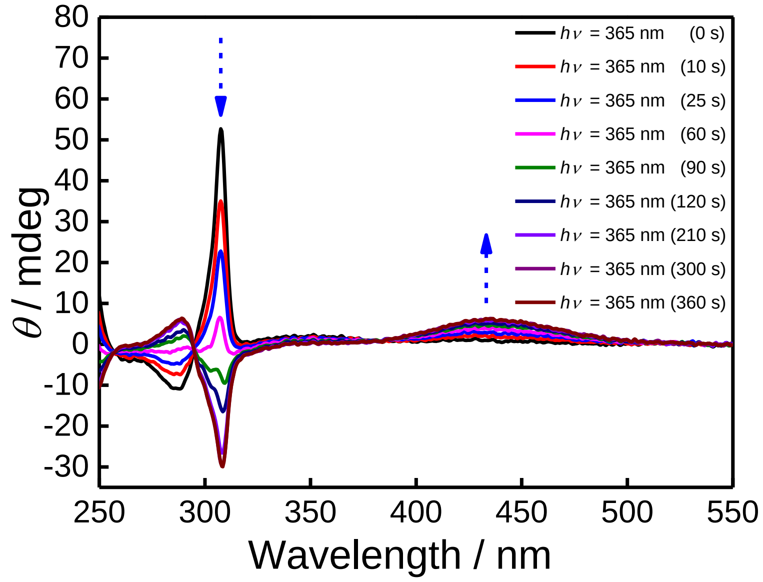

Supplementary Fig. 54 │ CD spectral changes (decahydronaphthalene, 25 ℃) of *trans*-(*in*-R_p/*out*-S_p)-**MUJ1** (0.02 mM) upon irradiation at 365 nm.

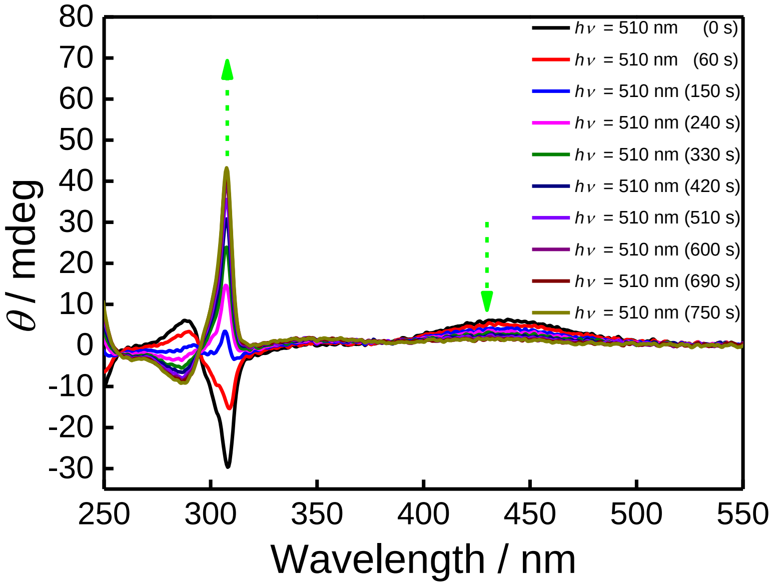

Supplementary Fig. 55 │ CD spectral changes (decahydronaphthalene, 25 ℃) of *cis*-(*in*-R_p/*out*-S_p)-**MUJ1** (0.02 mM) upon irradiation at 510 nm.

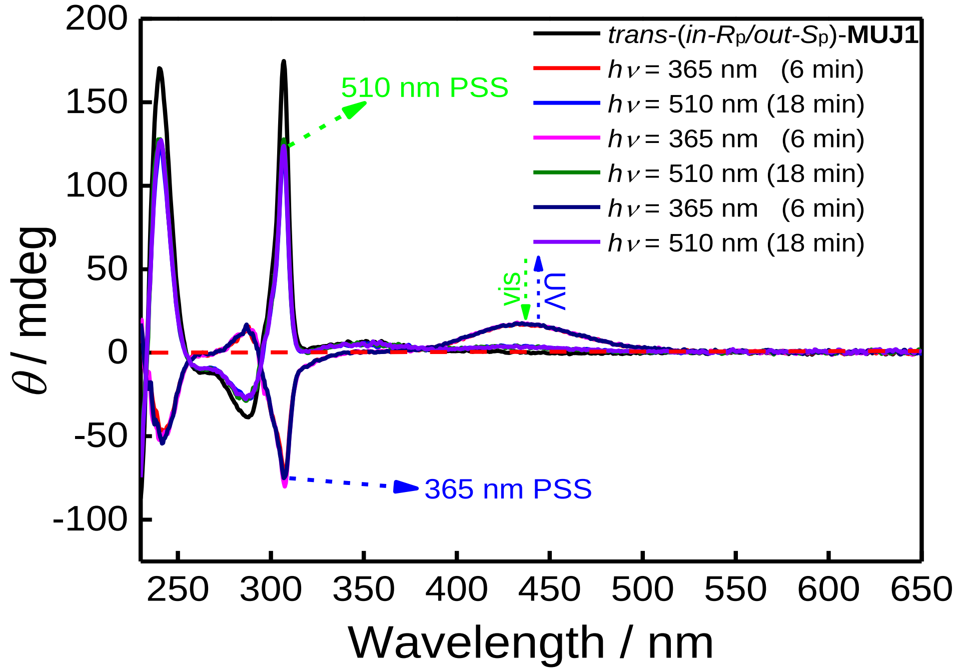

Supplementary Fig. 56 │ CD spectra (tetrahydrofuran : *n*-hexane = 1 : 4, 25 ℃) of *trans*-(*in*-R_p/*out*-S_p)-**MUJ1** (0.067 mM) and (*in*-R_p/*out*-S_p)-**MUJ1** in the PSS at 365 nm and 510 nm.

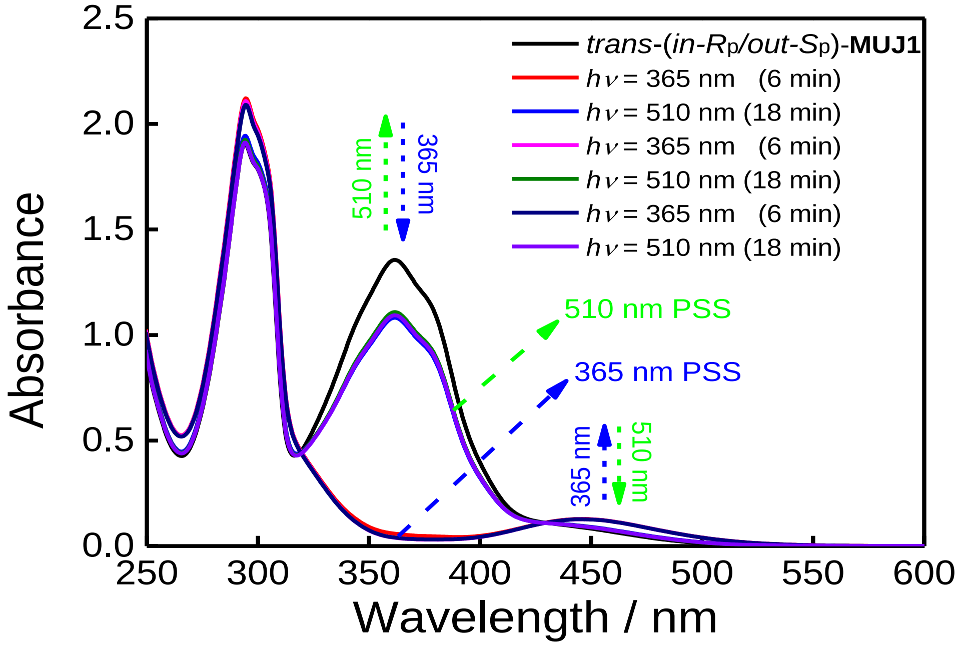

Supplementary Fig. 57 │ UV-vis spectra (tetrahydrofuran : *n*-hexane = 1 : 4) of *trans*-(*in*-R_p/*out*-S_p)-**MUJ1** (0.067 mM) and (*in*-R_p/*out*-S_p)-**MUJ1** in the PSS at 365 nm and 510 nm.

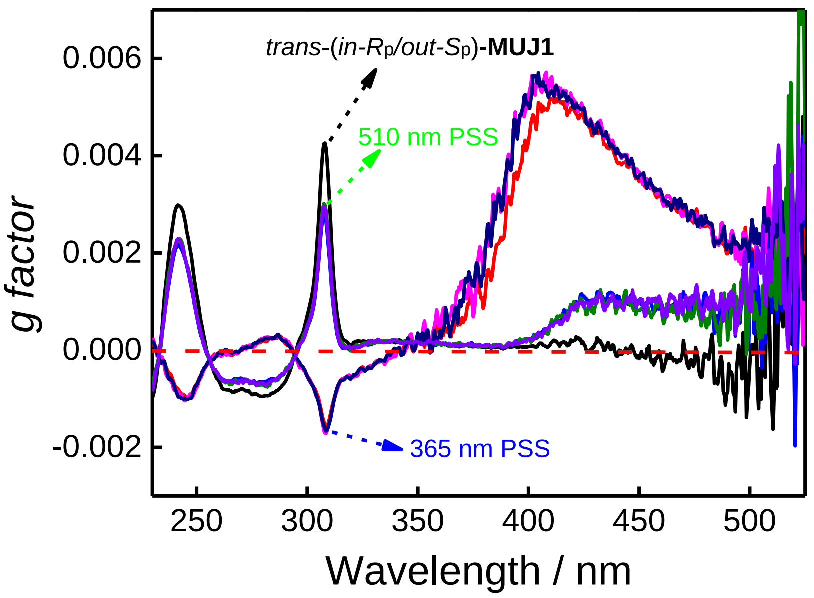

Supplementary Fig. 58 | Anisotropy factor (*g*) spectra (tetrahydrofuran : *n*-hexane = 1 : 4) of *trans*-(*in*-R_p/*out*-S_p)-**MUJ1** (0.067 mM) and (*in*-R_p/*out*-S_p)-**MUJ1** in the PSS at 365 nm and 510 nm.

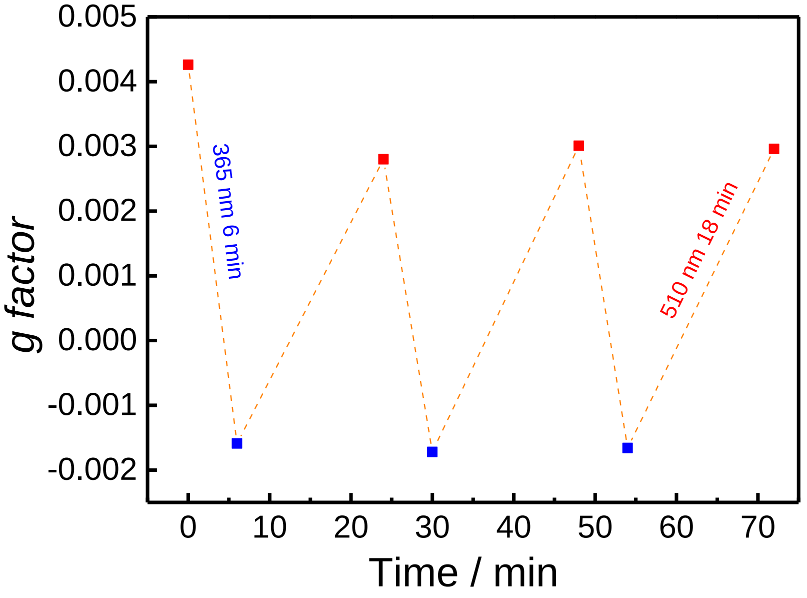

Supplementary Fig. 59 | Extremum changes (300 - 320 nm) of the anisotropy factor (*g*) (tetrahydrofuran : *n*-hexane = 1 : 4) of (*in*-R_p/*out*-S_p)-**MUJ1** (0.067 mM) in the PSS at 365 nm and 510 nm.

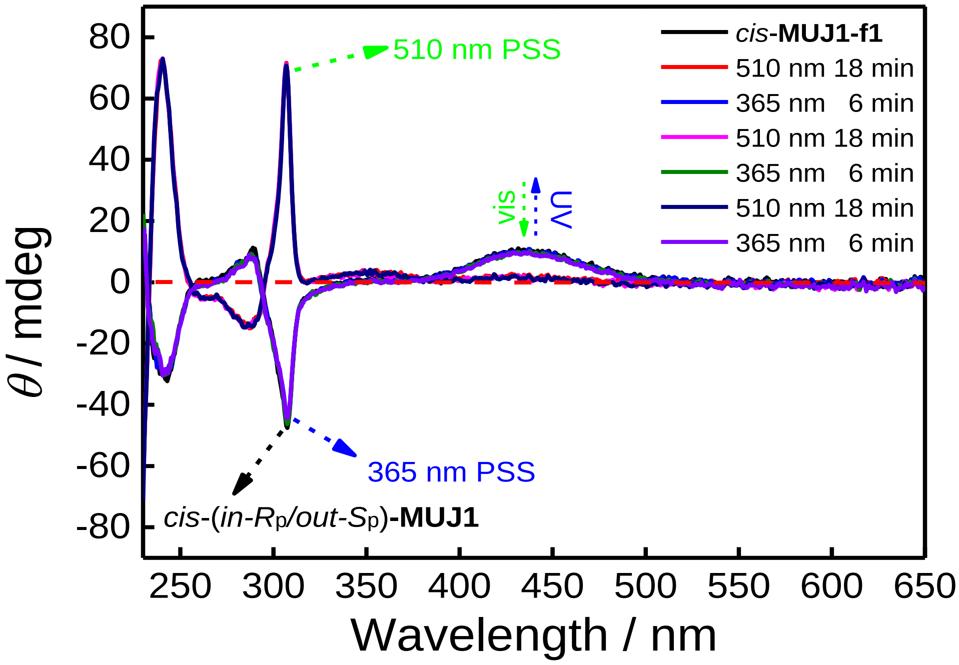

Supplementary Fig. 60 │ CD spectra (tetrahydrofuran : *n*-hexane = 1 : 4) of *cis*-(*in*-R_p/*out*-S_p)-**MUJ1** (0.045 mM) and (*in*-R_p/*out*-S_p)-**MUJ1** in the PSS at 365 nm and 510 nm.

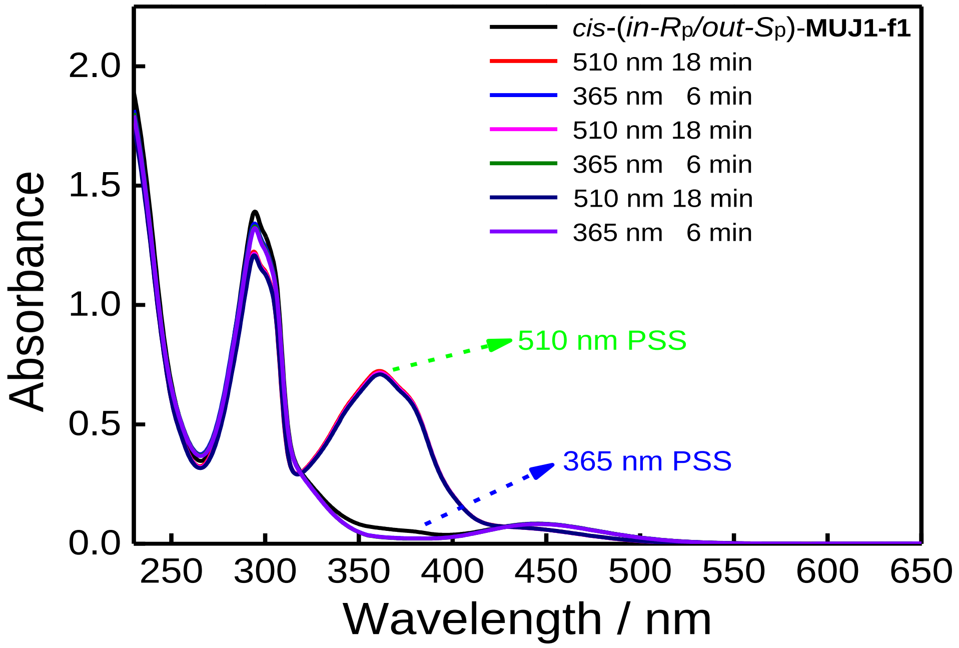

Supplementary Fig. 61 │ UV-vis spectra (tetrahydrofuran : *n*-hexane = 1 : 4) of *cis*-(*in*-R_p/*out*-S_p)-**MUJ1** (0.045 mM) and (*in*-R_p/*out*-S_p)-**MUJ1** in the PSS at 365 nm and 510 nm.

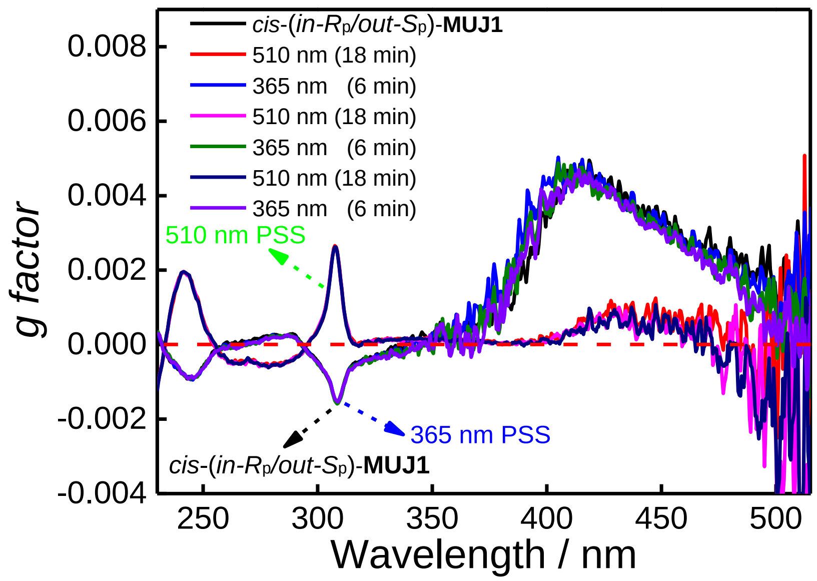

Supplementary Fig. 62 | Anisotropy factor (*g*) spectra (tetrahydrofuran : *n*-hexane = 1 : 4) of *cis*-(*in*-R_p/*out*-S_p)-**MUJ1** (0.045 mM) and (*in*-R_p/*out*-S_p)-**MUJ1** in the PSS at 365 nm and 510 nm.

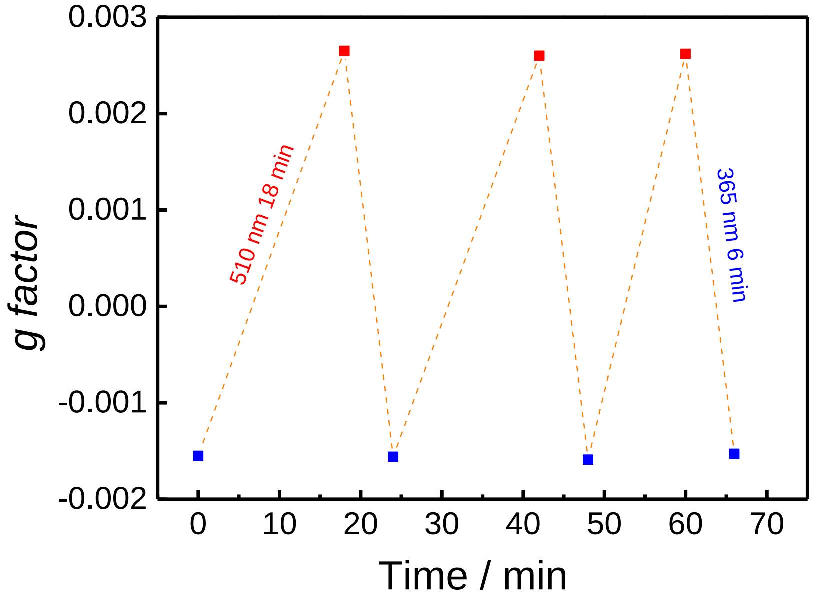

Supplementary Fig. 63 | Extremum changes (300-320 nm) of the anisotropy factor (*g*) (tetrahydrofuran : *n*-hexane = 1 : 4) of (*in*-R_p/*out*-S_p)-**MUJ1** (0.045 mM) in the PSS at 365 nm and 510 nm.

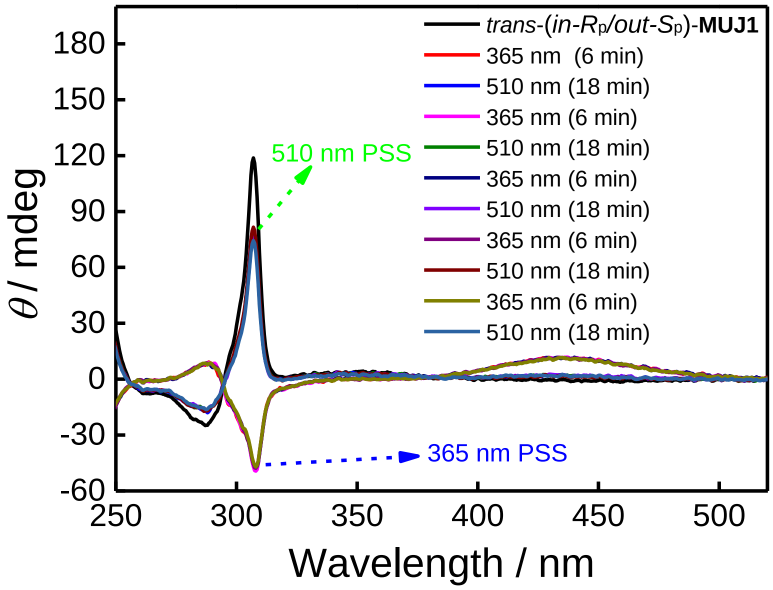

Supplementary Fig. 64 │ CD spectra of *trans*-(*in*-R_p/*out*-S_p)-**MUJ1** (0.041 mM, *n*-hexane) and (*in*-R_p/*out*-S_p)-**MUJ1** in the PSS at 365 nm and 510 nm.

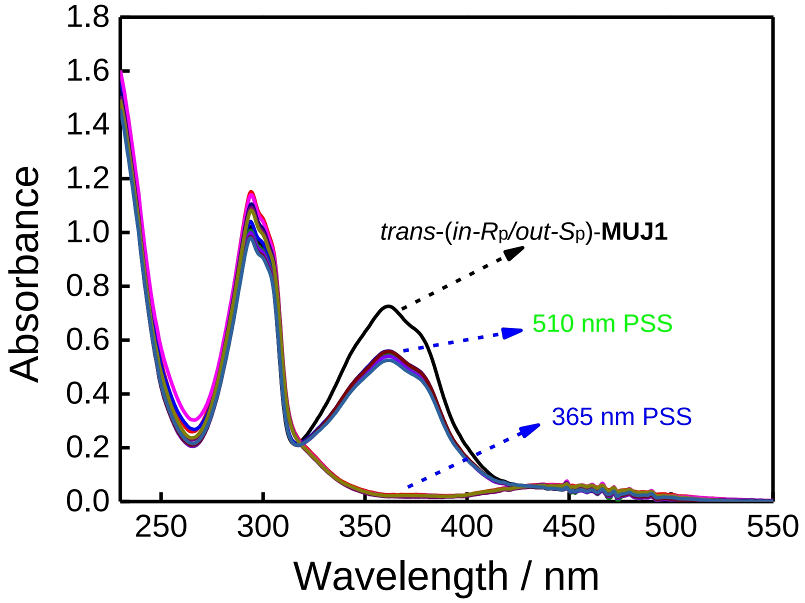

Supplementary Fig. 65 │ UV-vis spectra of *trans*-(*in*-R_p/*out*-S_p)-**MUJ1** (0.041 mM, *n*-hexane) and (*in*-R_p/*out*-S_p)-**MUJ1** in the PSS at 365 nm and 510 nm.

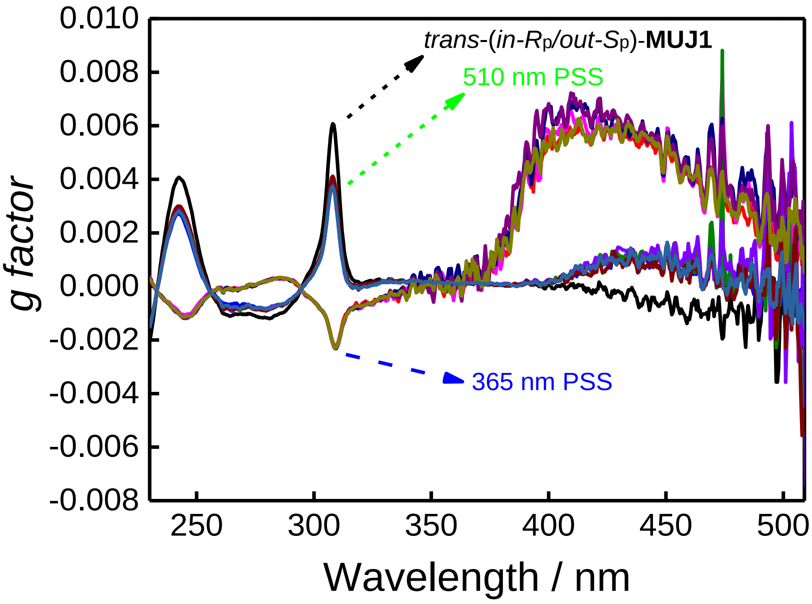

Supplementary Fig. 66 │ Anisotropy factor (*g*) spectra of (*in*-R_p/*out*-S_p)-**MUJ1** (0.041 mM, *n*-hexane) and (*in*-R_p/*out*-S_p)-**MUJ1** in the PSS at 365 nm and 510 nm.

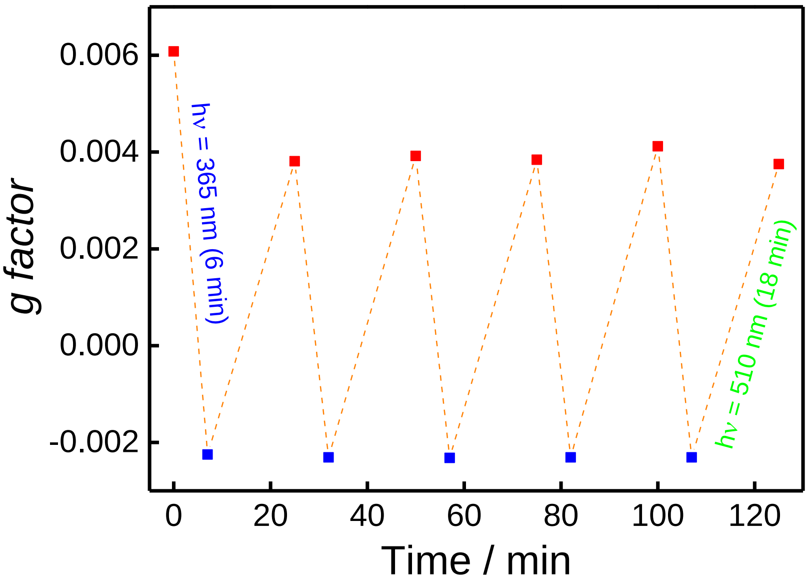

Supplementary Fig. 67 │ Extremum changes (300 - 320 nm) of the anisotropy factor (*g*) of (*in*-R_p/*out*-S_p)-**MUJ1** (0.041 mM, *n*-hexane) in the PSS at 365 nm and 510 nm.

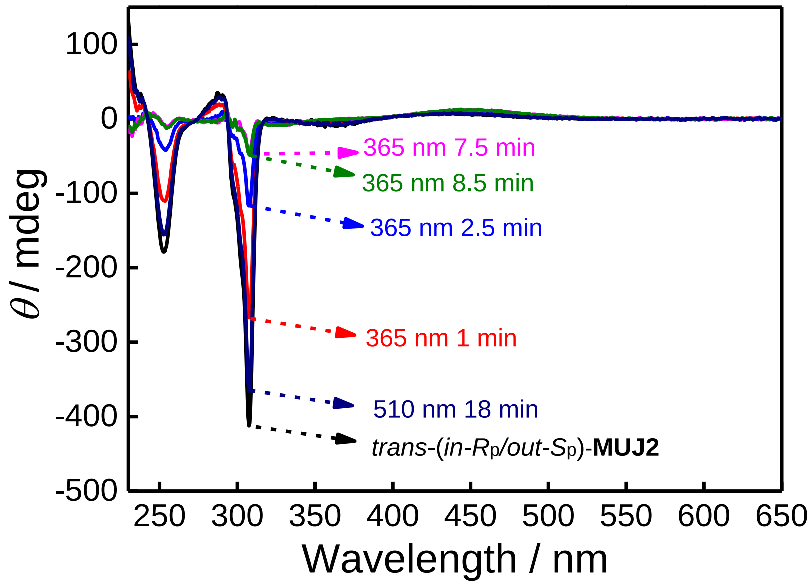

Supplementary Fig. 68 │ CD spectra of *trans*-(*in*-R_p/*out*-S_p)-**MUJ2** (0.14 mM, tetrahydrofuran/*n*-hexane = 3 : 17) under irradiation at 365 nm and 510 nm.

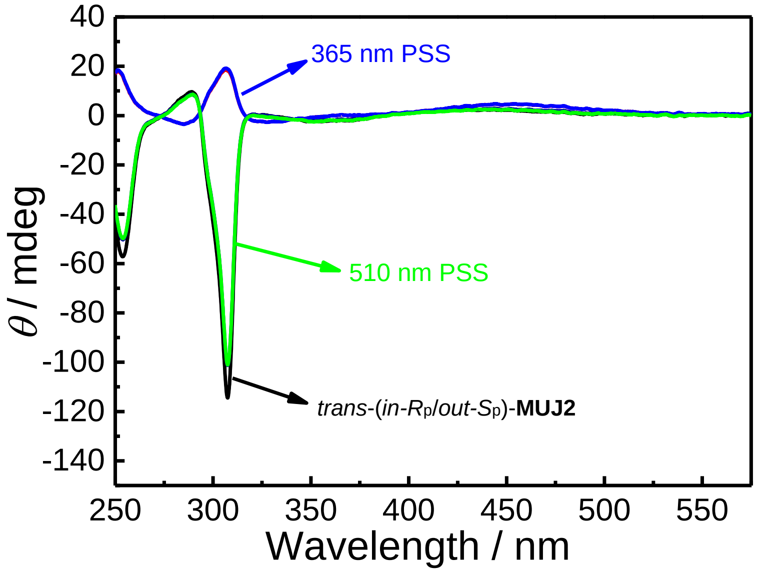

Supplementary Fig. 69 │ CD spectra of *trans*-(*in*-R_p/*out*-S_p)-**MUJ2** (0.039 mM, *n*-hexane) and (*in*-R_p/*out*-S_p)-**MUJ2** in the PSS at 365 nm and 510 nm.

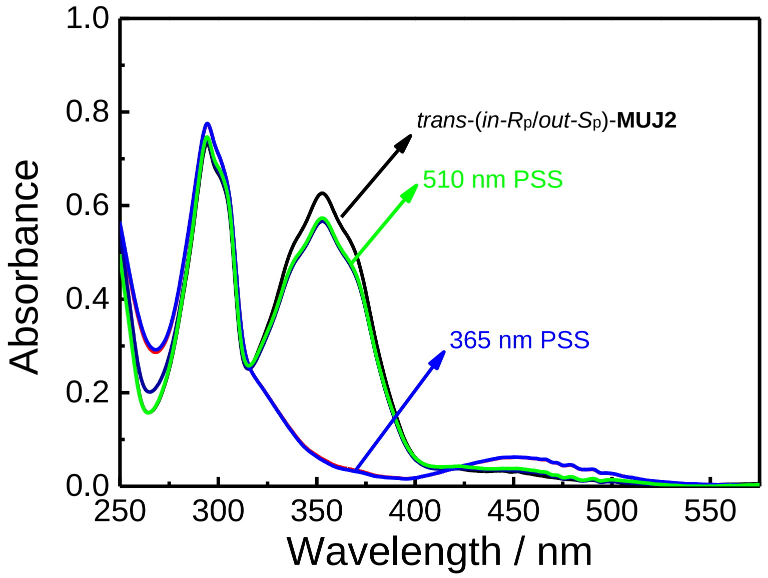

Supplementary Fig. 70 │ UV-vis spectra of *trans*-(*in*-R_p/*out*-S_p)-**MUJ2** (0.039 mM, *n*-hexane) and (*in*-R_p/*out*-S_p)-**MUJ2** in the PSS at 365 nm and 510 nm.

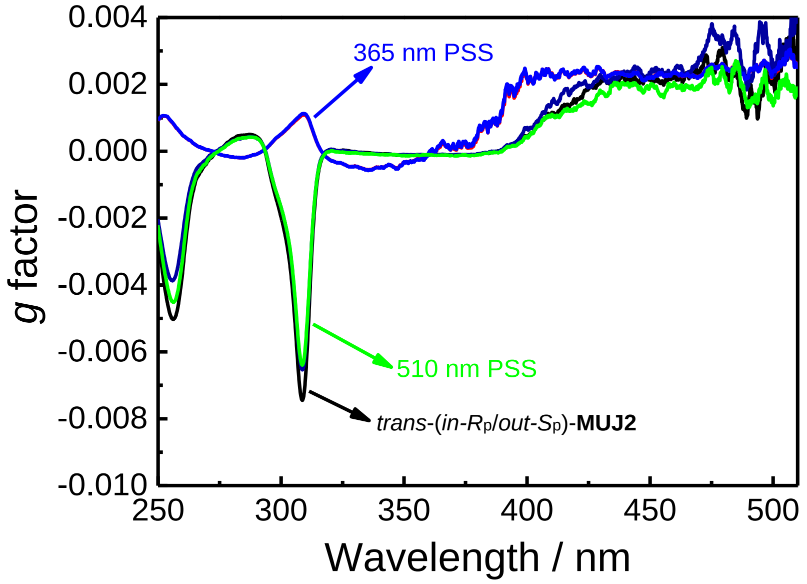

Supplementary Fig. 71 │ Anisotropy factor (*g*) spectra of *trans*-(*in*-R_p/*out*-S_p)-**MUJ2** (0.039 mM, *n*-hexane) and (*in*-R_p/*out*-S_p)-**MUJ2** in the PSS at 365 nm and 510 nm.

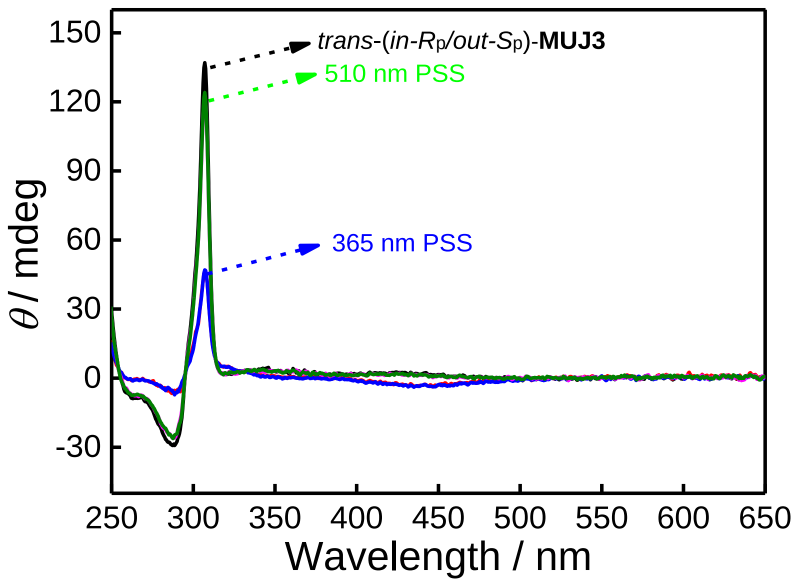

Supplementary Fig. 72 │ CD spectra of *trans*-(*in*-R_p/*out*-S_p)-**MUJ3** (0.047 mM, tetrahydrofuran/*n*-hexane = 3 : 17) under irradiation at 365 nm and 510 nm.

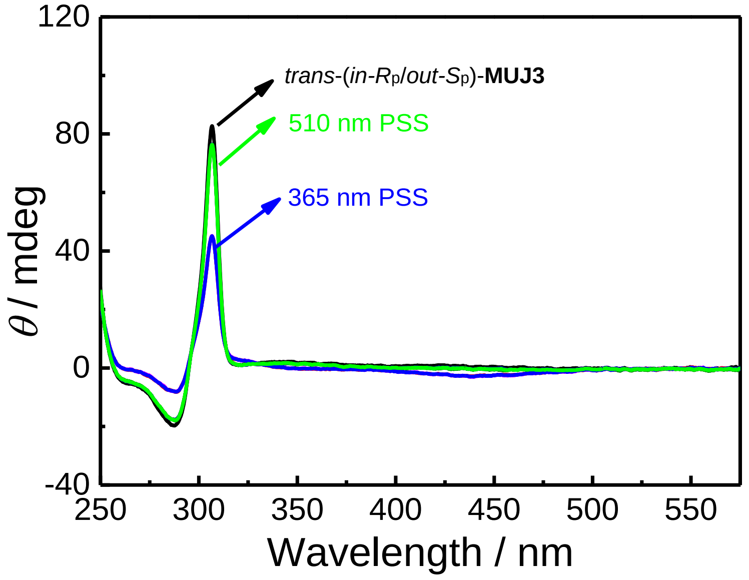

Supplementary Fig. 73 │ CD spectra of *trans*-(*in*-R_p/*out*-S_p)-**MUJ3** (0.027 mM, *n*-hexane) and (*in*-R_p/*out*-S_p)-**MUJ3** in the PSS at 365 nm and 510 nm.

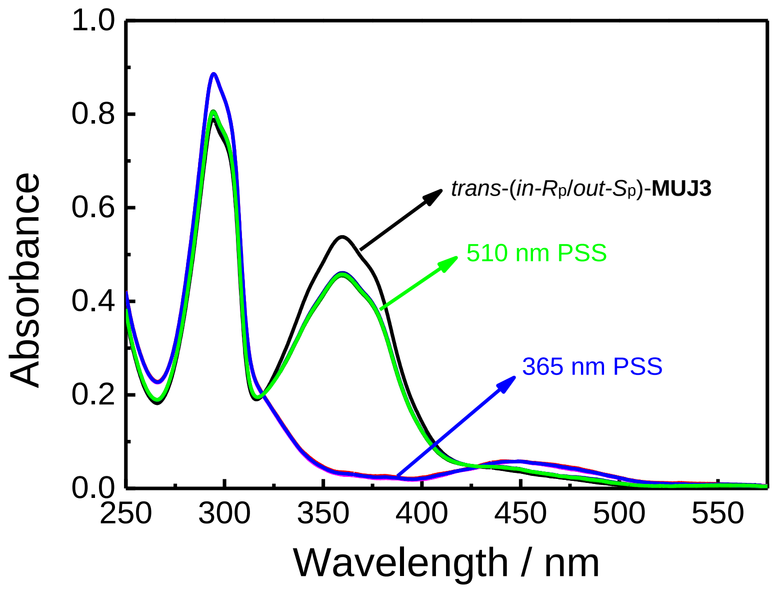

Supplementary Fig. 74 │ UV-vis spectra of *trans*-(*in*-R_p/*out*-S_p)-**MUJ3** (0.027 mM, *n*-hexane) and (*in*-R_p/*out*-S_p)-**MUJ3** in the PSS at 365 nm and 510 nm.

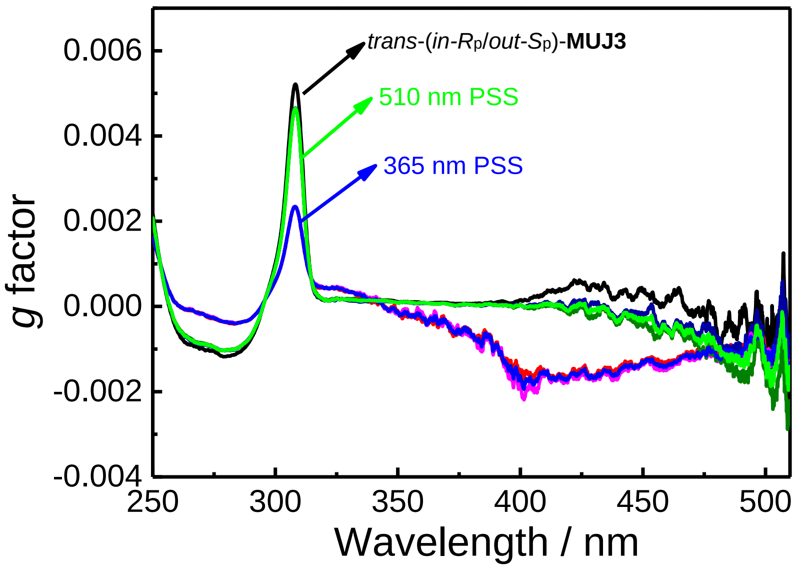

Supplementary Fig. 75 │ Anisotropy factor (*g*) spectra of *trans*-(*in*-R_p/*out*-S_p)-**MUJ3** (0.027 mM, *n*-hexane) and (*in*-R_p/*out*-S_p)-**MUJ3** in the PSS at 365 nm and 510 nm.

9. Chirality Switching in Coating Film

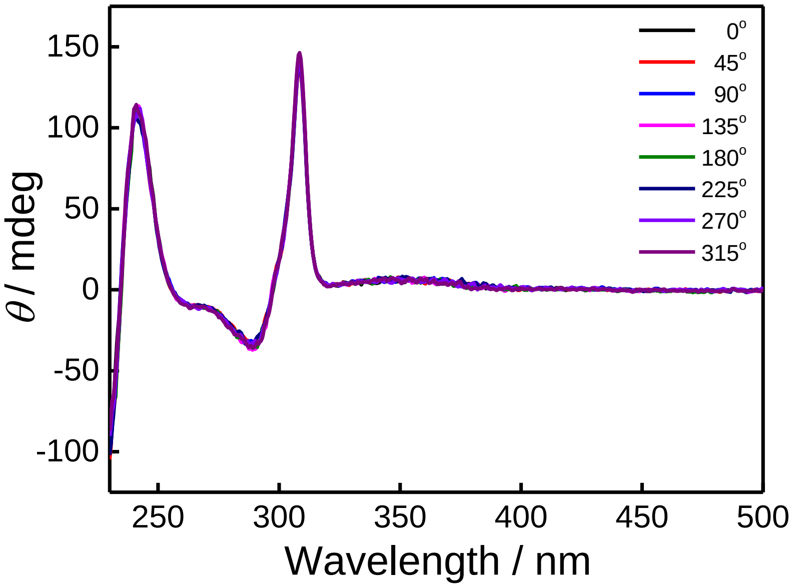

Supplementary Fig. 76 │ CD spectra of the spin-coated film of *trans*-(*in*-R_p/*out*-S_p)-**MUJ1** detected at different angles.

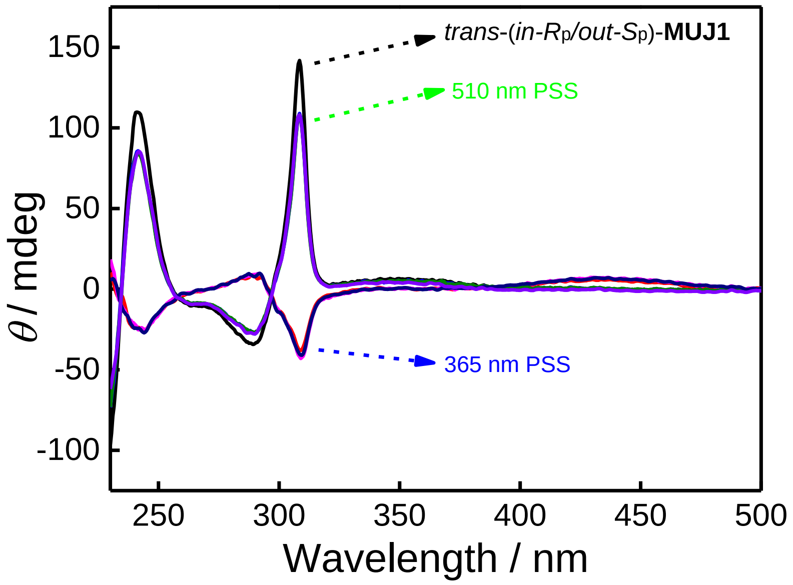

Supplementary Fig. 77 │ CD spectra of the spin-coated film of (*in*-R_p/*out*-S_p)-**MUJ1** under irradiation at 365 nm and 510 nm.

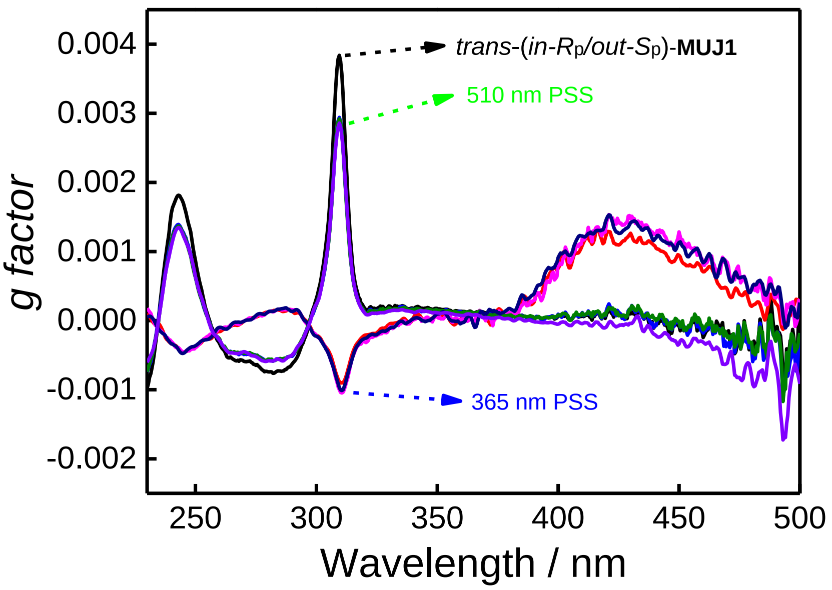

Supplementary Fig. 78 │ Anisotropy factor (*g*) spectra of the spin-coated film of (*in*-R_p/*out*-S_p)-**MUJ1** under irradiation at 365 nm and 510 nm.

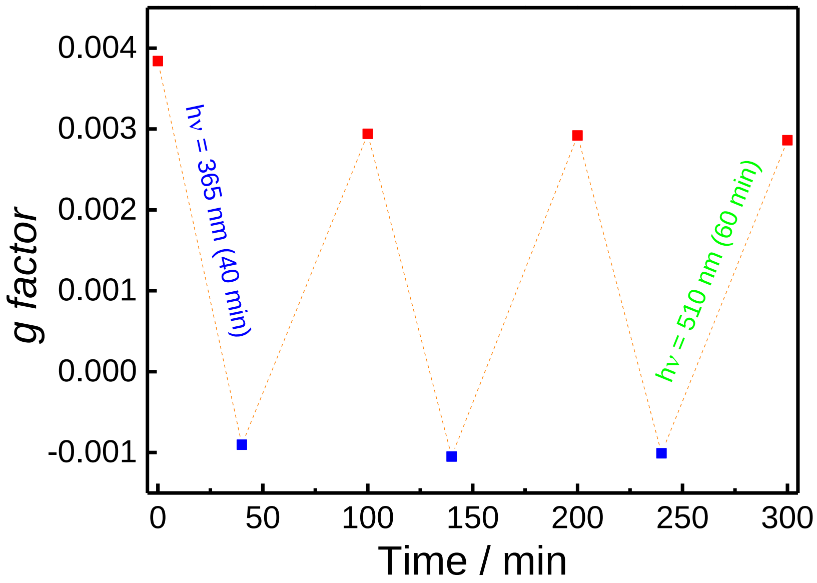

Supplementary Fig. 79 │ Extremum changes (300 - 320 nm) of the anisotropy factor (*g*) of the spin-coated film of (*in*-R_p/*out*-S_p)-**MUJ1** upon irradiation at 365 nm and 510 nm.

10. Photoresponsive ^{1}H NMR Spectra of MUJs

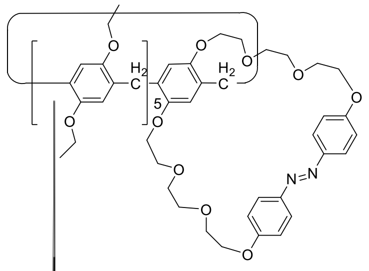

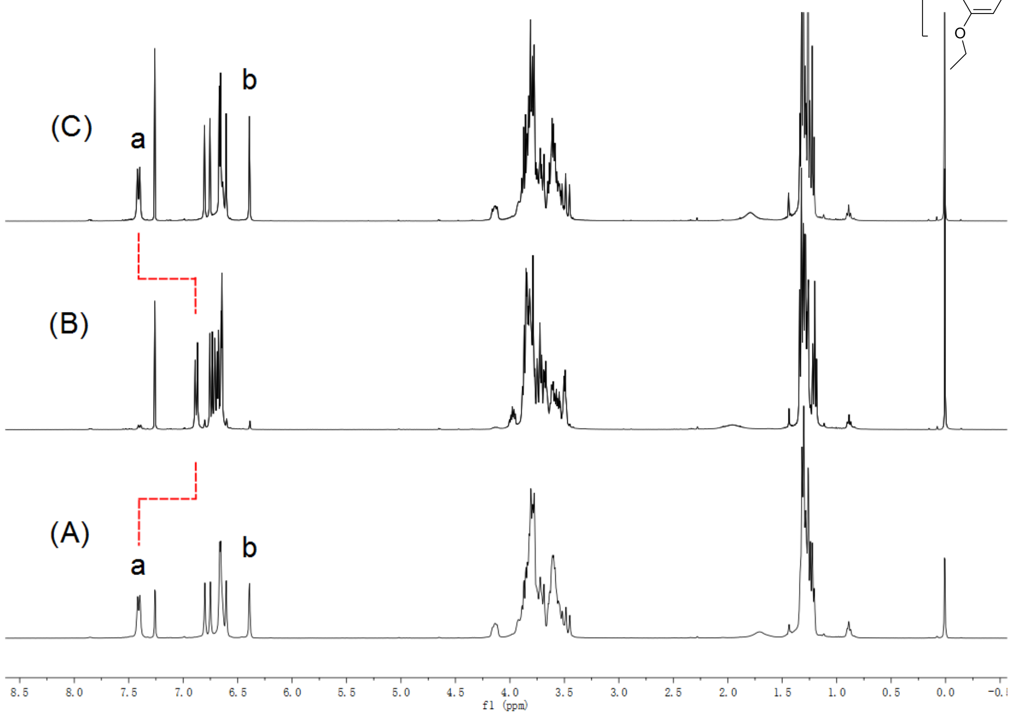

Supplementary Fig. 80 | ^{1}H NMR of **MUJ1** in $CDCl_3$ before (A) and after (B) irradiation with 365 nm LED illumination (C) and then after being kept in the dark for 1 week.

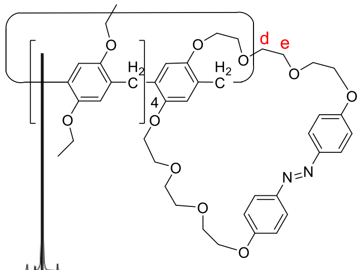

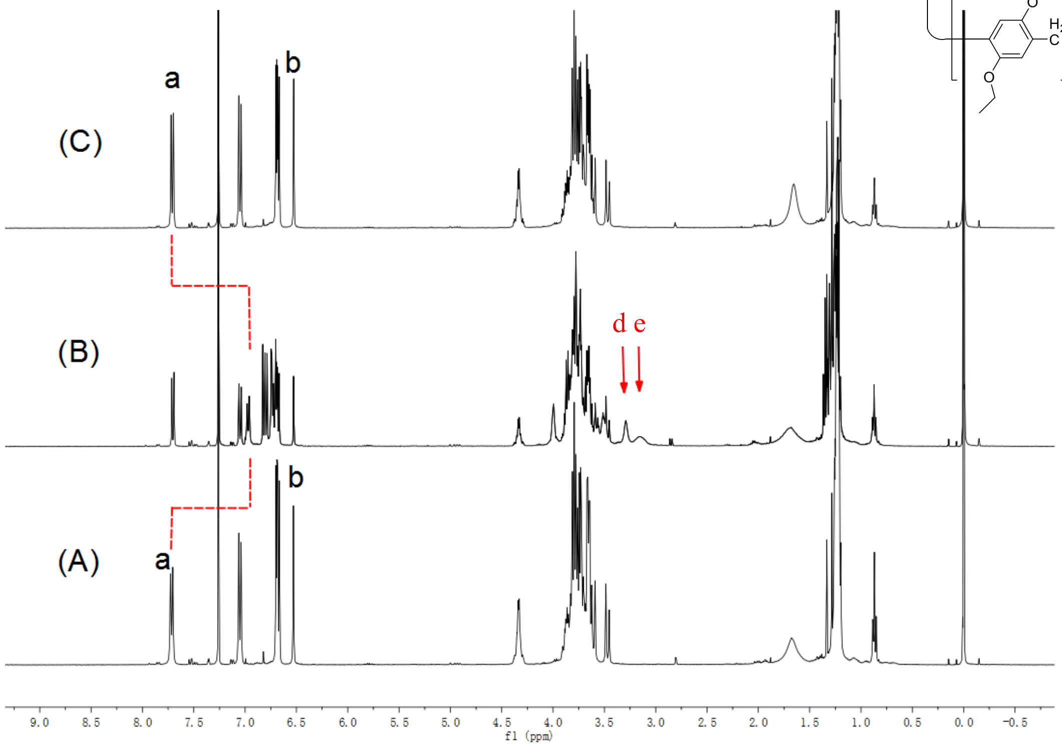

Supplementary Fig. 81 | ^{1}H NMR of **MUJ2** in $CDCl_3$ before (A) and after (B) irradiation with 365 nm LED illumination (C) and then after being kept in the dark for 1 week.

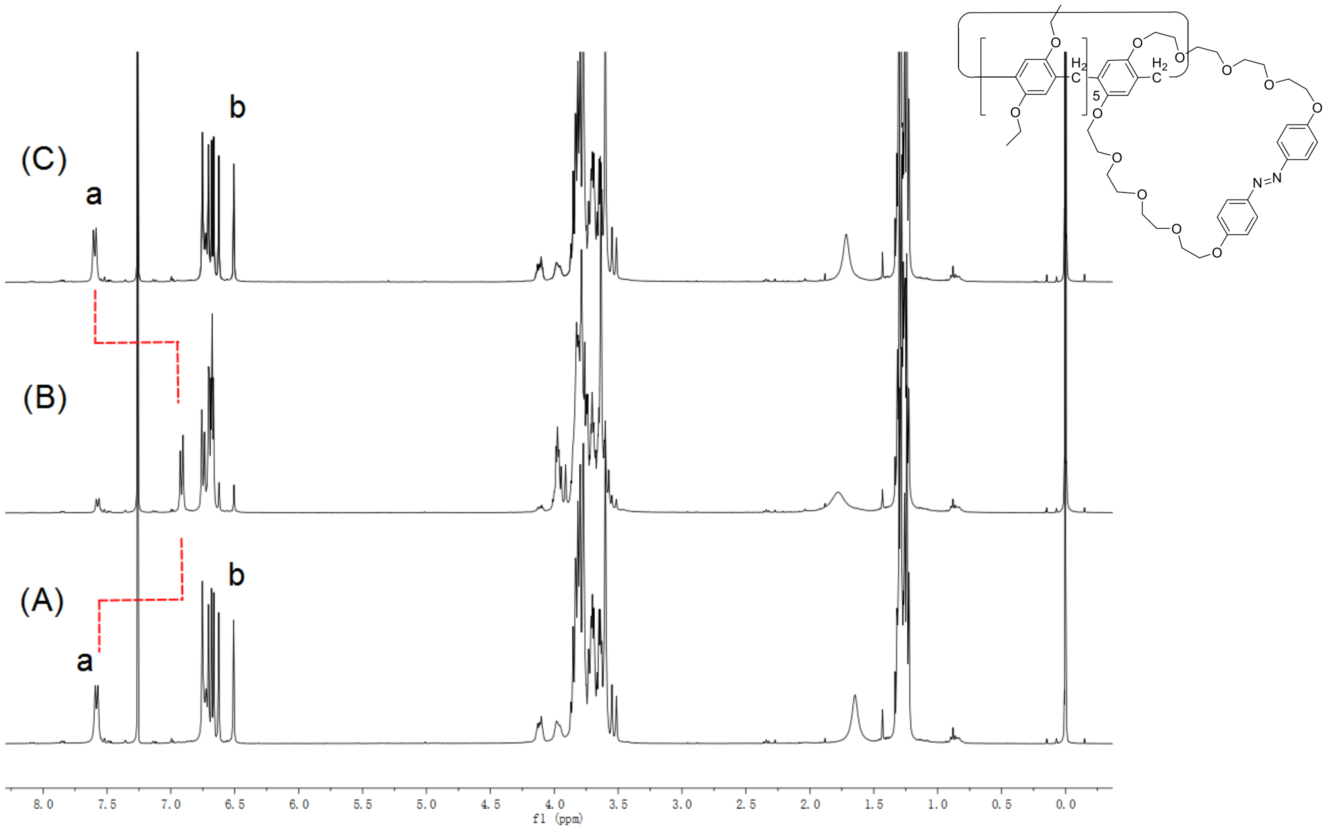

Supplementary Fig. 82 | ^{1}H NMR of **MUJ3** in $CDCl_3$ before (A) and after (B) irradiation with 365 nm LED illumination and then after (C) being kept in the dark for 1 week.

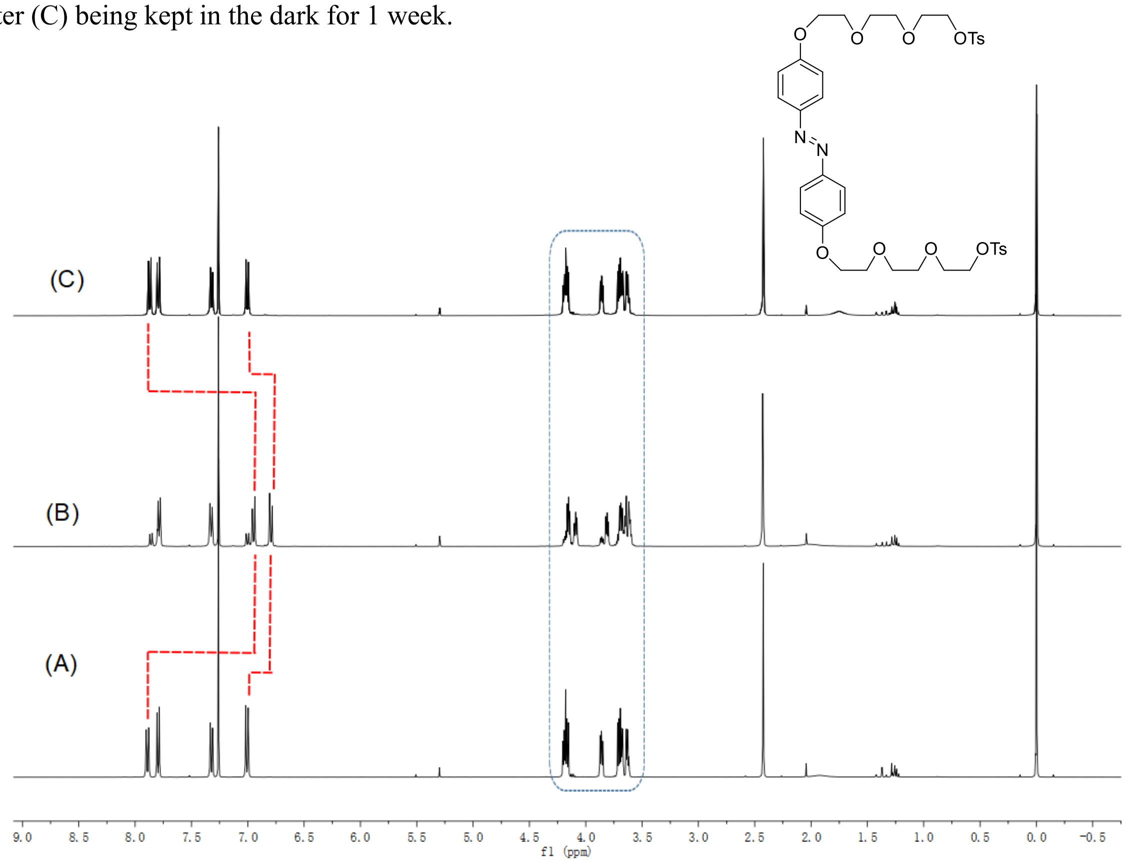

Supplementary Fig. 83 | ^{1}H NMR of **3a** in $CDCl_3$ before (A) and after (B) irradiation with 365 nm LED illumination (2.5 min) (C) and then after being kept in the dark for 1 week.

11. Molecular Model Studies of MUJ2

(a) **(b)**

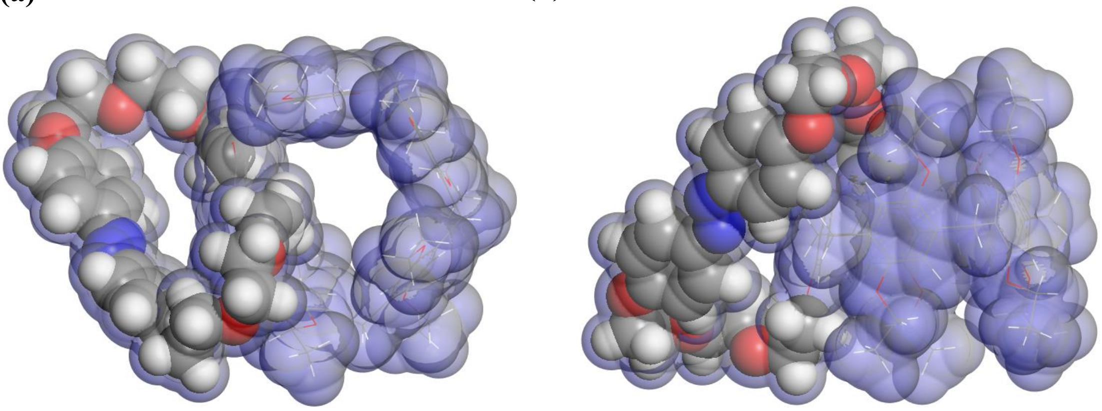

Supplementary Fig. 84 │ (a) Top and (b) front views of the *trans*-*out*-(S_p)-**MUJ2** geometry optimized by DFT calculations at the B3LYP/6-31G(d) level with Gaussian 09W.

(a) **(b)**

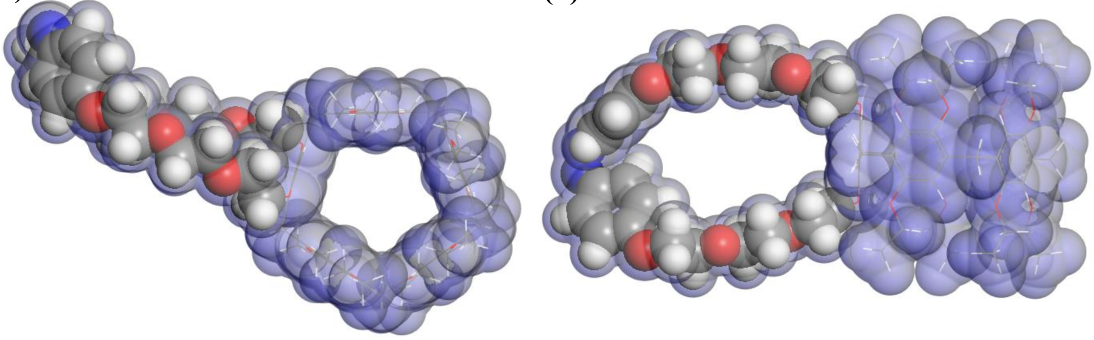

Supplementary Fig. 85 │ (a) Top and (b) front views of the *cis*-*out*-(S_p)-**MUJ2** geometry optimized by DFT calculations at the B3LYP/6-31G(d) level with Gaussian 09W.

(a) **(b)**

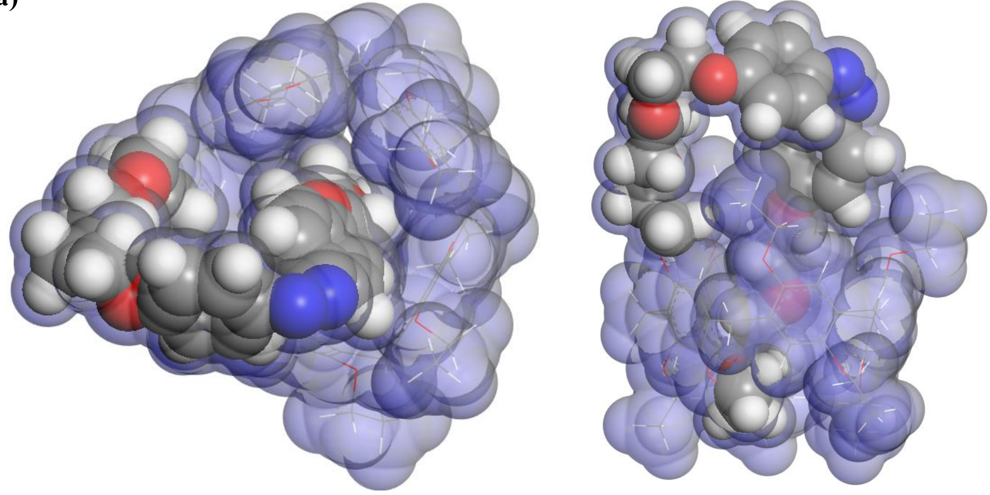

Supplementary Fig. 86 │ (a) Top and (b) front views of *cis*-*in*-(R_p)-**MUJ2** adopting a partially self-included conformation, with the geometry optimized by DFT calculations at the B3LYP/6-31G(d) level with Gaussian 09W.

12. VT CD Spectra of *trans*-(*in*-R_p/*out*-S_p)-MUJ1

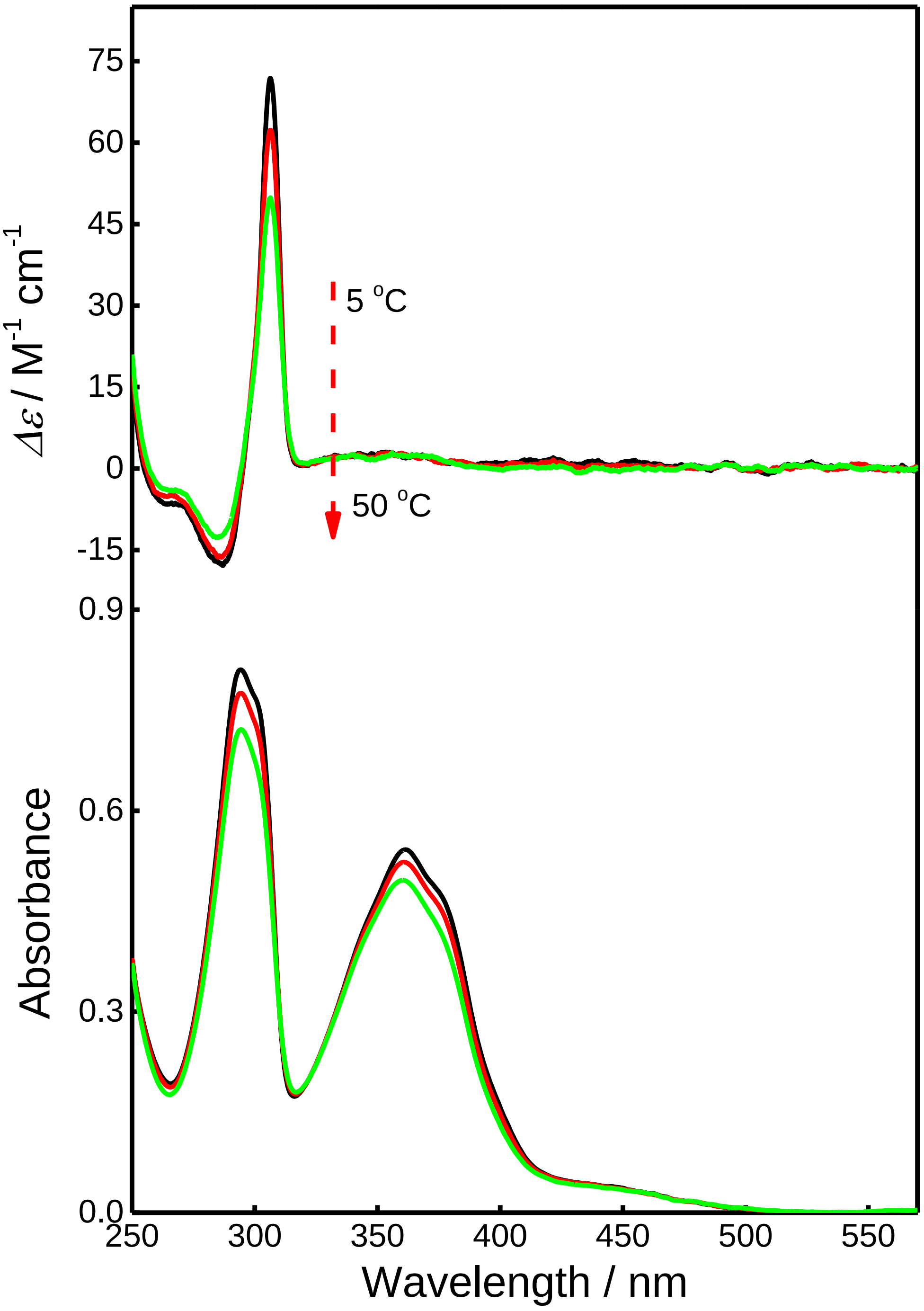

Supplementary Fig. 87 │ V-T CD spectra of *trans*-(*in*-R_p/*out*-S_p)-**MUJ1** (0.03 mM) in *n*-hexane at 5 °C (black), 25 °C (red) and 50 °C (green) respectively.

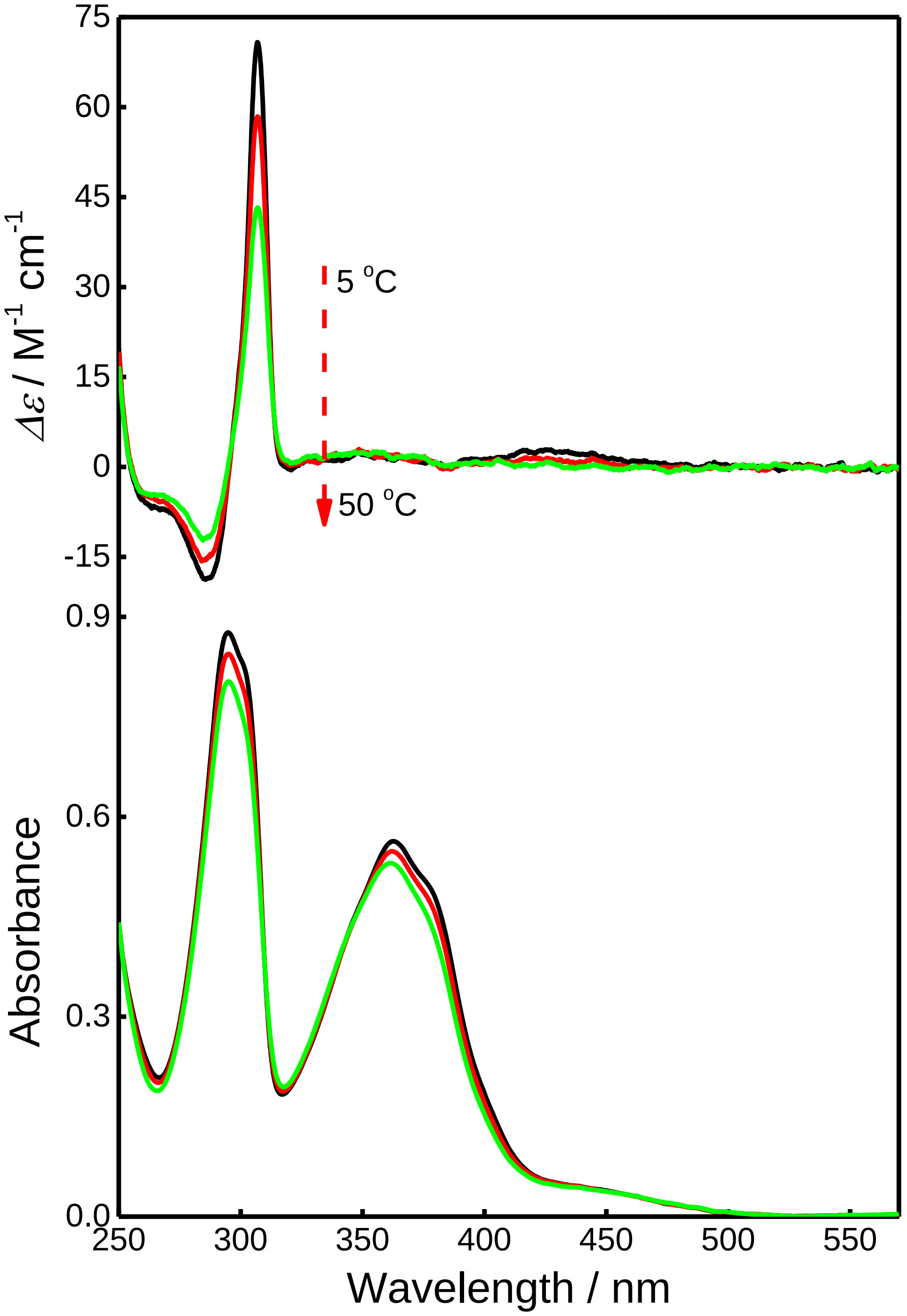

Supplementary Fig. 88 | V-T CD spectra of *trans*-(*in*-R_p/*out*-S_p)-**MUJ1** (0.03 mM) in decahydronaphthalene at 5 ºC (black), 25 ºC (red) and 50 ºC (green) respectively.

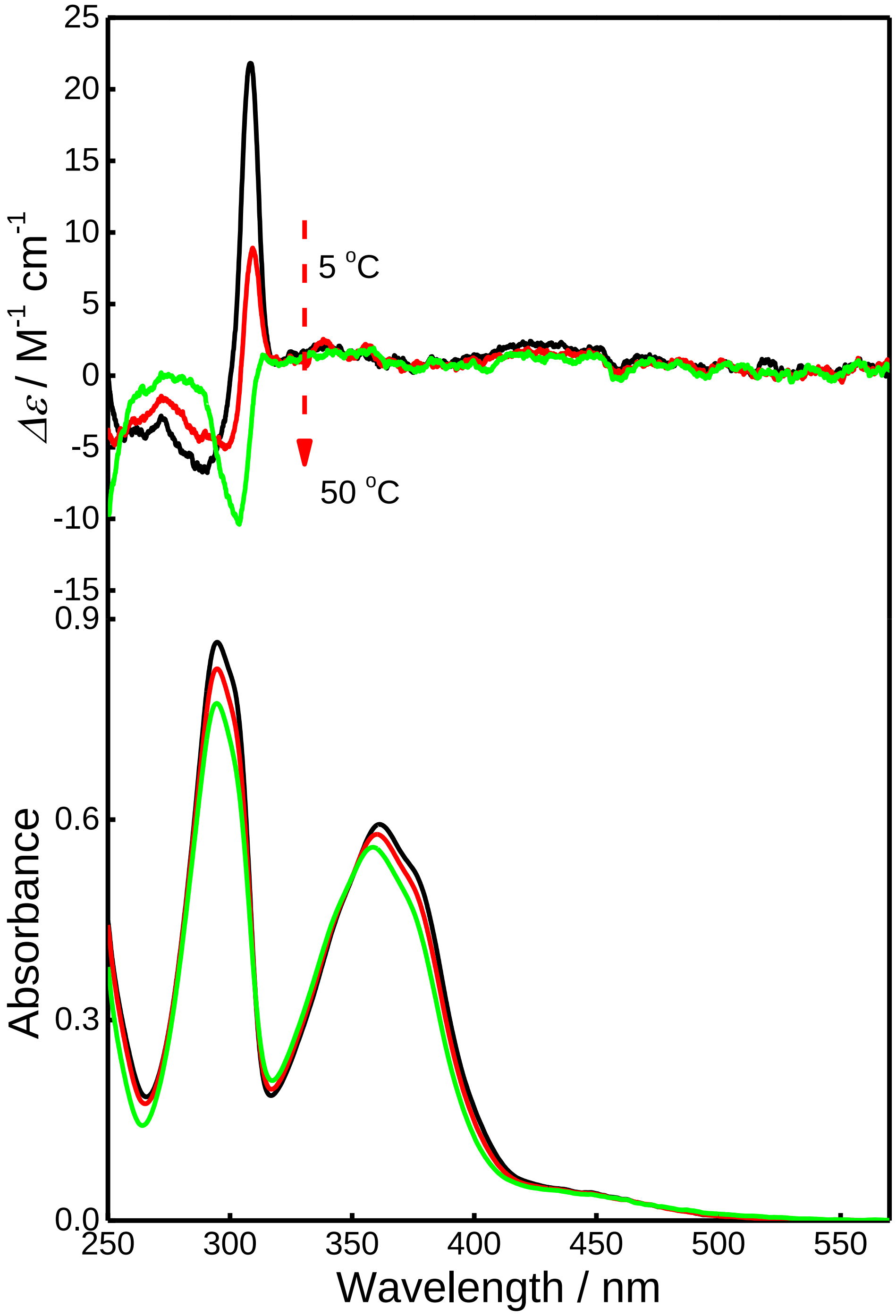

Supplementary Fig. 89 │ V-T CD spectra of *trans*-(*in*-R_p/*out*-S_p)-**MUJ1** (0.03 mM) in tetrahydrofuran at 5 °C (black), 25 °C (red) and 50 °C (green) respectively.

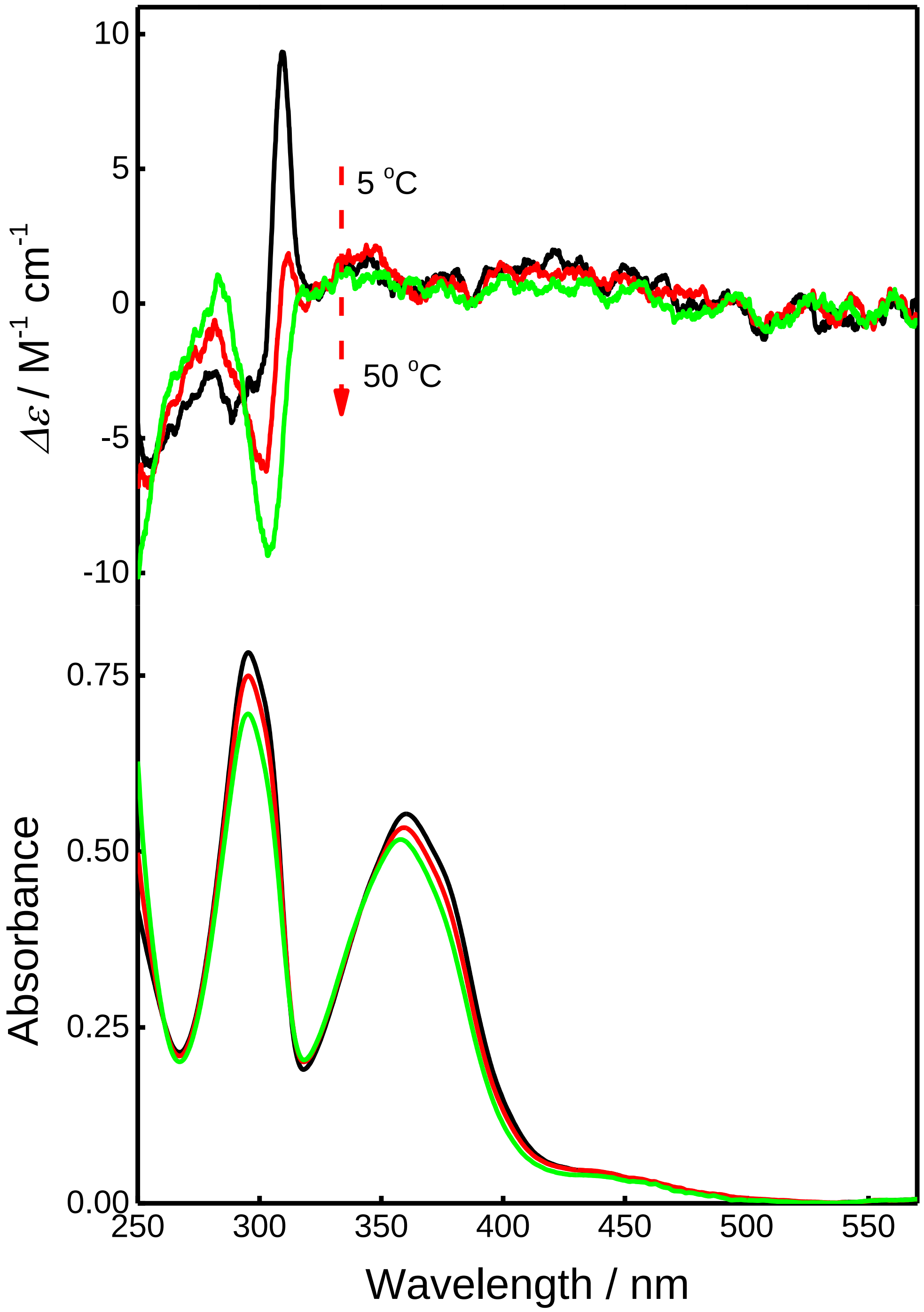

Supplementary Fig. 90 │ V-T CD spectra of *trans*-(*in*-R_p/*out*-S_p)-**MUJ1** (0.03 mM) in chloroform at 5 °C (black), 25 °C (red) and 50 °C (green) respectively.

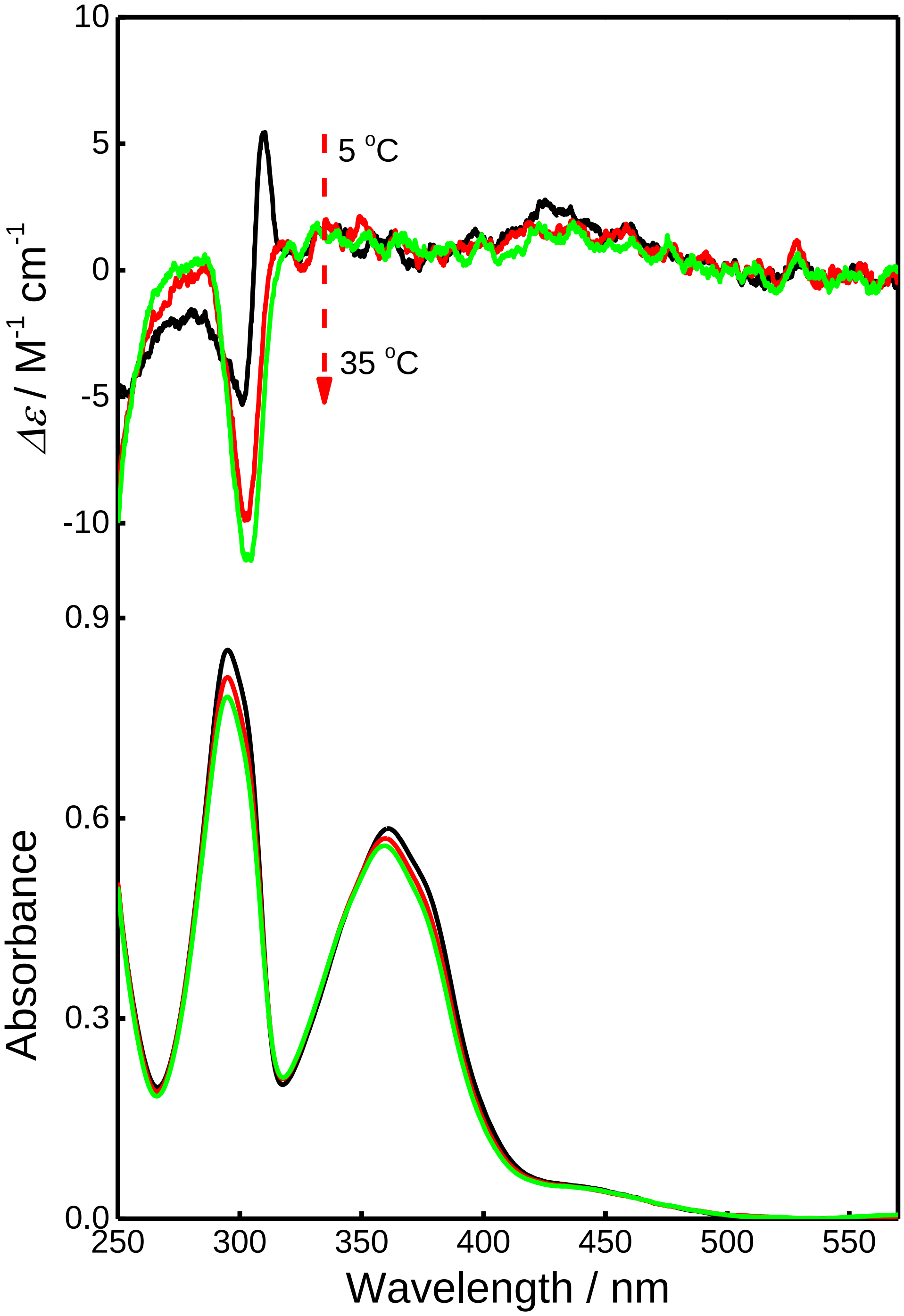

Supplementary Fig. 91 │ V-T CD spectra of *trans*-(*in*-R_p/*out*-S_p)-**MUJ1** (0.03 mM) in dichloromethane at 5 ºC (black), 25 ºC (red) and 35 ºC (green) respectively.

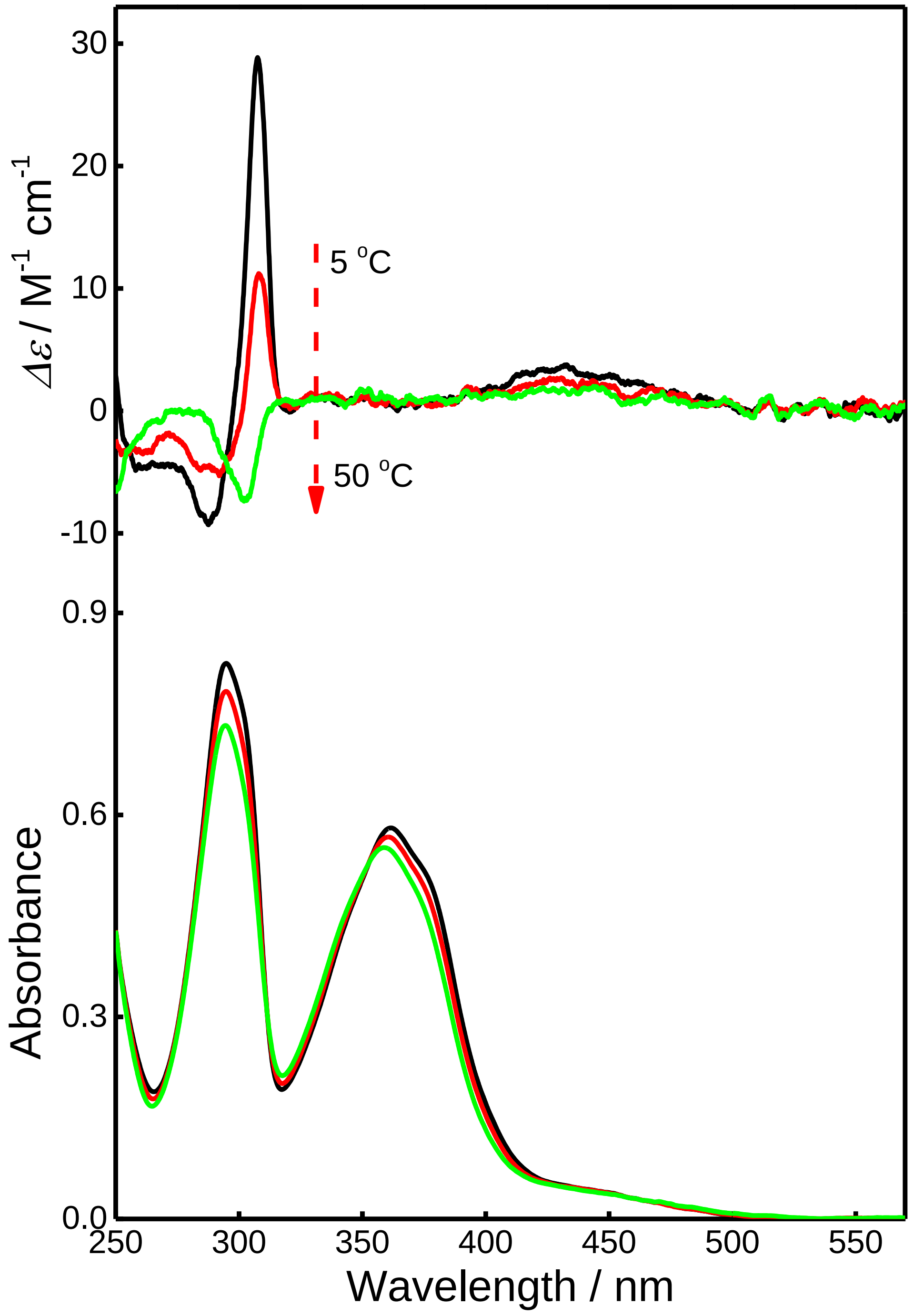

Supplementary Fig. 92 │ V-T CD spectra of *trans*-(*in*-R_p/*out*-S_p)-**MUJ1** (0.03 mM) in acetonitrile at 5 °C (black), 25 °C (red) and 50 °C (green) respectively.

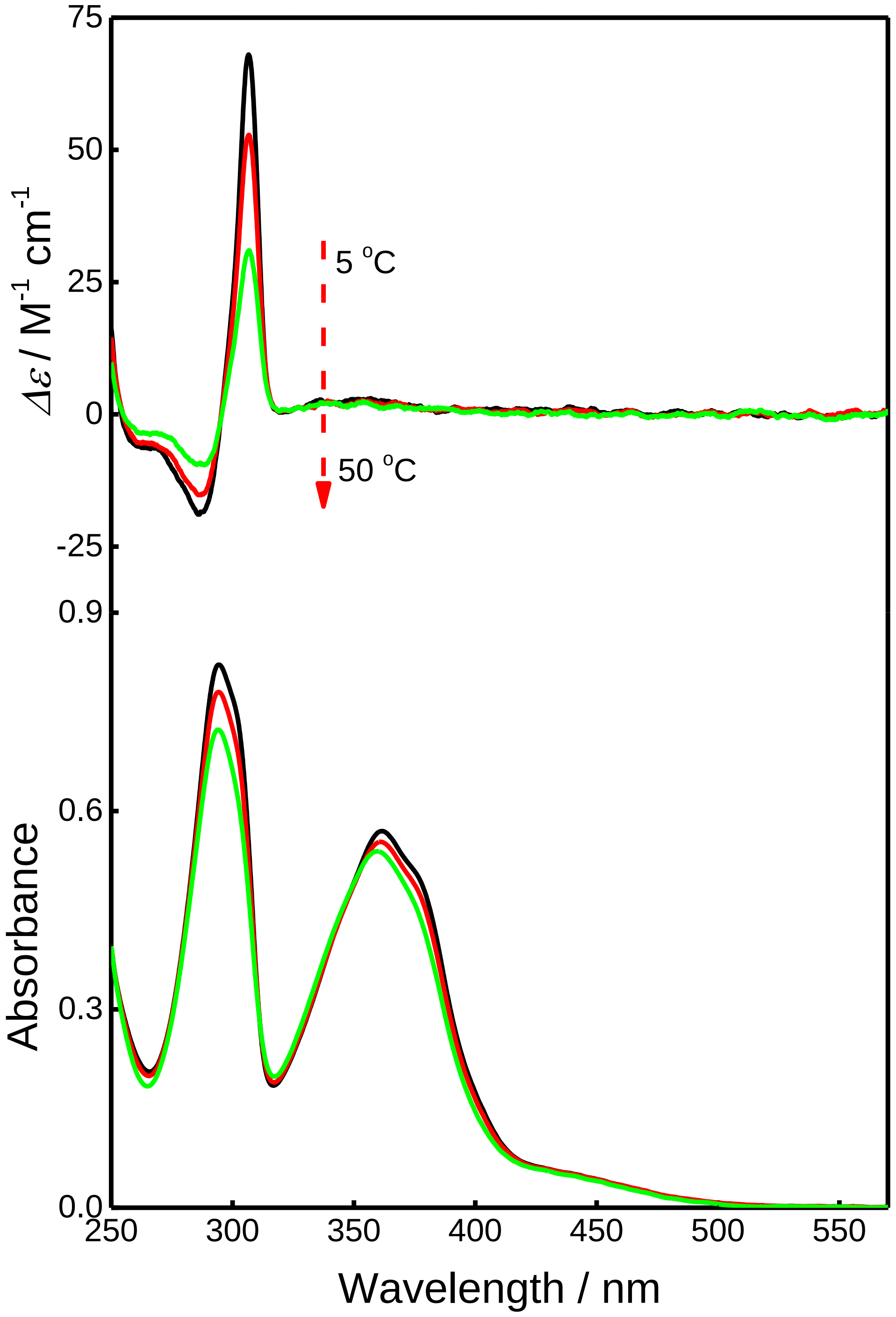

Supplementary Fig. 93 │ V-T CD spectra of *trans*-(*in*-R_p/*out*-S_p)-**MUJ1** (0.03 mM) in methanol at 5 °C (black), 25 °C (red) and 50 °C (green) respectively.

13. VT CD Spectra of *trans*-(*in*-R_p/*out*-S_p)-MUJ2

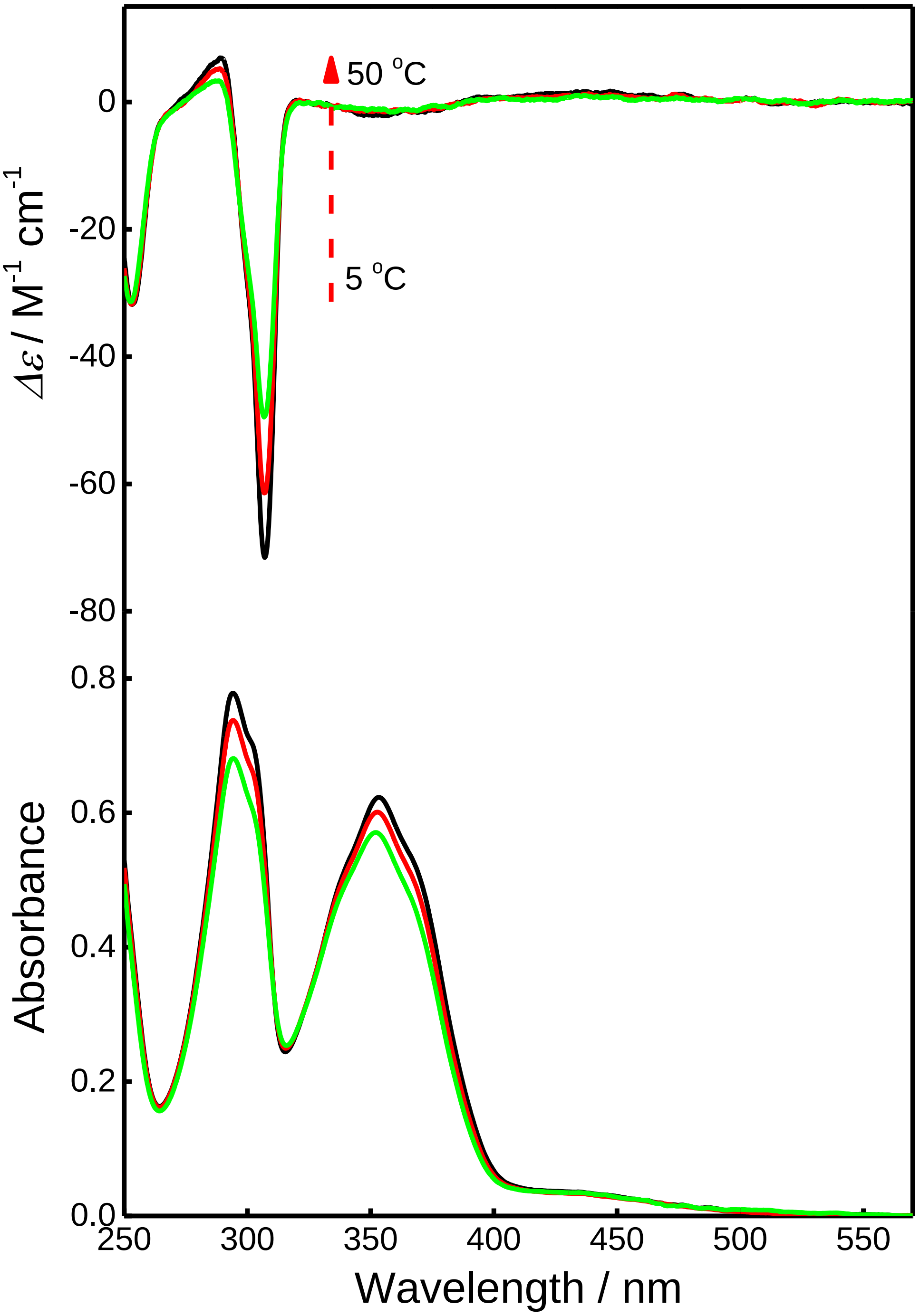

Supplementary Fig. 94 │ V-T CD spectra of *trans*-(*in*-R_p/*out*-S_p)-**MUJ2** (0.046 mM) in *n*-hexane at 5 °C (black), 25 °C (red) and 50 °C (green) respectively.

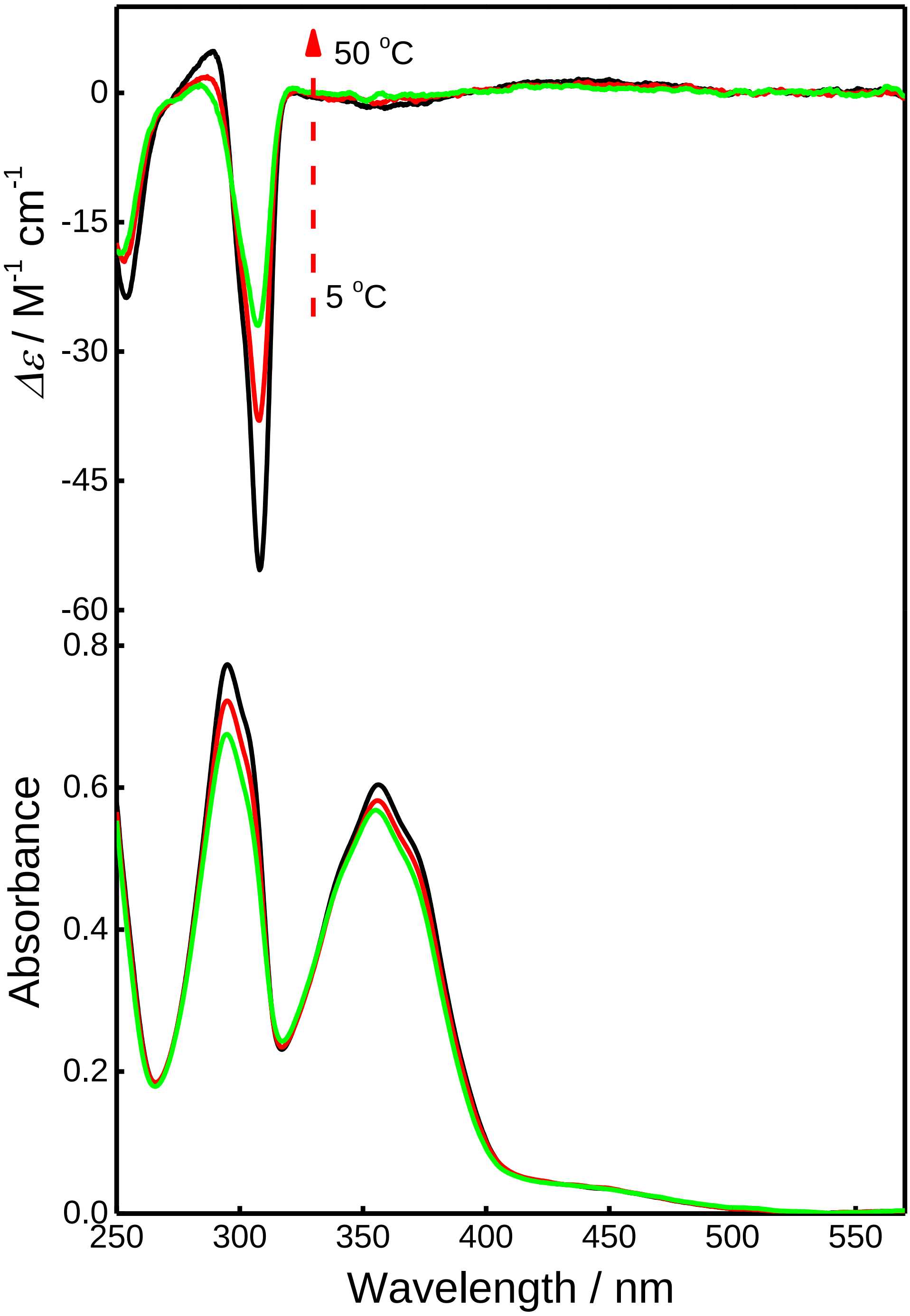

Supplementary Fig. 95 │ V-T CD spectra of *trans*-(*in*-R_p/*out*-S_p)-**MUJ2** (0.075 mM) in decahydronaphthalene at 5 °C (black), 25 °C (red) and 50 °C (green) respectively.

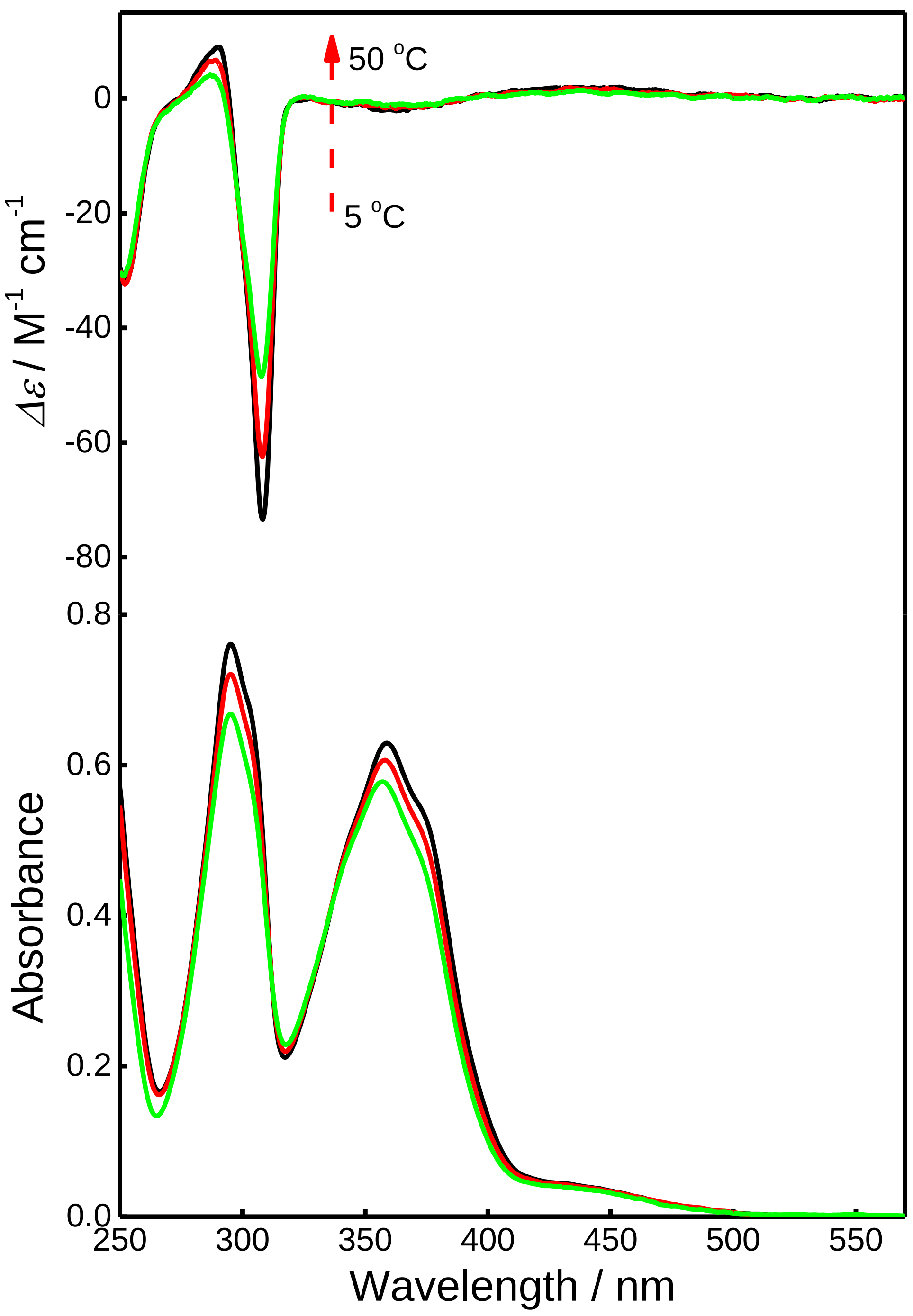

Supplementary Fig. 96 │ V-T CD spectra of *trans*-(*in*-R_p/*out*-S_p)-**MUJ2** (0.046 mM) in tetrahydrofuran at 5 ºC (black), 25 ºC (red) and 50 ºC (green) respectively.

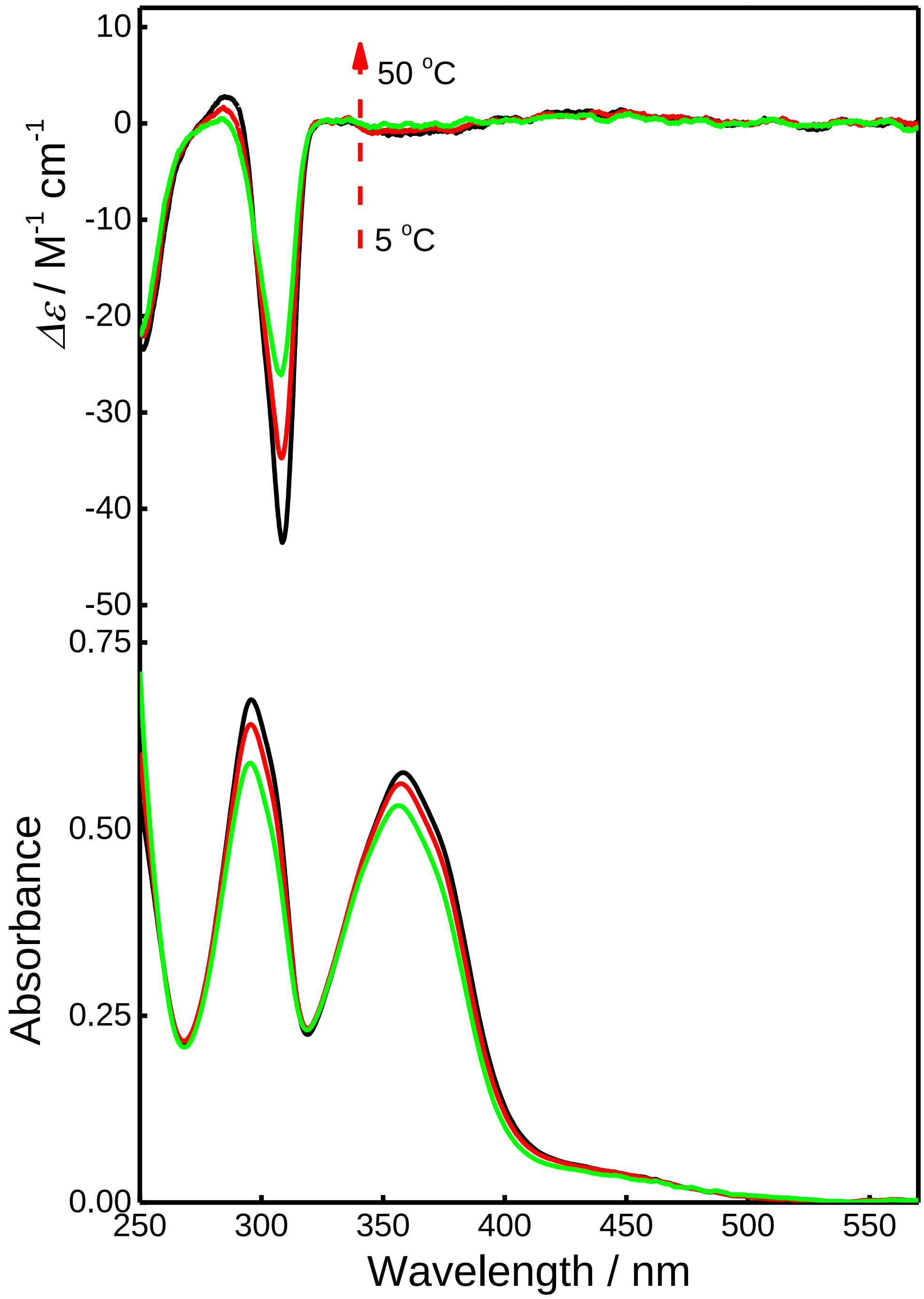

Supplementary Fig. 97 │ V-T CD spectra of *trans*-(*in*-R_p/*out*-S_p)-**MUJ2** (0.046 mM) in chloroform at 5 ºC (black), 25 ºC (red) and 50 ºC (green) respectively.

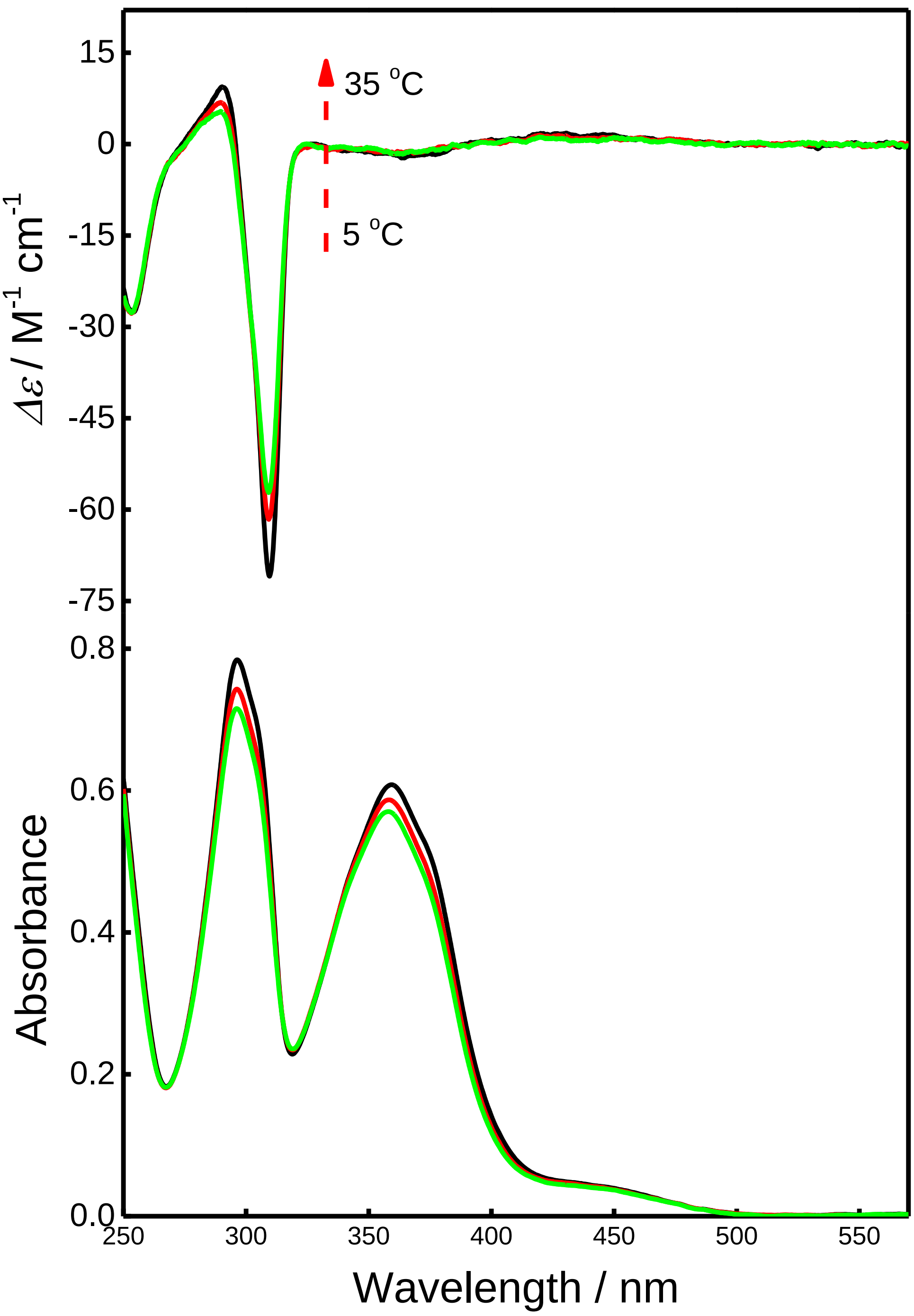

Supplementary Fig. 98 │ V-T CD spectra of *trans*-(*in*-R_p/*out*-S_p)-**MUJ2** (0.046 mM) in dichloromethane at 5 ºC (black), 25 ºC (red) and 35 ºC (green) respectively.

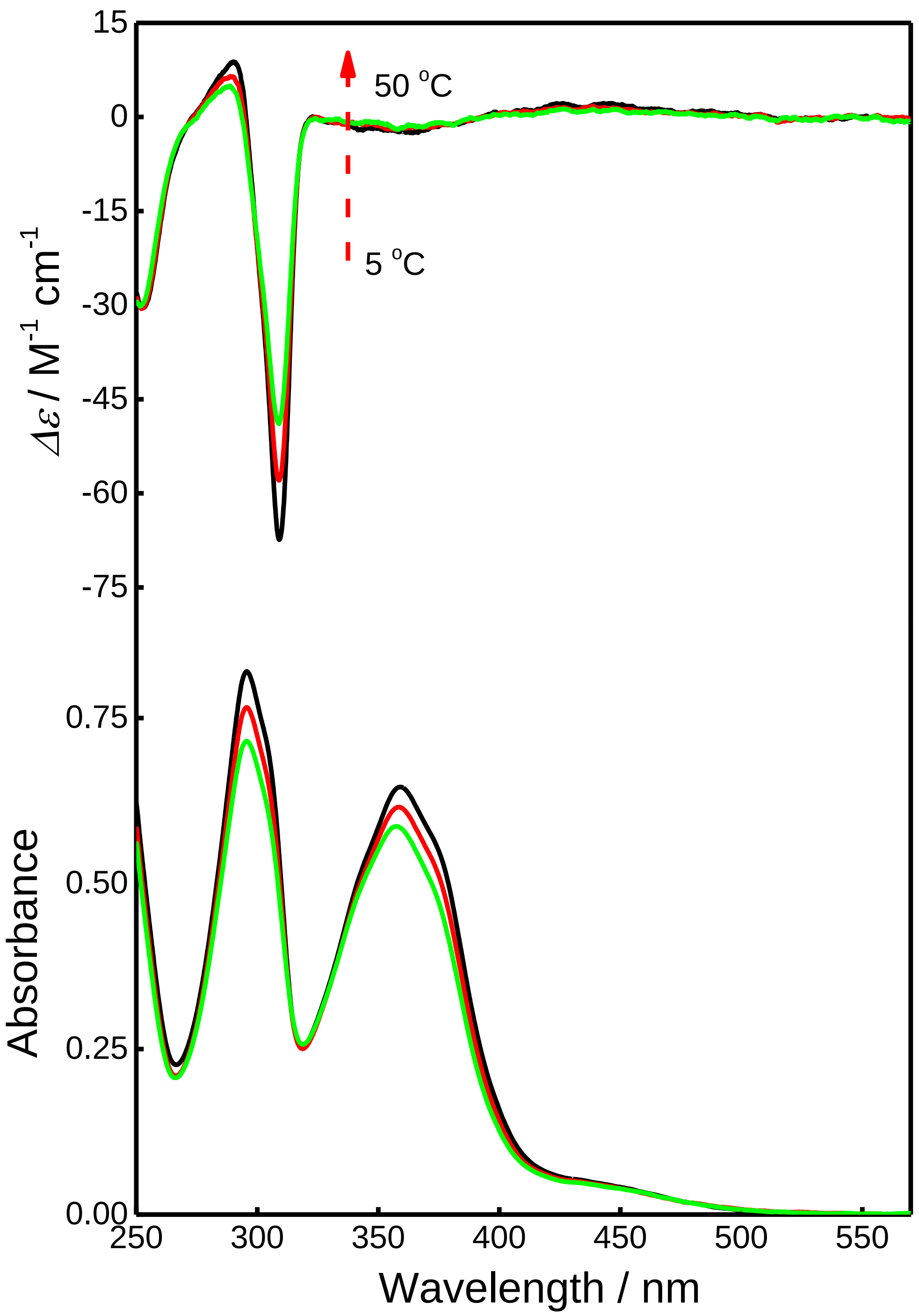

Supplementary Fig. 99 | V-T CD spectra of *trans*-(*in*-R_p/*out*-S_p)-**MUJ2** (0.046 mM) in acetonitrile at 5 °C (black), 25 °C (red) and 50 °C (green) respectively.

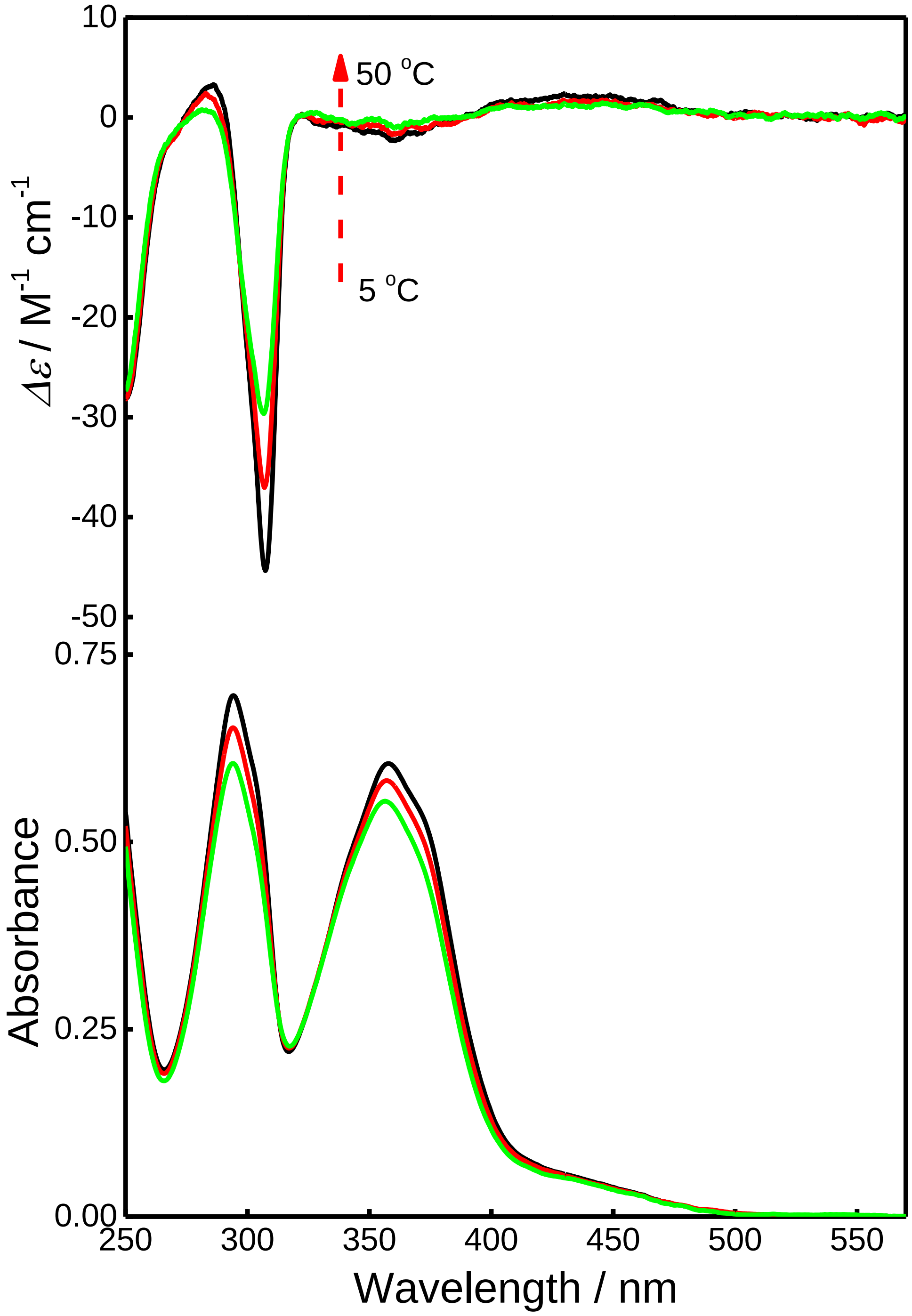

Supplementary Fig. 100 │ V-T CD spectra of *trans*-(*in*-R_p/*out*-S_p)-**MUJ2** (0.046 mM) in methanol at 5 ºC (black), 25 ºC (red) and 50 ºC (green) respectively.

14. VT CD Spectra of *trans*-(*in*-R_p/*out*-S_p)-MUJ3

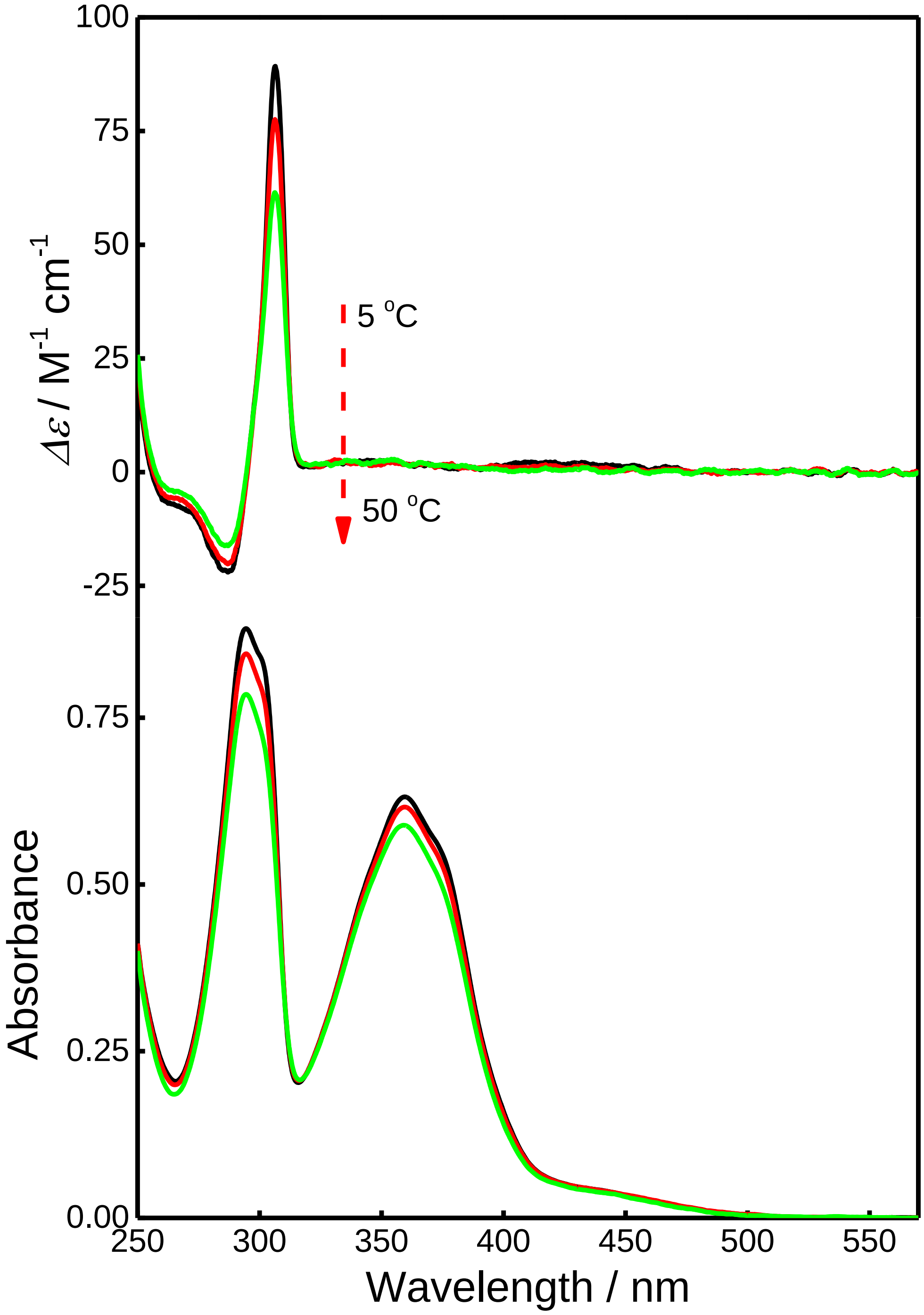

Supplementary Fig. 101 │ V-T CD spectra of *trans*-(*in*-R_p/*out*-S_p)-**MUJ3** (0.030 mM) in *n*-hexane at 5 °C (black), 25 °C (red) and 50 °C (green) respectively.

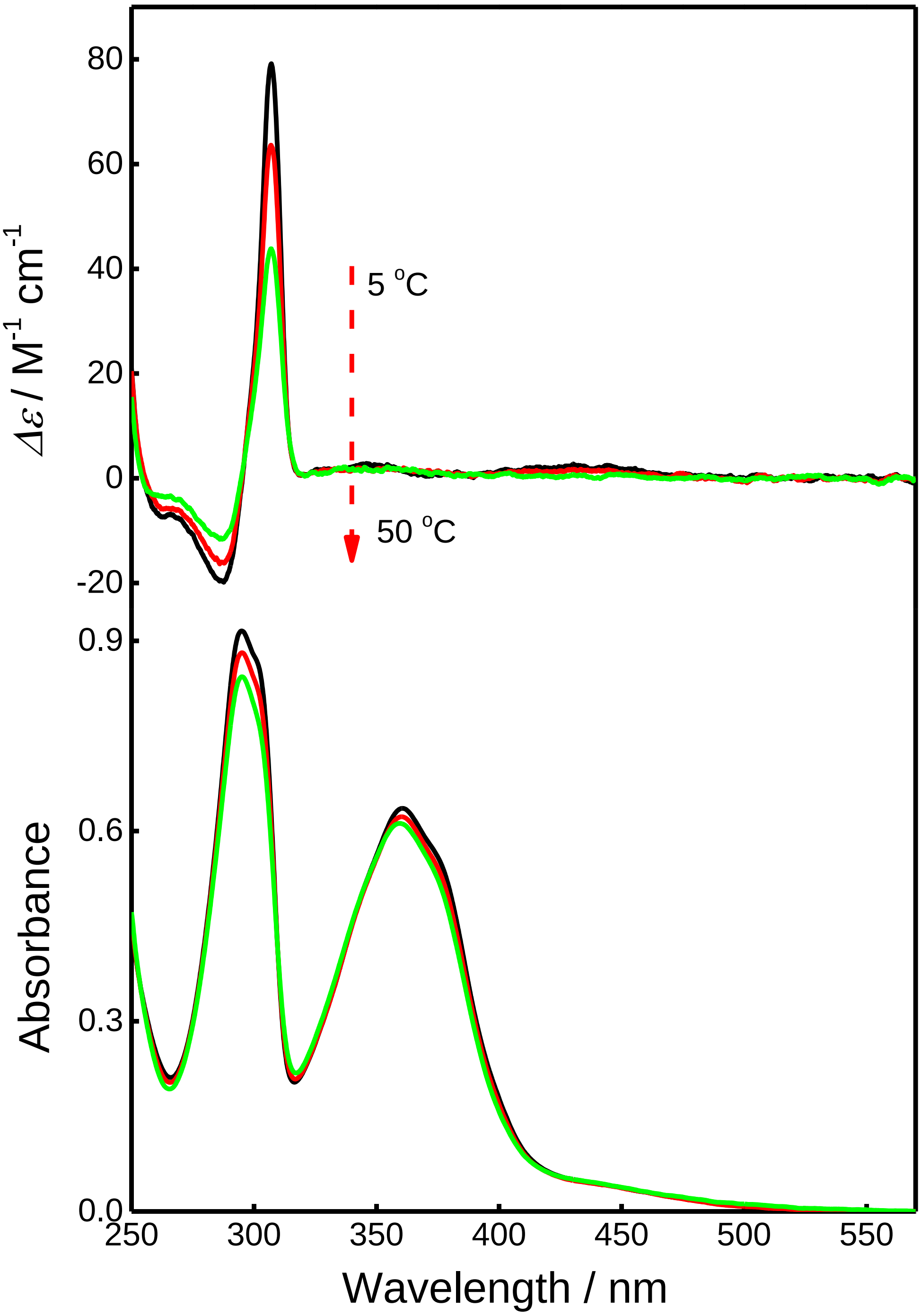

Supplementary Fig. 102 │ V-T CD spectra of *trans*-(*in*-R_p/*out*-S_p)-**MUJ3** (0.030 mM) in decahydronaphthalene at 5 ºC (black), 25 ºC (red) and 50 ºC (green) respectively.

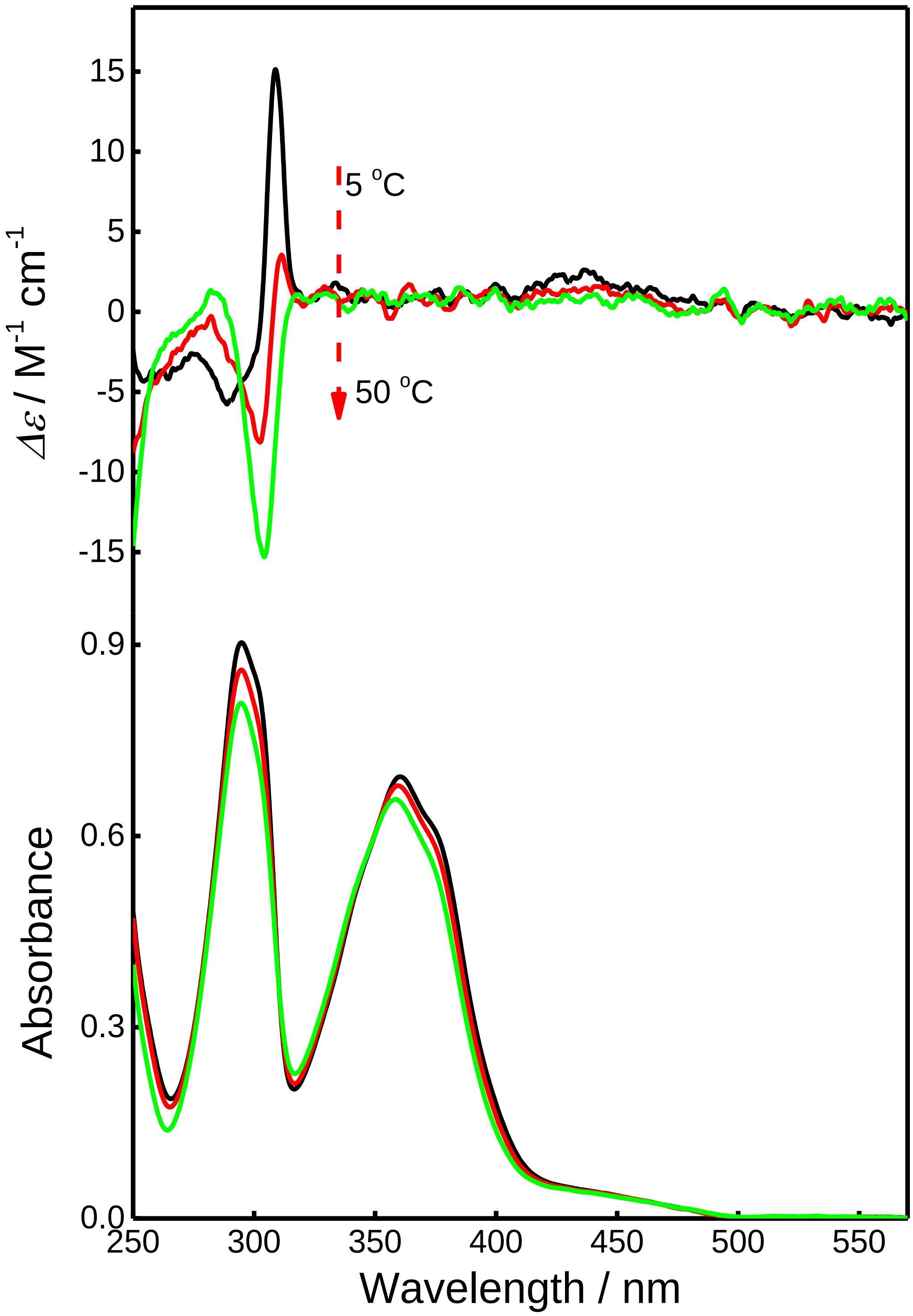

Supplementary Fig. 103 │ V-T CD spectra of *trans*-(*in*-R_p/*out*-S_p)-**MUJ3** (0.030 mM) in tetrahydrofuran at 5 ºC (black), 25 ºC (red) and 50 ºC (green) respectively.

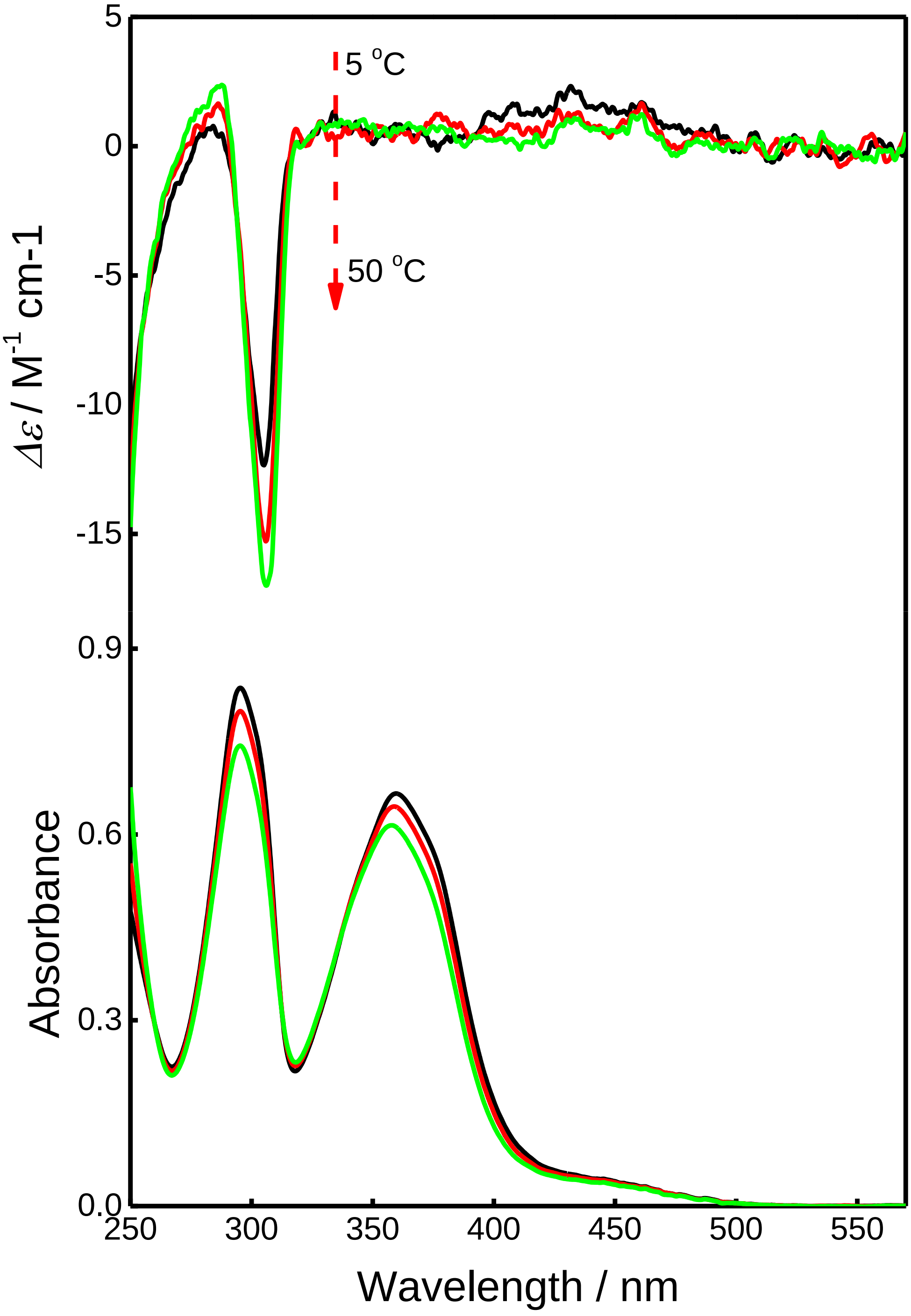

Supplementary Fig. 104 │ V-T CD spectra of *trans*-(*in*-R_p/*out*-S_p)-**MUJ3** (0.030 mM) in chloroform at 5 °C (black), 25 °C (red) and 50 °C (green) respectively.

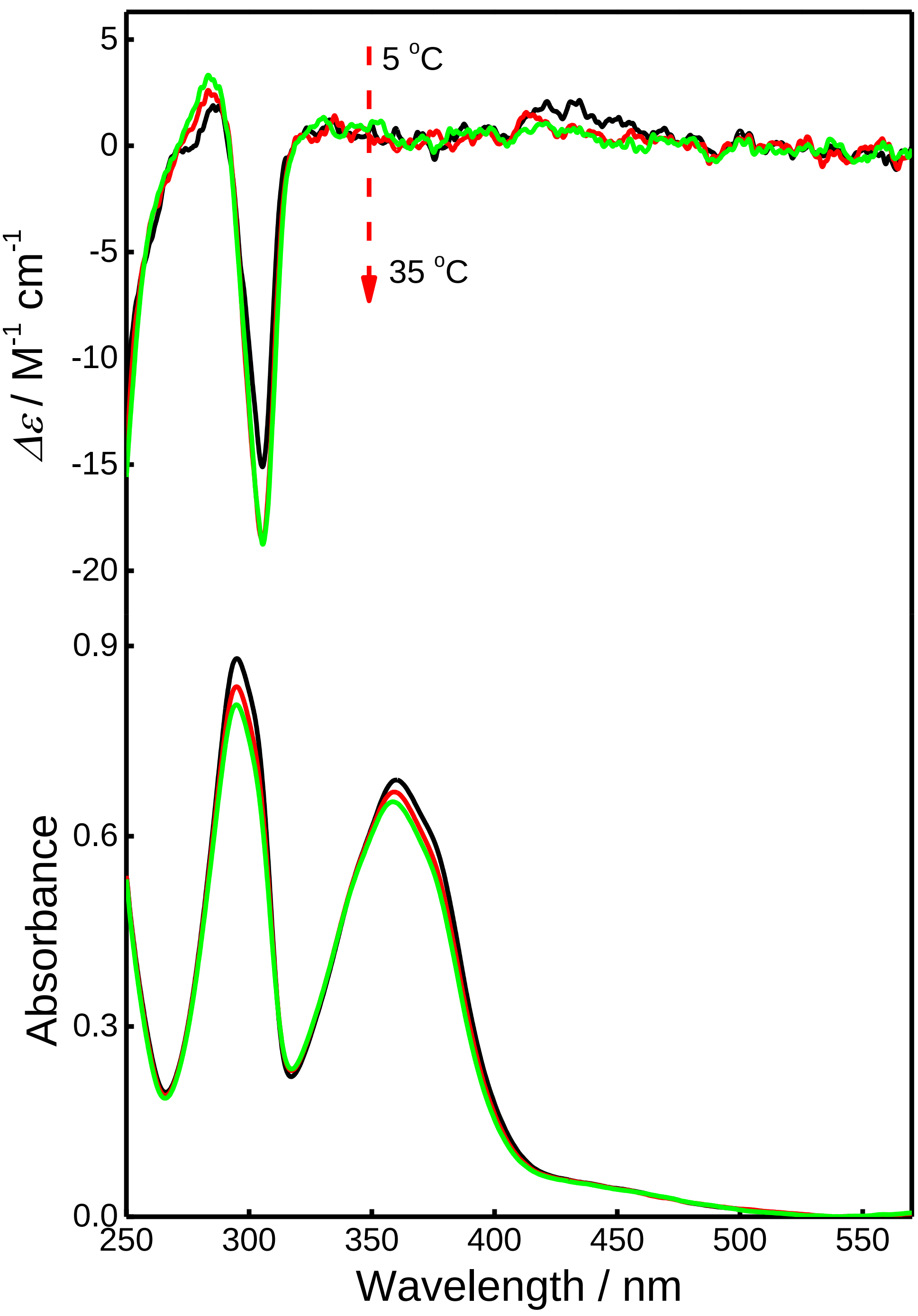

Supplementary Fig. 105 │ V-T CD spectra of *trans*-(*in*-R_p/*out*-S_p)-**MUJ3** (0.030 mM) in dichloromethane at 5 °C (black), 25 °C (red) and 35 °C (green) respectively.

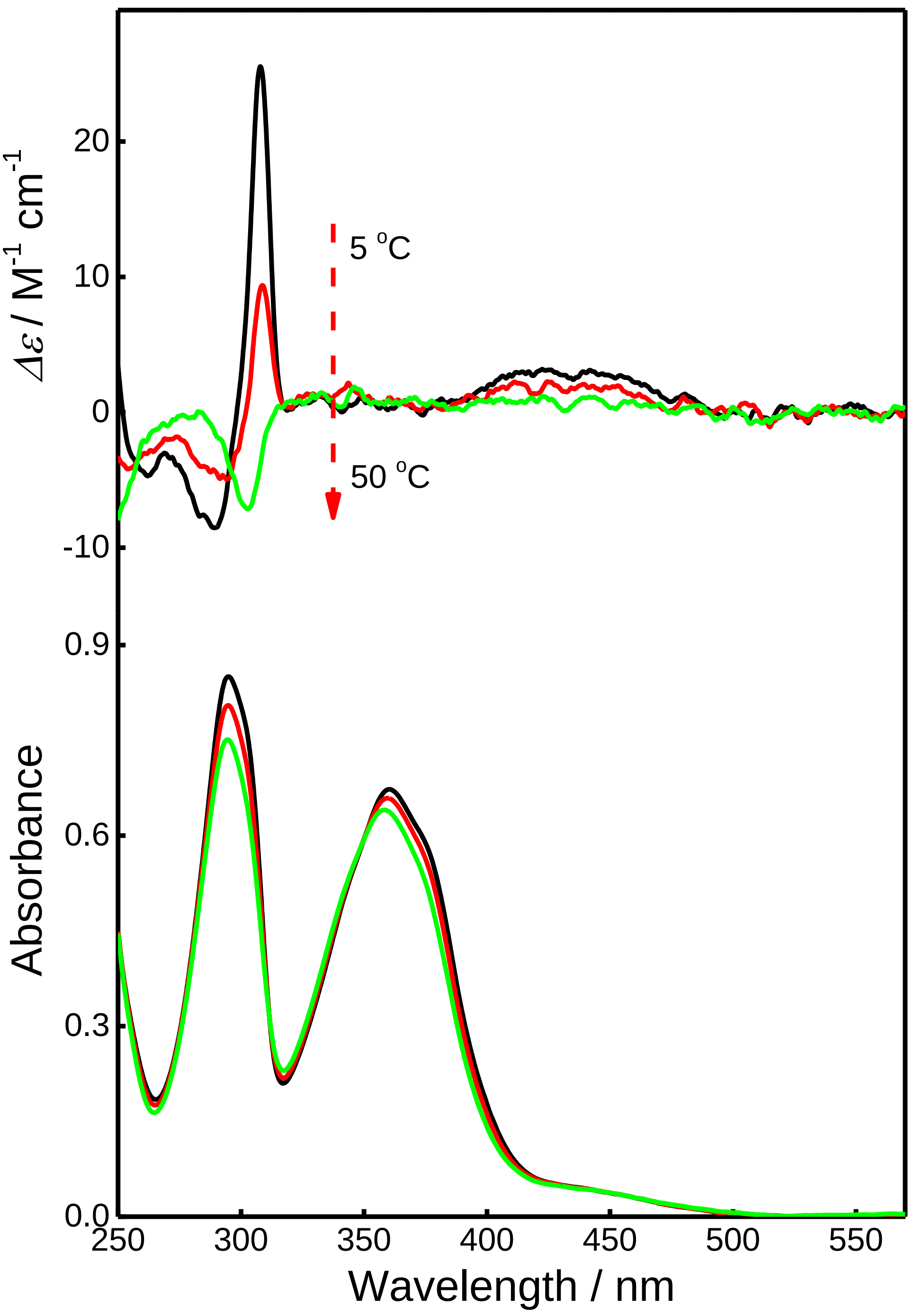

Supplementary Fig. 106 │ V-T CD spectra of *trans*-(*in*-R_p/*out*-S_p)-**MUJ3** (0.030 mM) in acetonitrile at 5 ºC (black), 25 ºC (red) and 50 ºC (green) respectively.

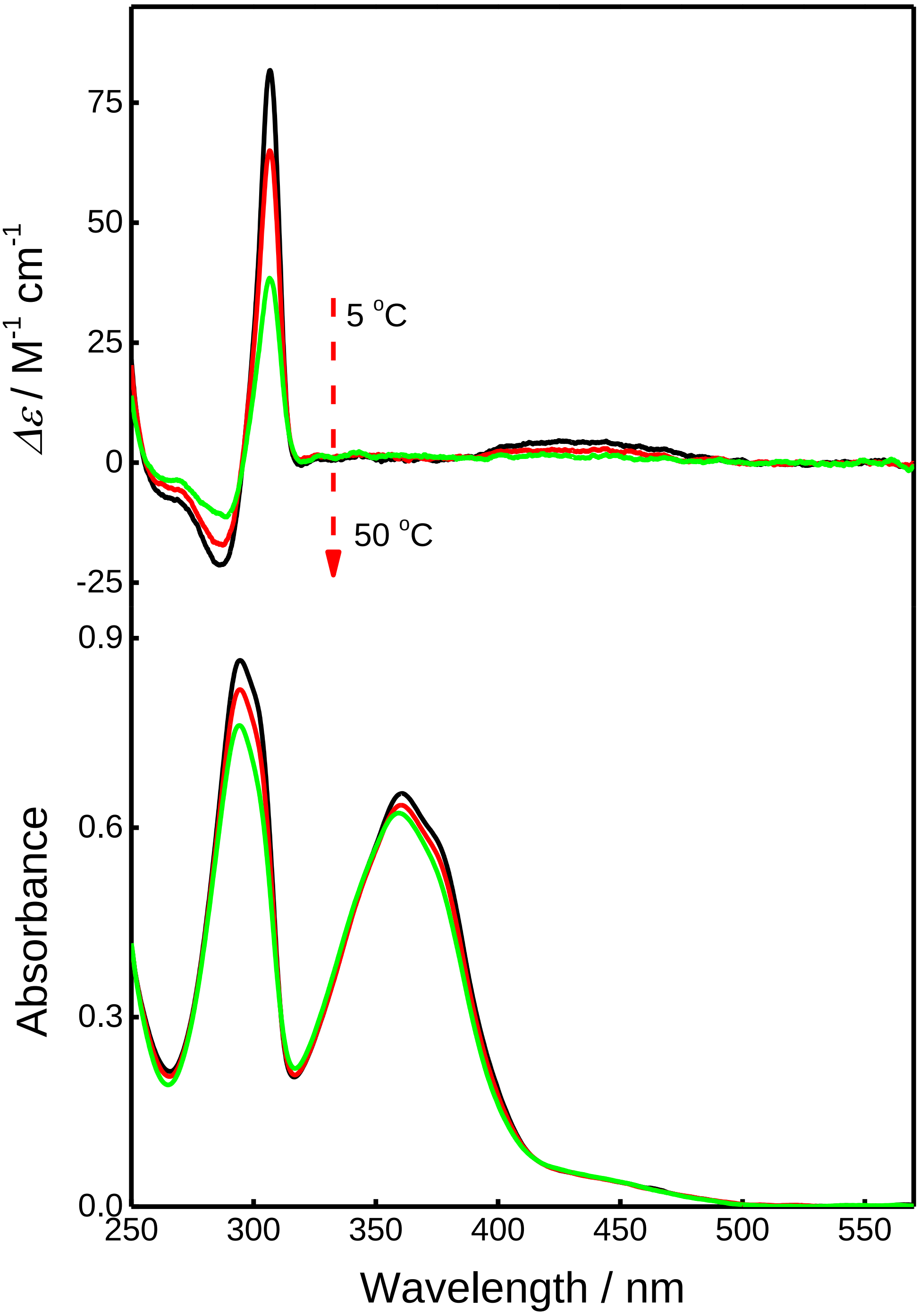

Supplementary Fig. 107 │ V-T CD spectra of *trans*-(*in*-R_p/*out*-S_p)-**MUJ3** (0.030 mM) in methanol at 5 °C (black), 25 °C (red) and 50 °C (green) respectively.

15 Thermodynamic Parameters of the out-to-in Equilibrium

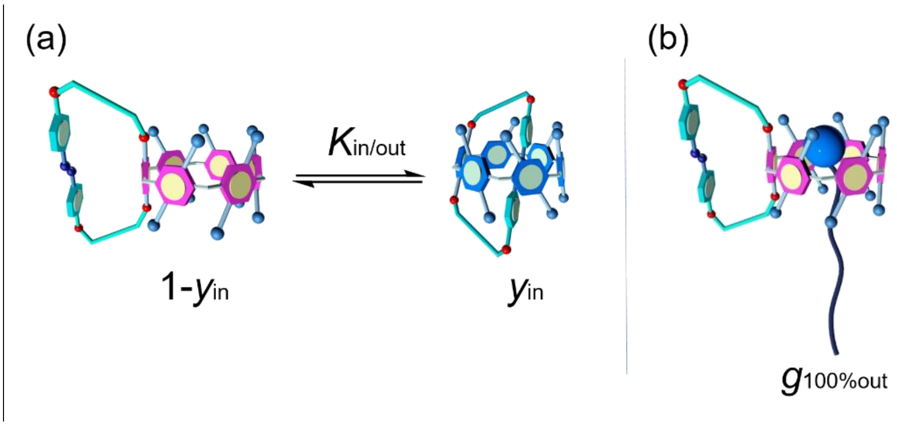

Supplementary Fig. 108 | Schematic representations of (a) the in-out equilibrium of *trans*-(*in*-R_p/*out*-S_p)-**MUJ1** and (b) the penetrative host-guest complex of *trans*-(*in*-R_p/*out*-S_p)-**MUJ1** with **G1**.

$$(1)\ \ y_{in}(-g_{100\%out}) + (1-y_{in})g_{100\%out} = g$$

$$y_{in} = (1-g/g_{100\%out})/2$$

$$(2)\ \ K_{in/out} = y_{in}/(1-y_{in})$$

In this case, the *g* factor of 100% *out* conformers of *trans*-(*in*-R_p/*out*-S_p)-**MUJ1** is estimated by adding excessive **G1** to form saturated *out*-**MUJ1**, and the $g_{100\%in}$ is supposed as $-g_{100\%out}$ in order to estimate the proportion of *in*-**MUJ1** and *out*-**MUJ1** as well as the equilibrium constant $K_{in}/_{out}$ by the ratio of *g* factor extremum at around 310 nm. So the thermodynamic parameters can be calculated by van't Hoff treatment of ln$K_{in/out}$ versus T^{-1}.

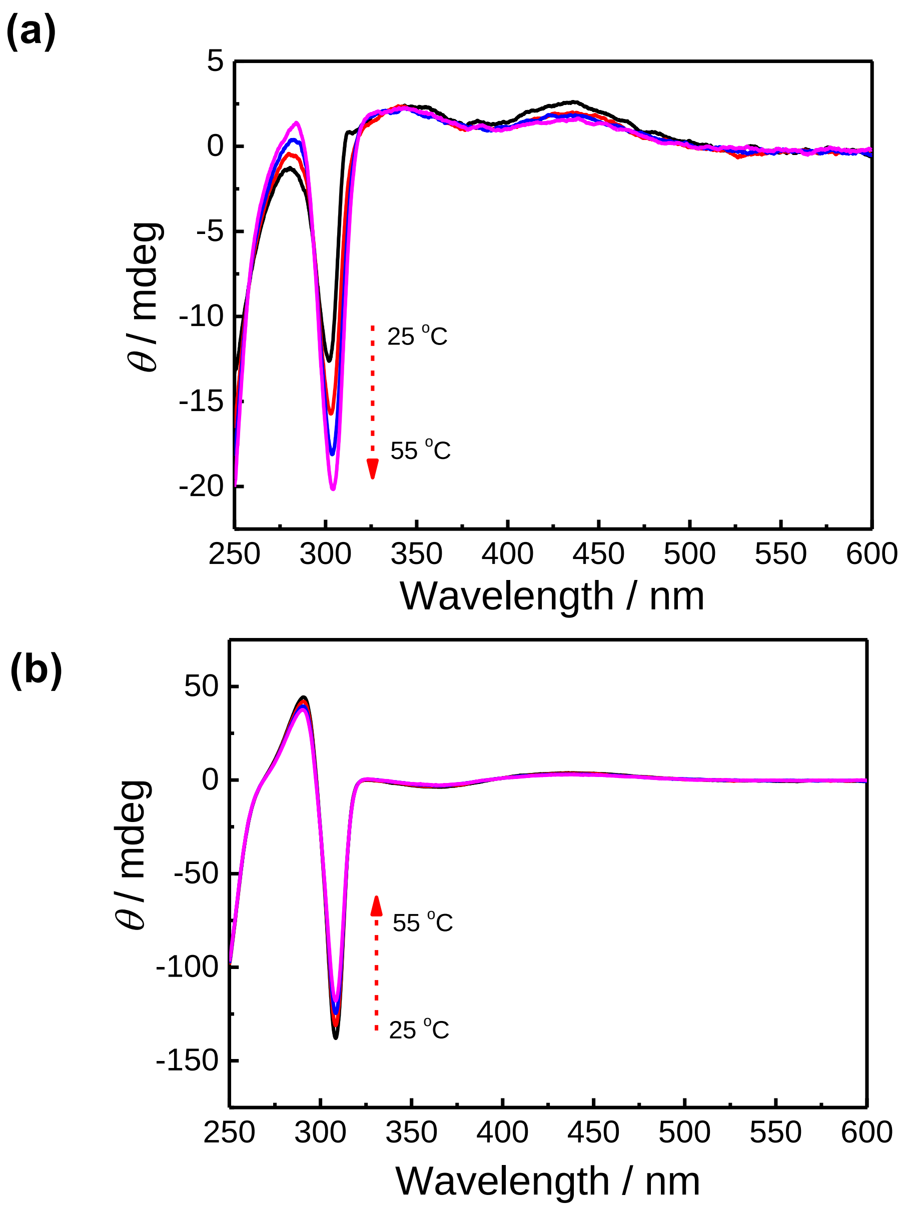

Supplementary Fig. 109 │ VT CD spectra of (a) *trans*-(*in*-R_p/*out*-S_p)-**MUJ1** and (b) *trans*-(*in*-R_p/*out*-S_p)-**MUJ1** with excessive **G1** in chloroform.

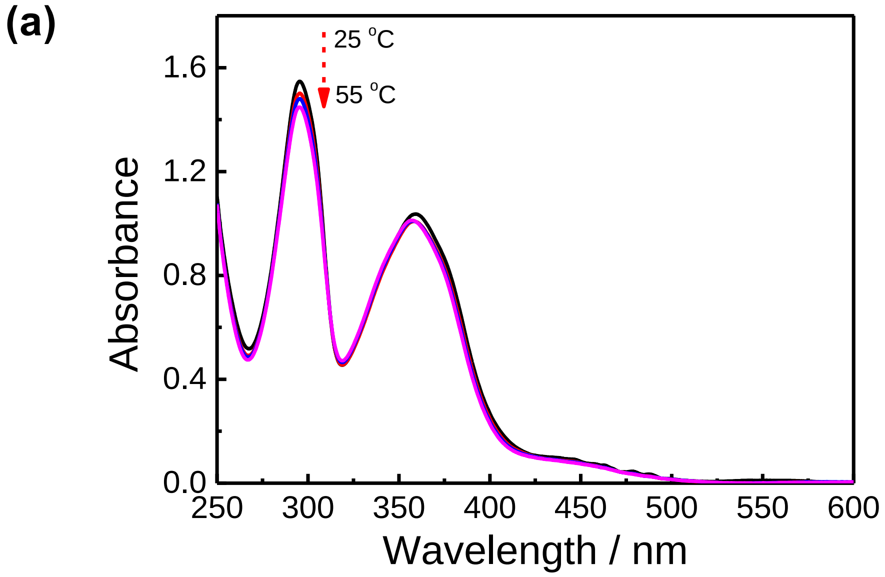

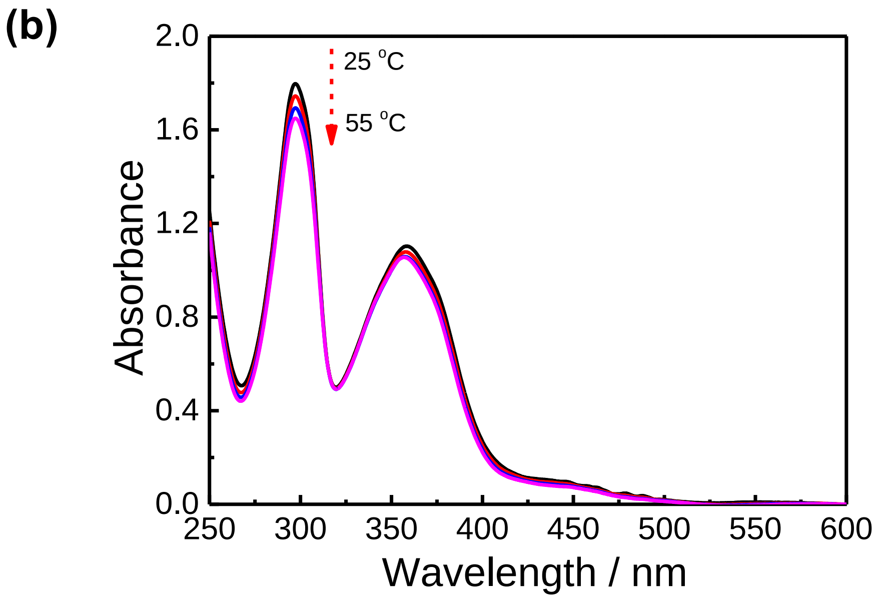

Supplementary Fig. 110 | VT UV-Vis spectra of (a) *trans*-(*in*-R_p/*out*-S_p)-**MUJ1** and (b) *trans*-(*in*-R_p/*out*-S_p)-**MUJ1** with excessive **G1** in chloroform.

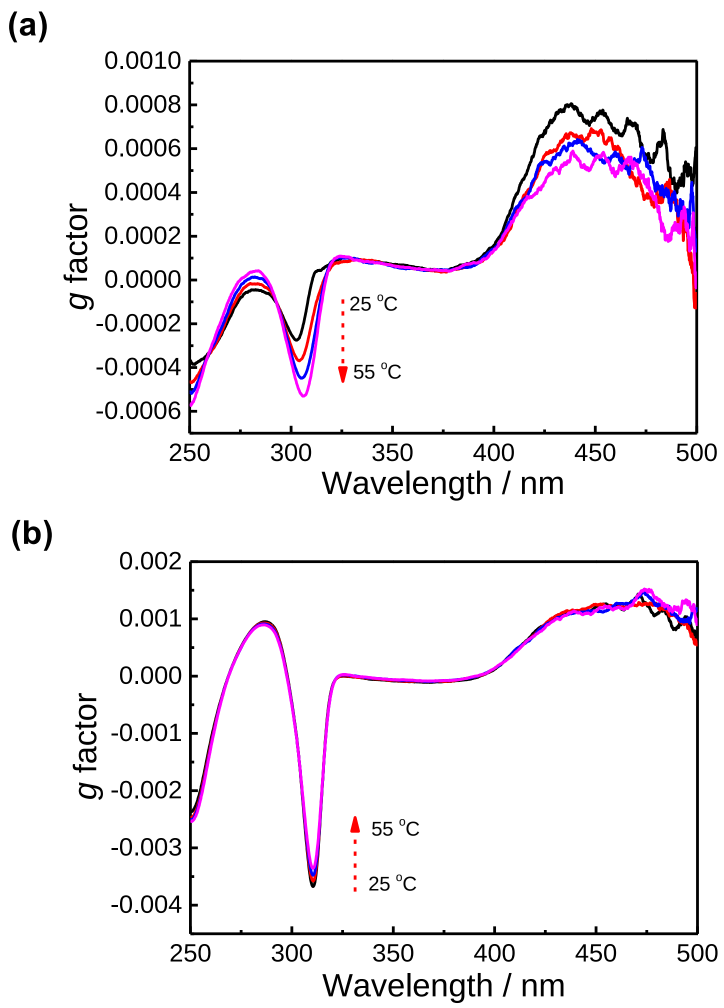

Supplementary Fig. 111 | VT dissymmetry factor (g) spectra of (a) *trans*-(*in*-R_p/*out*-S_p)-**MUJ1** and (b) *trans*-(*in*-R_p/*out*-S_p)-**MUJ1** with excessive **G1** in chloroform.

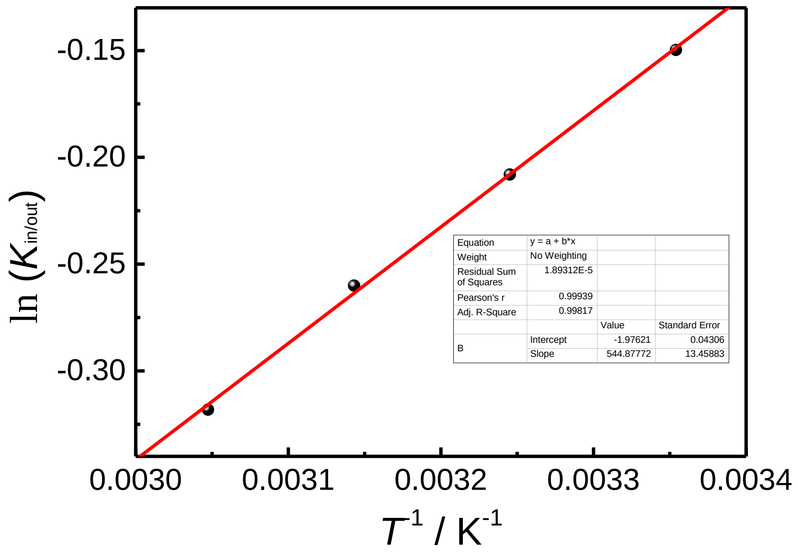

Supplementary Fig. 112 | van't Hoff plot for the in-out equilibrium of *trans*-(*in*-R_p/*out*-S_p)-**MUJ1** at different temperature in chloroform, and thermodynamic parameters $\Delta\Delta H$ = -4.5 KJ mol^{-1}, $\Delta\Delta S$ = -16.5 J mol^{-1} K^{-1} are obtained for the *out*-to-*in* chiral inversion.

16. Temperature-Regulated Light-Driven Chirality Switching

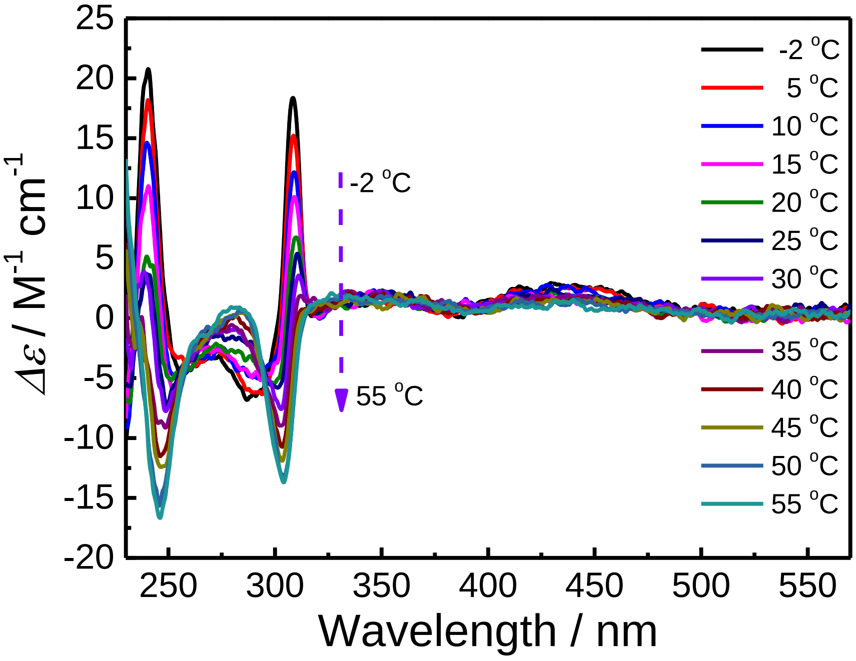

Supplementary Fig. 113 │ V-T CD spectra of (*in*-R_p/*out*-S_p)-**MUJ1** in the PSS at 510 nm in tetrahydrofuran.

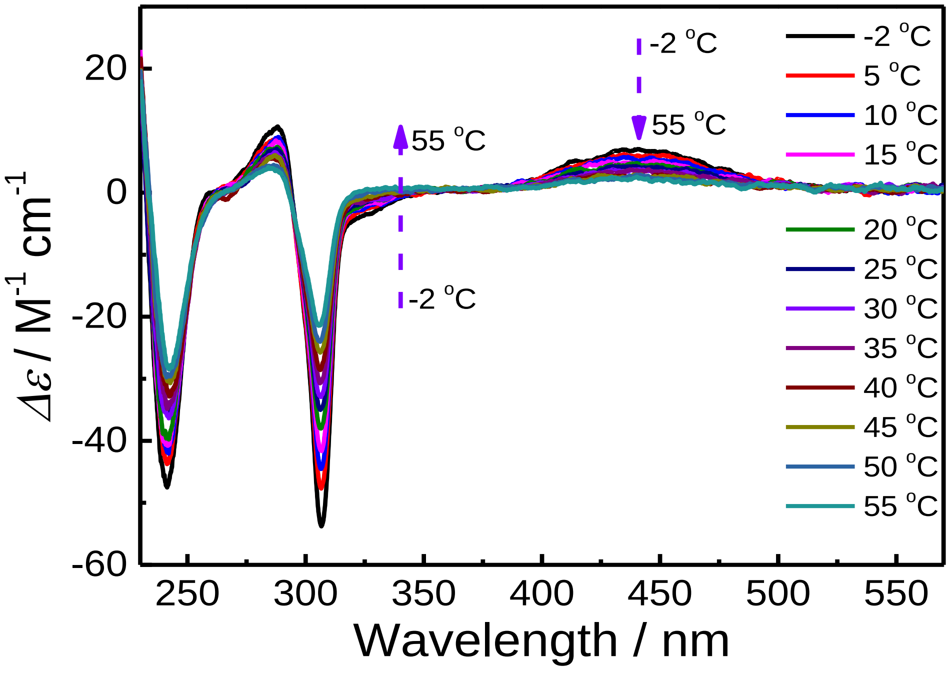

Supplementary Fig. 114 │ V-T CD spectra of (*in*-R_p/*out*-S_p)-**MUJ1** in the PSS at 365 nm in tetrahydrofuran.

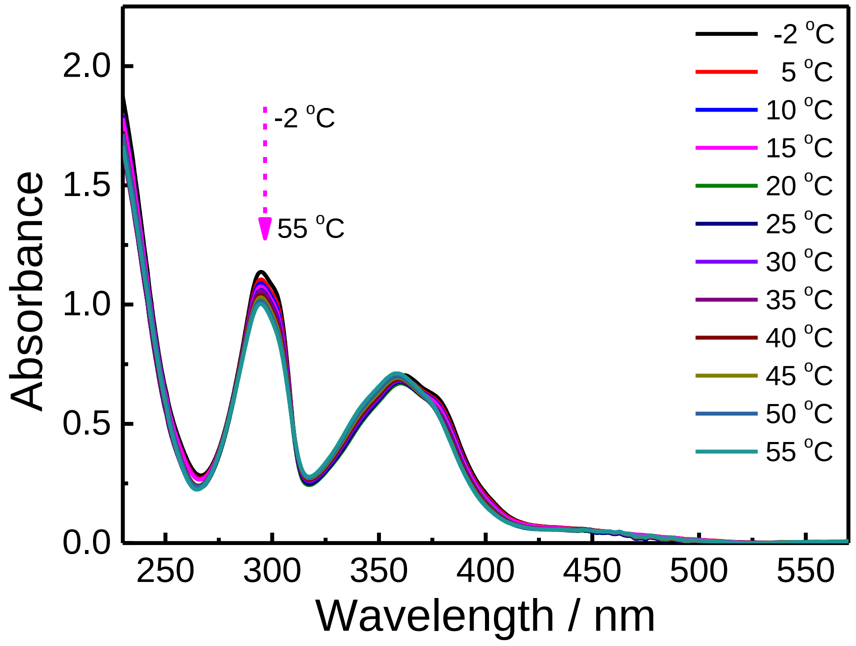

Supplementary Fig. 115 │ V-T absorption spectra of (*in*-R_p/*out*-S_p)-**MUJ1** in the PSS at 510 nm in tetrahydrofuran.

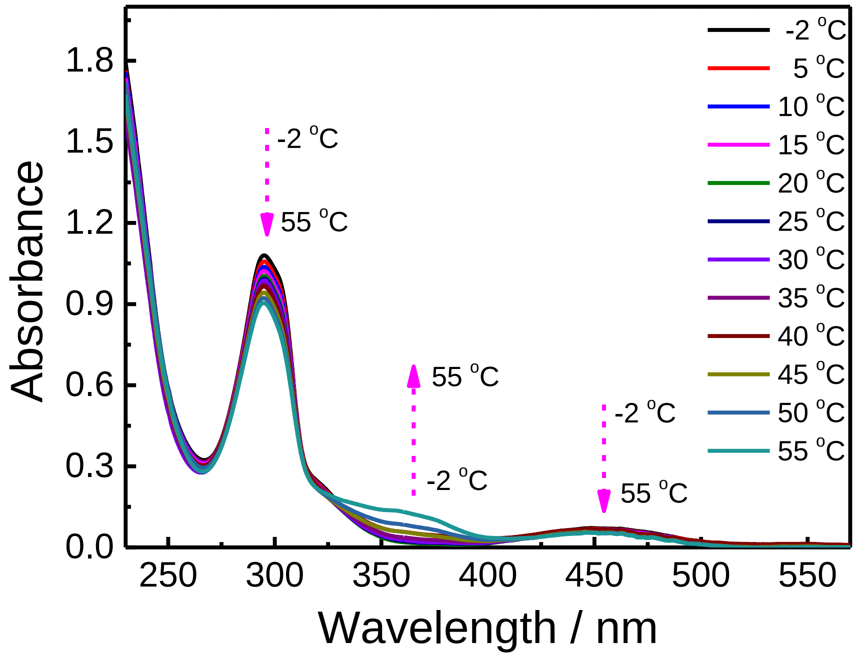

Supplementary Fig. 116 │ V-T absorption spectra of (*in*-R_p/*out*-S_p)-**MUJ1** in the PSS at 365 nm in tetrahydrofuran.

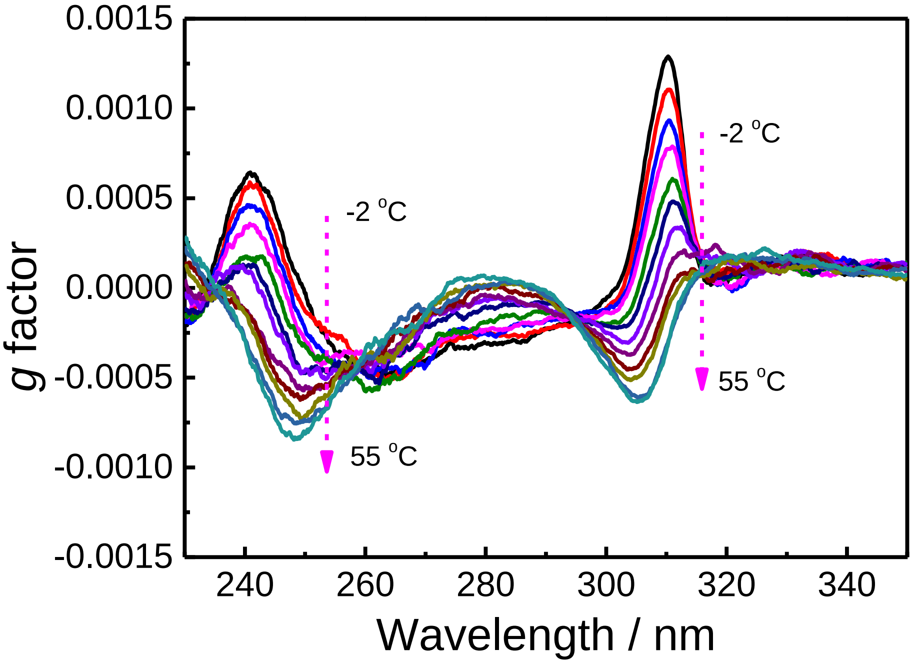

Supplementary Fig. 117 | V-T *g* factor changes of (*in*-R_p/*out*-S_p)-**MUJ1** in the PSS at 510 nm in tetrahydrofuran.

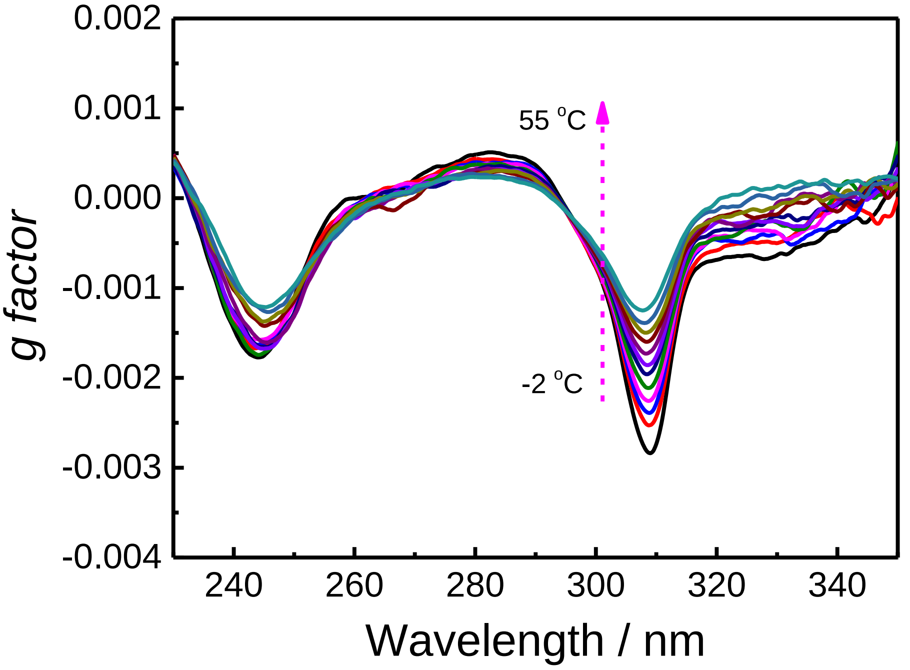

Supplementary Fig. 118 | V-T *g* factor changes of (*in*-R_p/*out*-S_p)-**MUJ1** in the PSS at 365 nm in tetrahydrofuran.

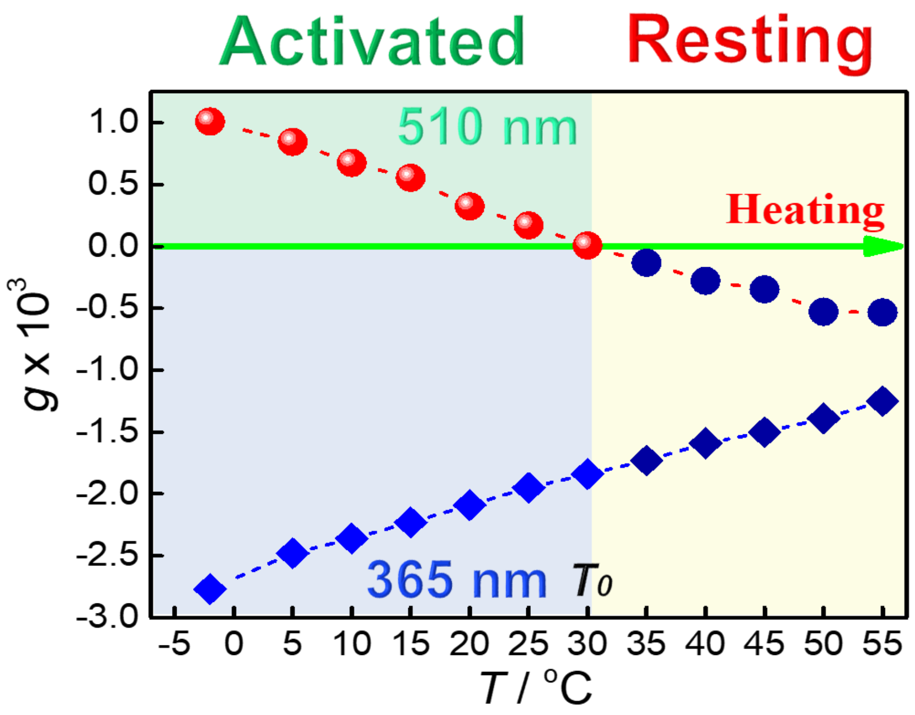

Supplementary Fig. 119 │ V-T anisotropy factor (*g*) changes of (*in*-R_p/*out*-S_p)-**MUJ1** in the PSS at 365 nm (diamond) and 510 nm (circle) detected at 308 nm in tetrahydrofuran. (T_0 = 30.3 °C)

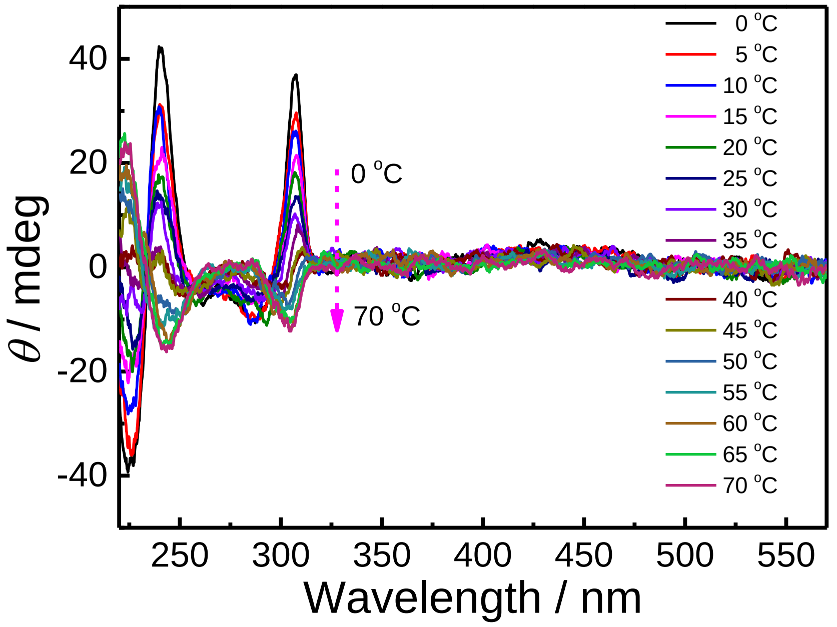

Supplementary Fig. 120 │ V-T CD spectra of (*in*-R_p/*out*-S_p)-**MUJ1** in the PSS at 510 nm in acetonitrile.

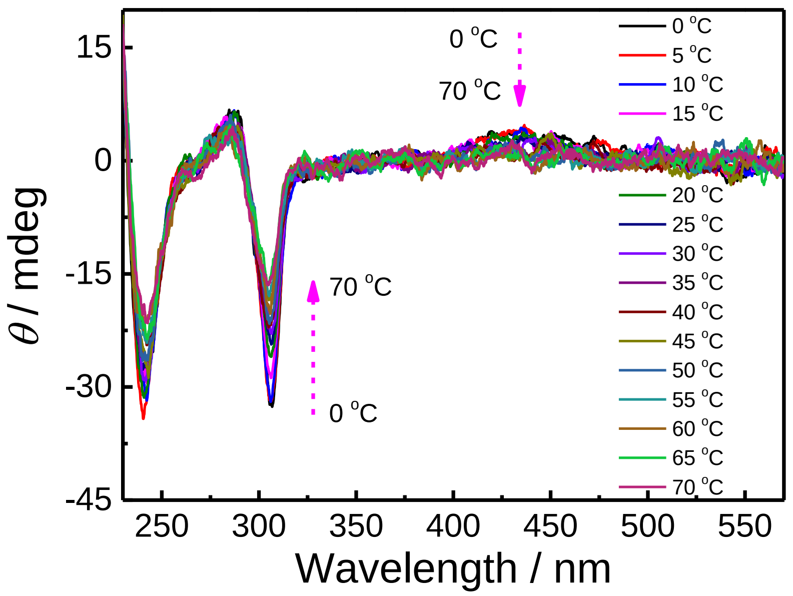

Supplementary Fig. 121 │ V-T CD spectra of (*in*-R_p/*out*-S_p)-**MUJ1** in the PSS at 365 nm in acetonitrile.

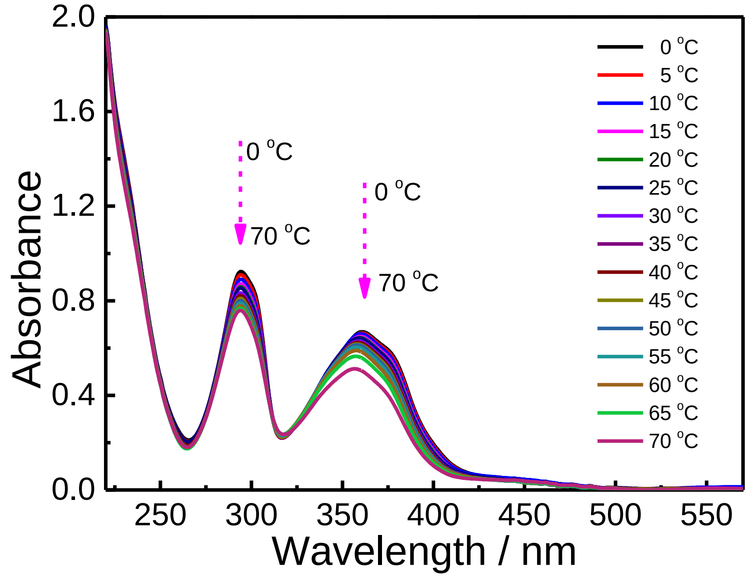

Supplementary Fig. 122 │ V-T absorption spectra of (*in*-R_p/*out*-S_p)-**MUJ1** in the PSS at 510 nm in acetonitrile.

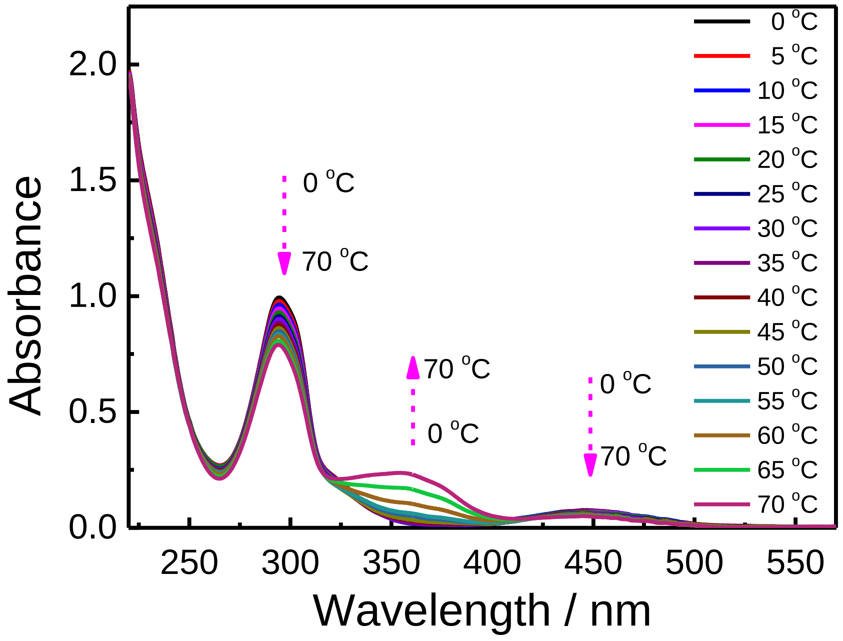

Supplementary Fig. 123 | V-T absorption spectra of (*in*-R_p/*out*-S_p)-**MUJ1** in the PSS at 365 nm in acetonitrile.

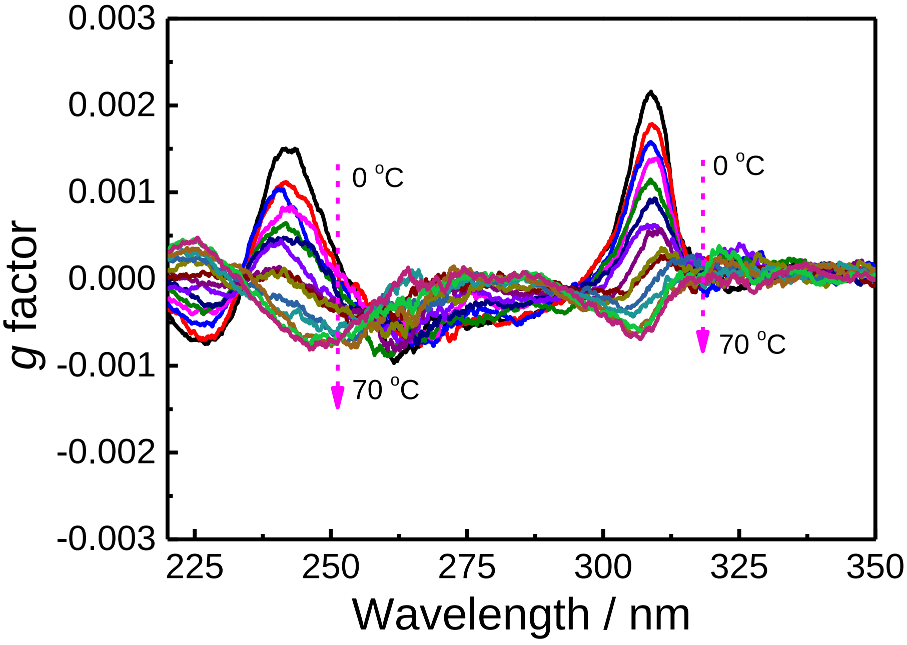

Supplementary Fig. 124 | V-T *g* factor changes of (*in*-R_p/*out*-S_p)-**MUJ1** in the PSS at 510 nm in acetonitrile.

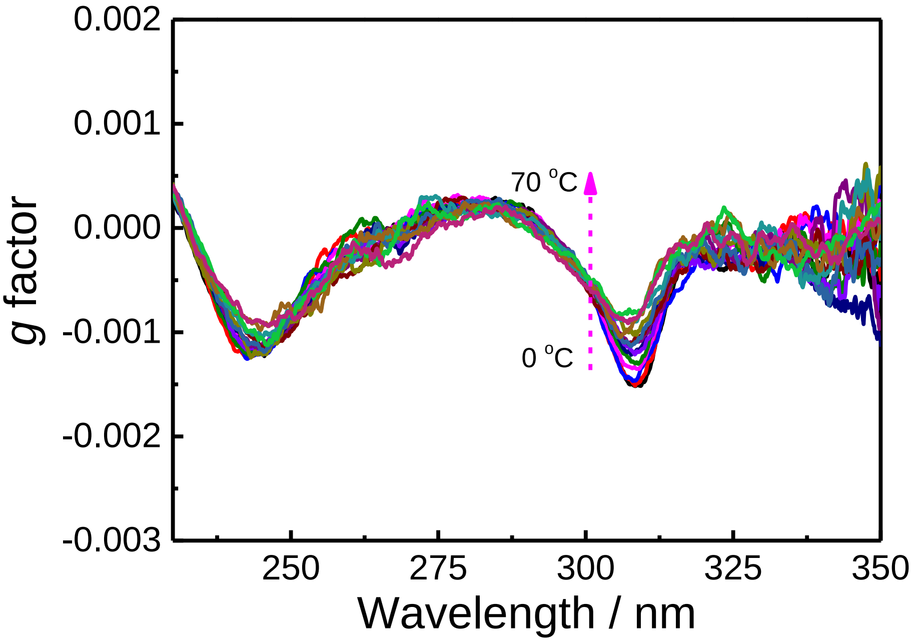

Supplementary Fig. 125 │ V-T g factor changes of (*in*-R_p/*out*-S_p)-**MUJ1** in the PSS at 365 nm in acetonitrile.

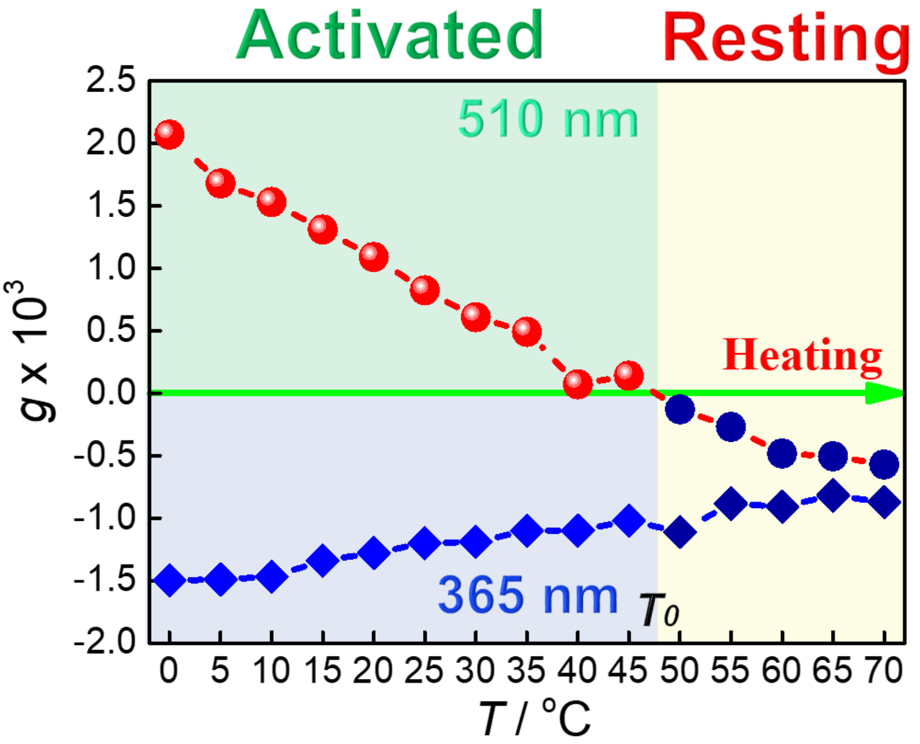

Supplementary Fig. 126 │ V-T anisotropy factor (g) changes of (*in*-R_p/*out*-S_p)-**MUJ1** in the PSS at 365 nm (diamond) and 510 nm (circle) detected at 308 nm in acetonitrile. (T_0 = 47.6 °C)

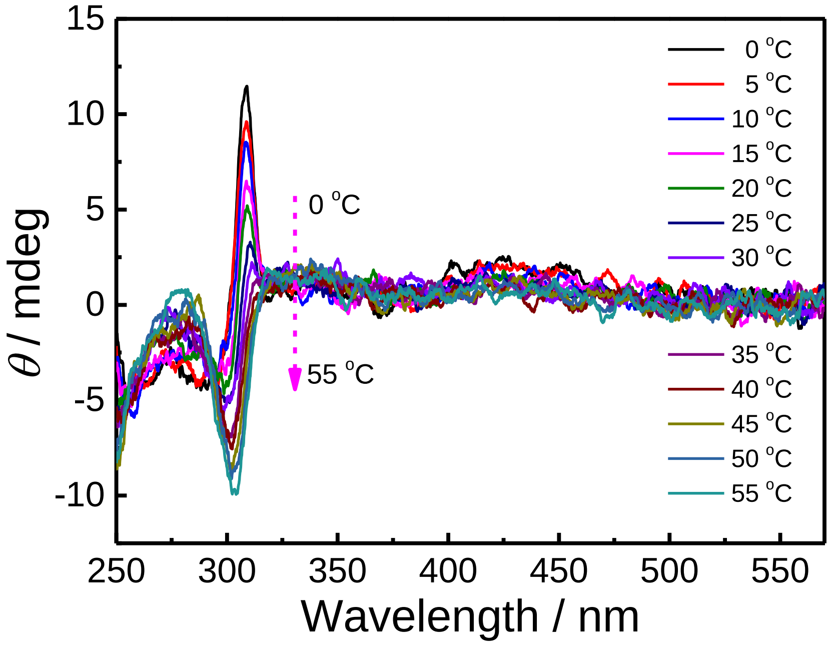

Supplementary Fig. 127 │ V-T CD spectra of (*in*-R_p/*out*-S_p)-**MUJ1** in the PSS at 510 nm in chloroform.

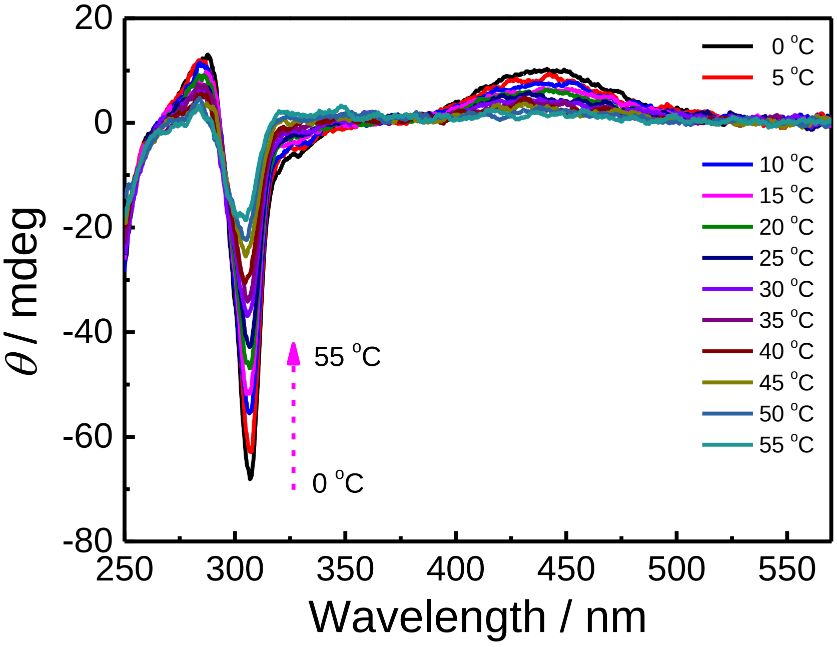

Supplementary Fig. 128 │ V-T CD spectra of (*in*-R_p/*out*-S_p)-**MUJ1** in the PSS at 365 nm in chloroform.

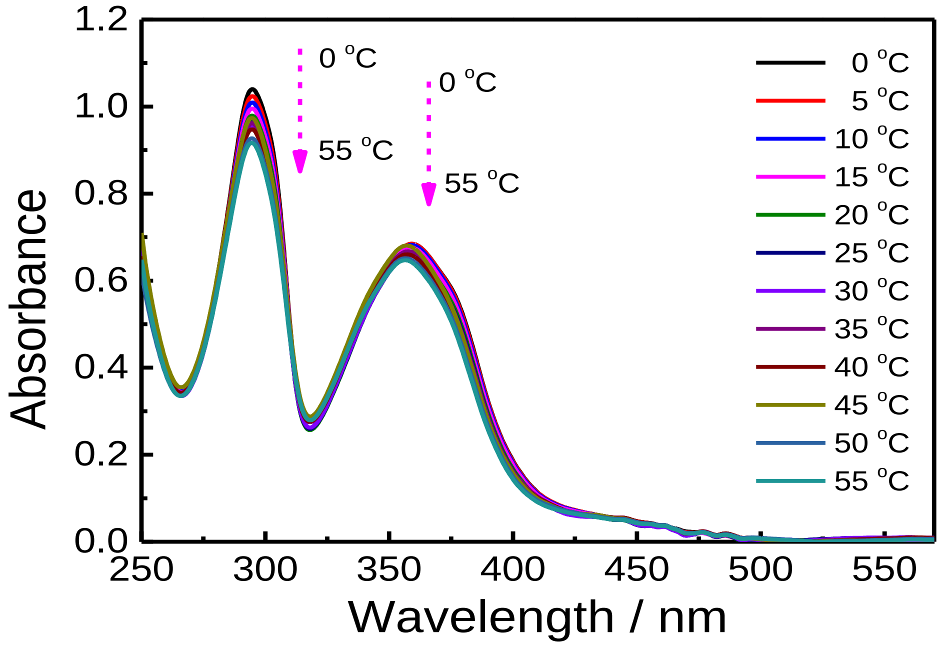

Supplementary Fig. 129 | V-T absorption spectra of (*in*-R_p/*out*-S_p)-**MUJ1** in the PSS at 510 nm in chloroform.

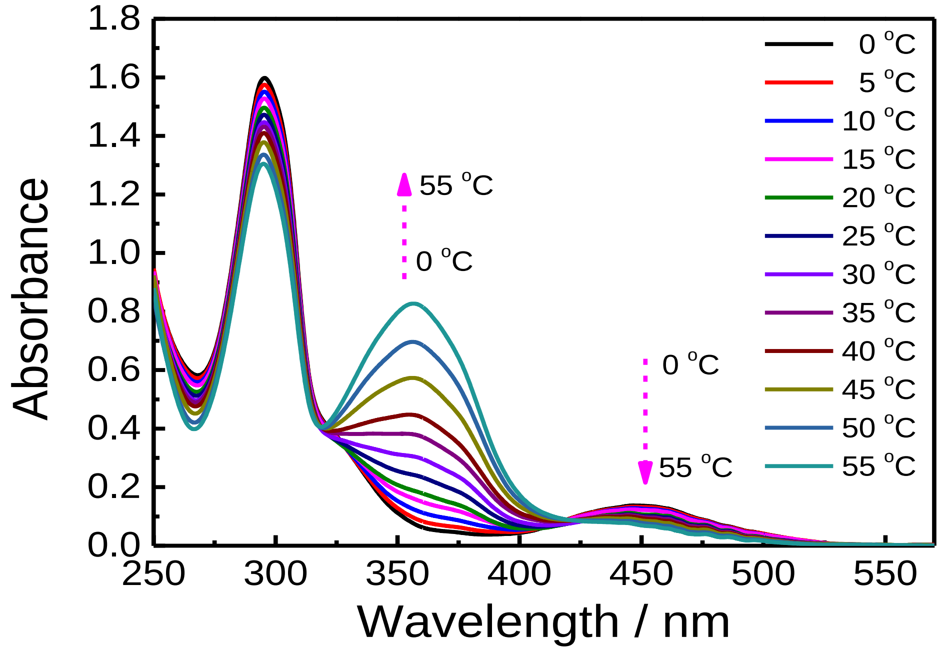

Supplementary Fig. 130 | V-T absorption spectra of (*in*-R_p/*out*-S_p)-**MUJ1** in the PSS at 365 nm in chloroform.

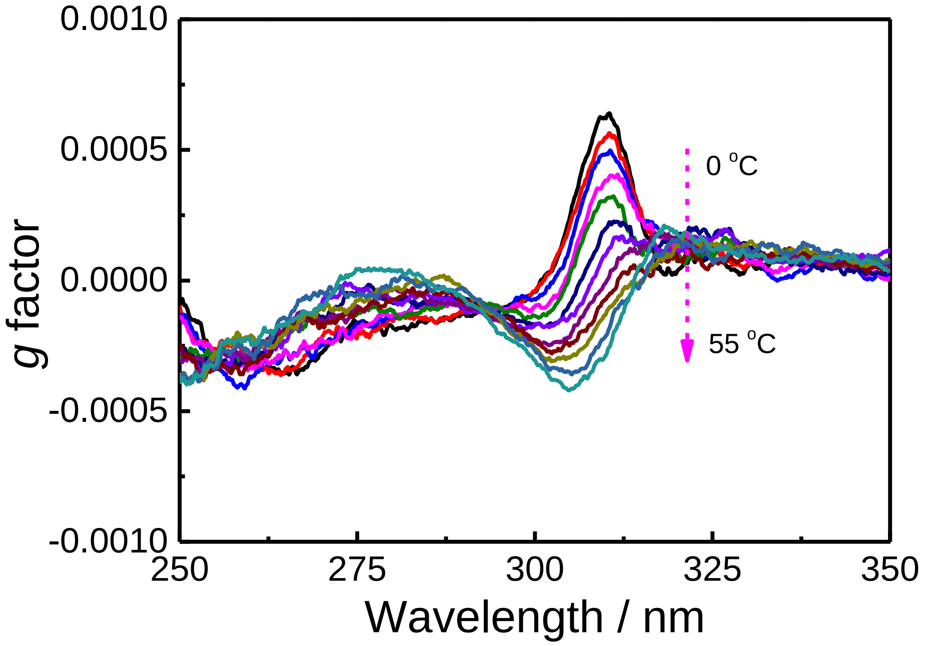

Supplementary Fig. 131 | V-T *g* factor changes of (*in*-R_p/*out*-S_p)-**MUJ1** in the PSS at 510 nm in chloroform.

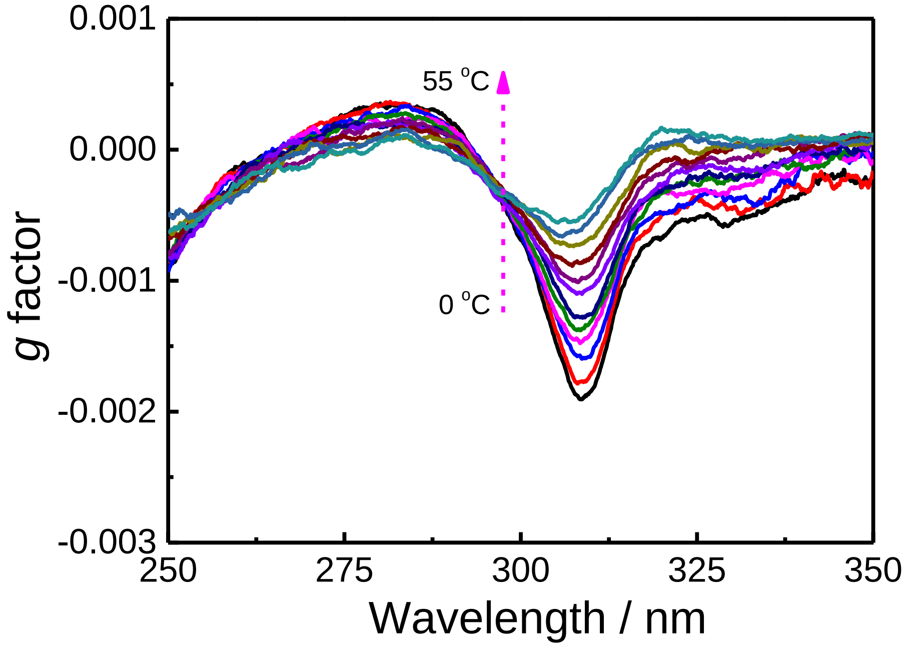

Supplementary Fig. 132 | V-T *g* factor changes of (*in*-R_p/*out*-S_p)-**MUJ1** in the PSS at 365 nm in chloroform.

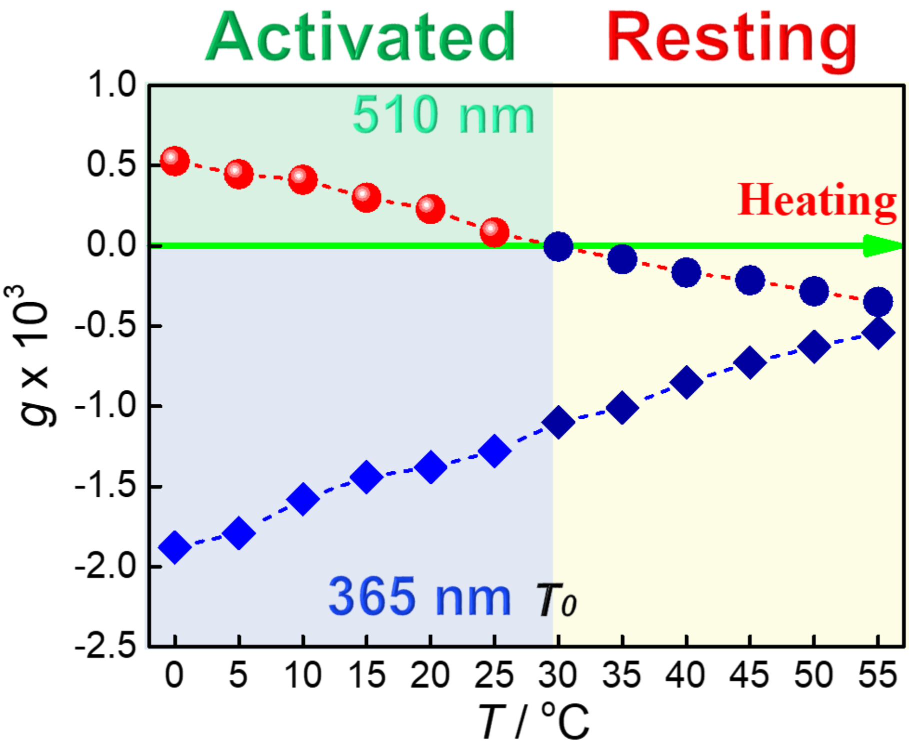

Supplementary Fig. 133 │ V-T anisotropy factor (g) changes of (*in*-R_p/*out*-S_p)-**MUJ1** in the PSS at 365 nm (diamond) and 510 nm (circle) detected at 308 nm in chloroform. (T_0 = 29.7 °C)

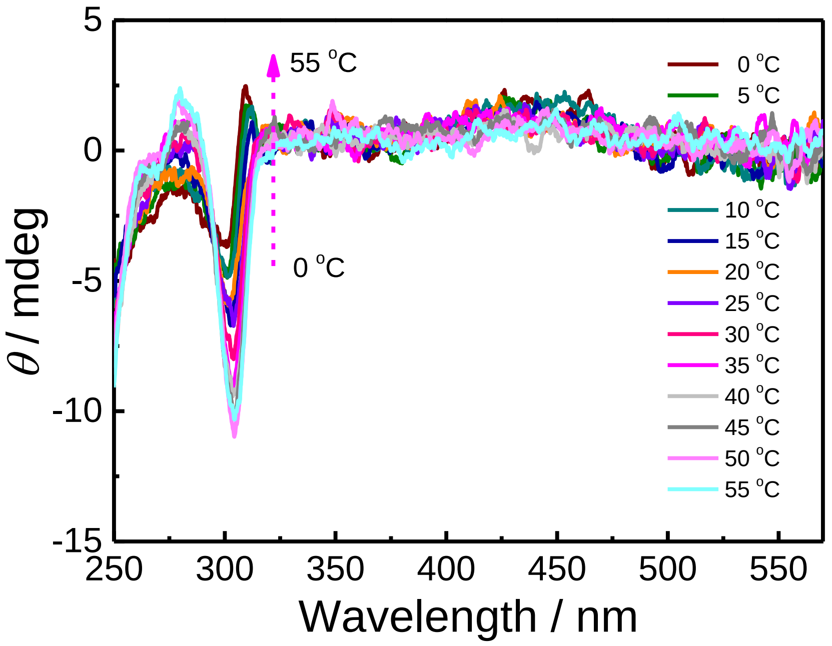

Supplementary Fig. 134 │ V-T CD spectra of (*in*-R_p/*out*-S_p)-**MUJ1** in the PSS at 510 nm in a mixed solvent (tetrahydrofuran/acetonitrile = 1 : 1).

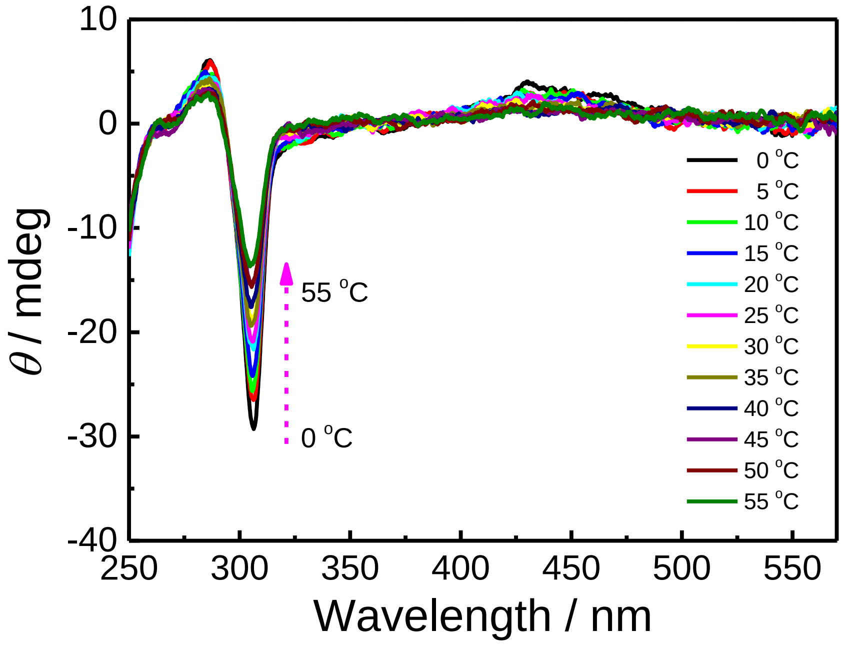

Supplementary Fig. 135 │ V-T CD spectra of (*in*-R_p/*out*-S_p)-**MUJ1** in the PSS at 365 nm in a mixed solvent (tetrahydrofuran/ acetonitrile = 1 : 1).

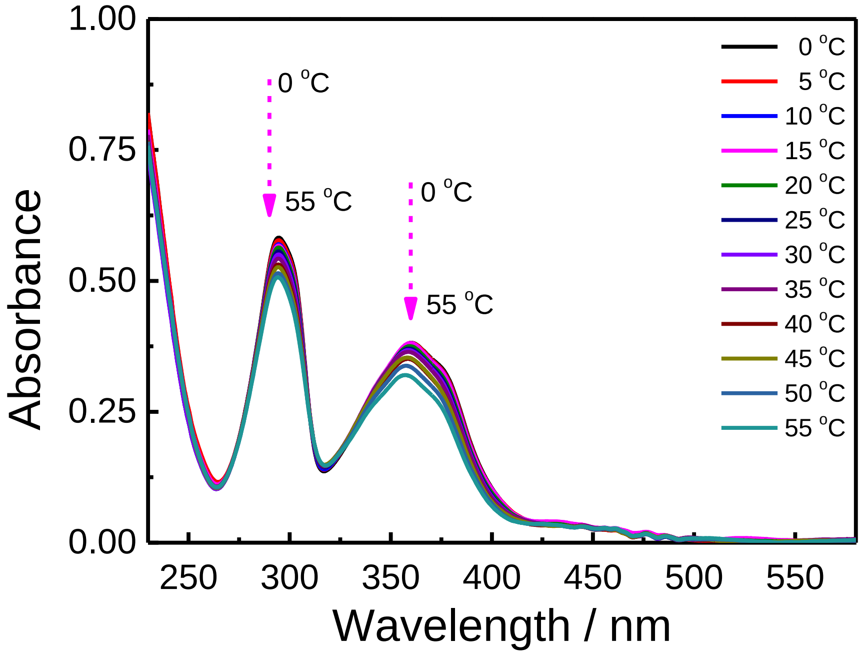

Supplementary Fig. 136 │ V-T absorption spectra of (*in*-R_p/*out*-S_p)-**MUJ1** in the PSS at 510 nm in a mixed solvent (tetrahydrofuran/ acetonitrile = 1 : 1).

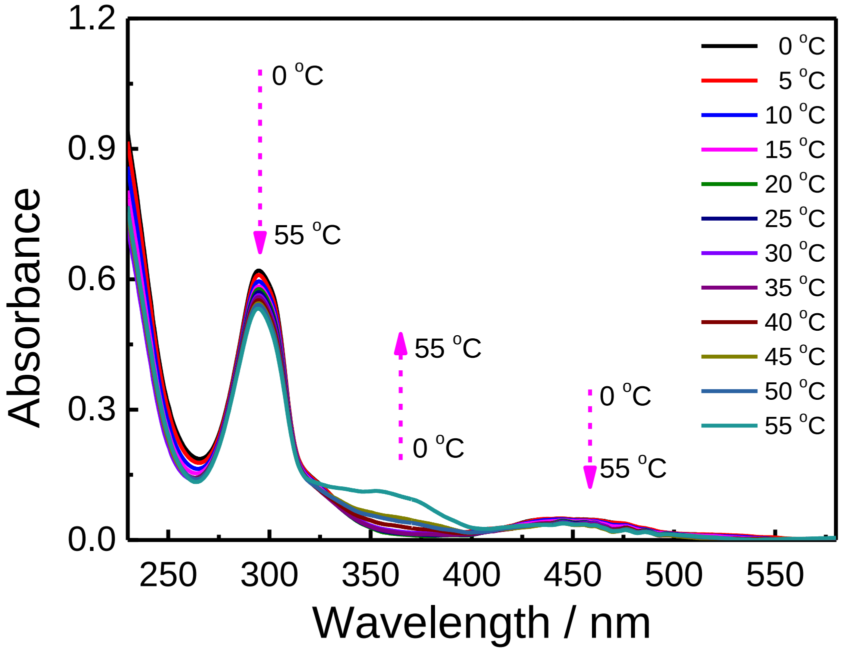

Supplementary Fig. 137 | V-T absorption spectra of (*in*-R_p/*out*-S_p)-**MUJ1** in the PSS at 365 nm in a mixed solvent (tetrahydrofuran/ acetonitrile = 1 : 1).

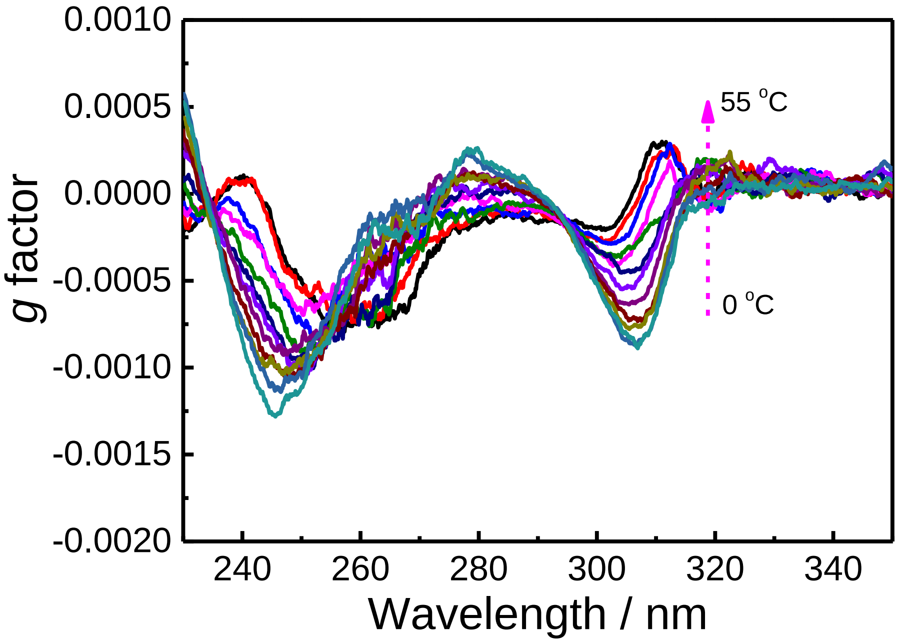

Supplementary Fig. 138 | V-T *g* factor changes of (*in*-R_p/*out*-S_p)-**MUJ1** in the PSS at 510 nm in a mixed solvent (tetrahydrofuran/ acetonitrile = 1 : 1).

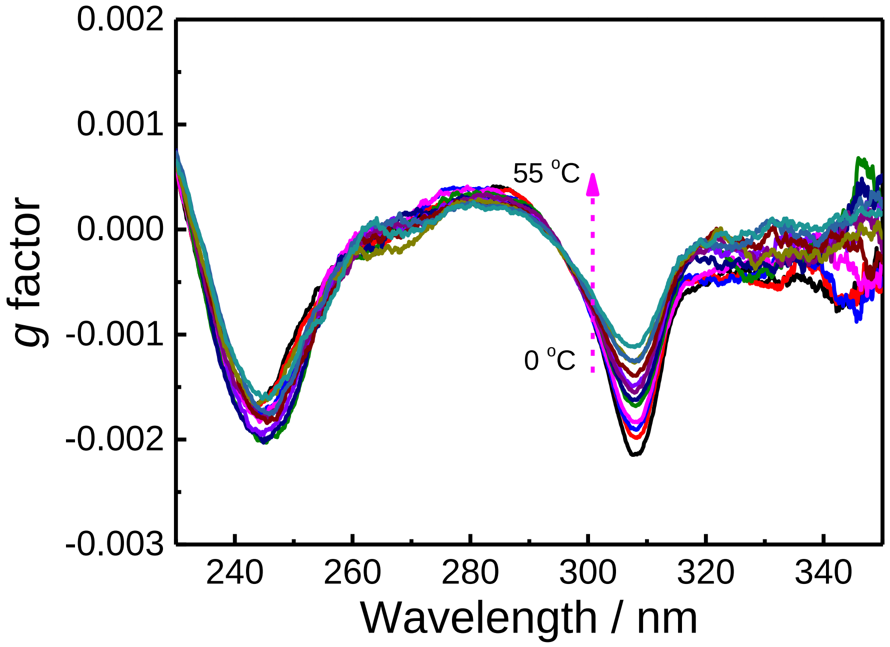

Supplementary Fig. 139 │ V-T *g* factor changes of (*in*-R_p/*out*-S_p)-**MUJ1** in the PSS at 365 nm in a mixed solvent (tetrahydrofuran/ acetonitrile = 1 : 1).

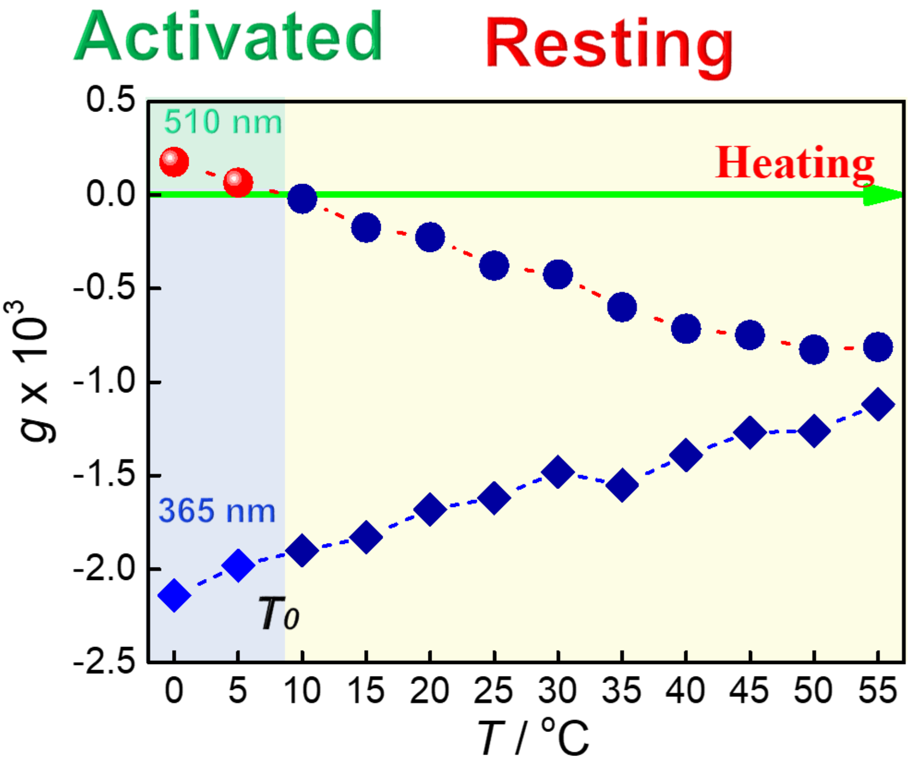

Supplementary Fig. 140 │ V-T anisotropy factor (*g*) changes of (*in*-R_p/*out*-S_p)-**MUJ1** in the PSS at 365 nm (diamond) and 510 nm (circle) detected at 308 nm in a mixed solvent (tetrahydrofuran/ acetonitrile = 1 : 1). (T_0 = 8.8 °C)

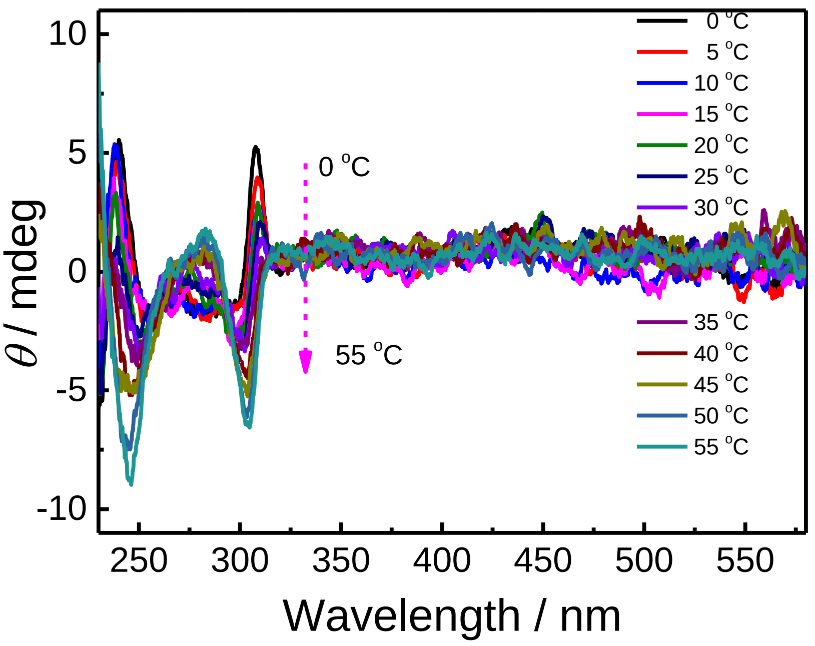

Supplementary Fig. 141 | V-T CD spectra of (*in*-R_p/*out*-S_p)-**MUJ1** in the PSS at 510 nm in a mixed solvent (tetrahydrofuran/acetonitrile = 19 : 1).

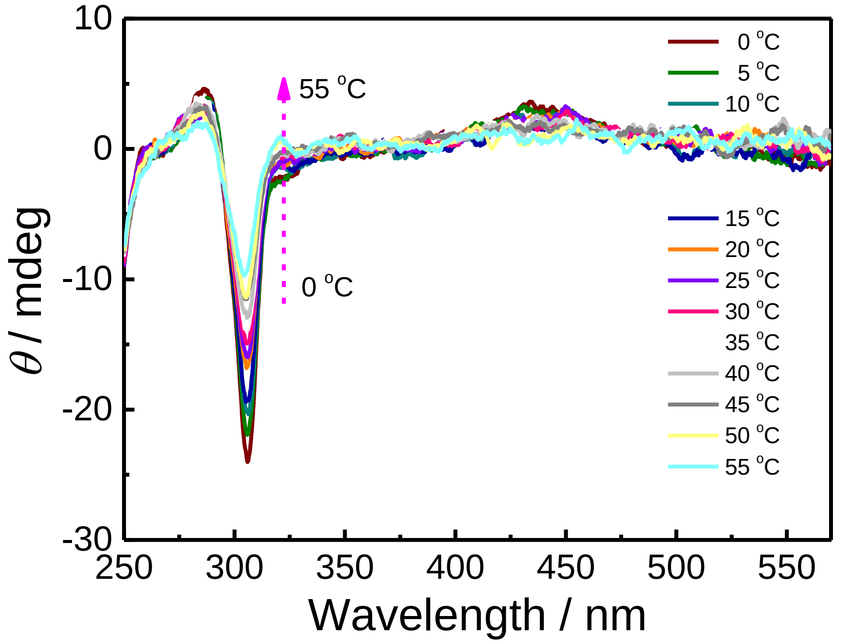

Supplementary Fig. 142 | V-T CD spectra of (*in*-R_p/*out*-S_p)-**MUJ1** in the PSS at 365 nm in a mixed solvent (tetrahydrofuran/acetonitrile = 19 : 1).

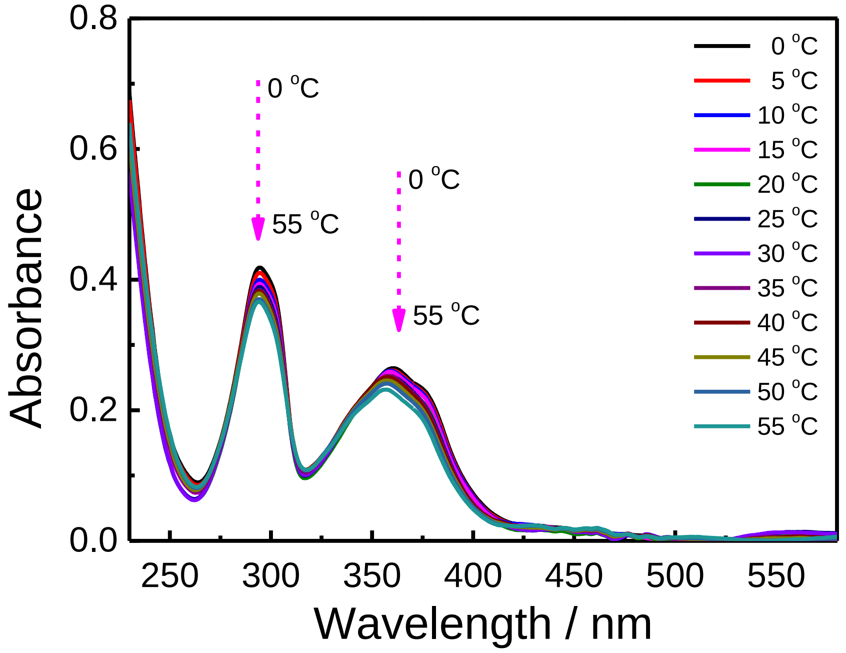

Supplementary Fig. 143 │ V-T absorption spectra of (*in-R*$_p$/*out-S*$_p$)-**MUJ1** in the PSS at 510 nm in a mixed solvent (tetrahydrofuran/ acetonitrile = 19 : 1).

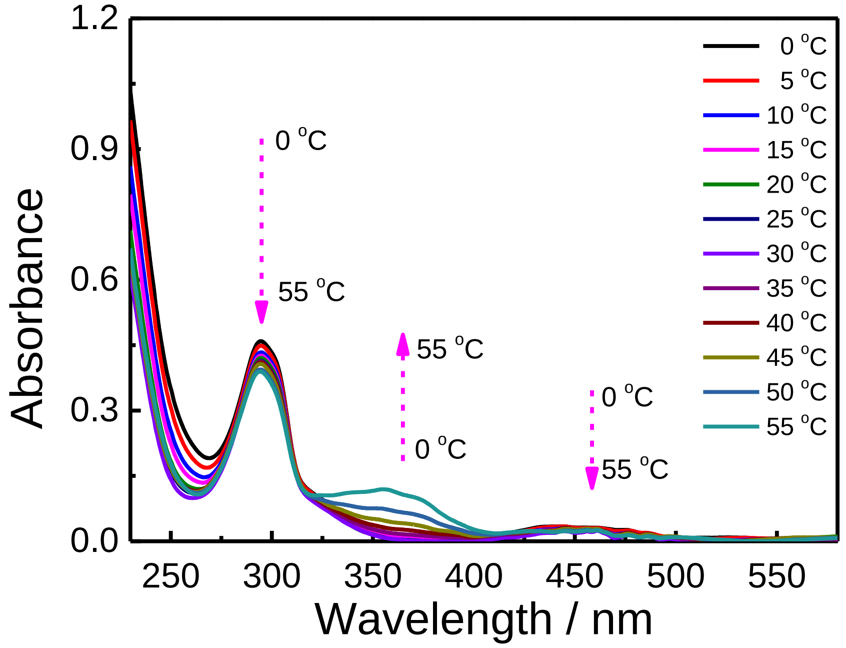

Supplementary Fig. 144 │ V-T absorption spectra of (*in-R*$_p$/*out-S*$_p$)-**MUJ1** in the PSS at 365 nm in a mixed solvent (tetrahydrofuran/ acetonitrile = 19 : 1).

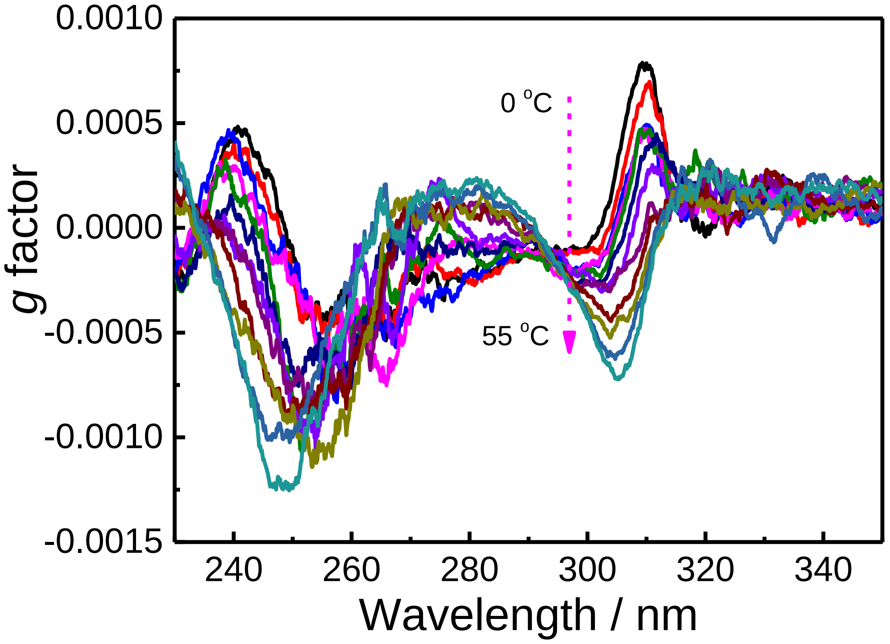

Supplementary Fig. 145 | V-T *g* factor changes of (*in*-R_p/*out*-S_p)-**MUJ1** in the PSS at 510 nm in a mixed solvent (tetrahydrofuran/ acetonitrile = 19 : 1).

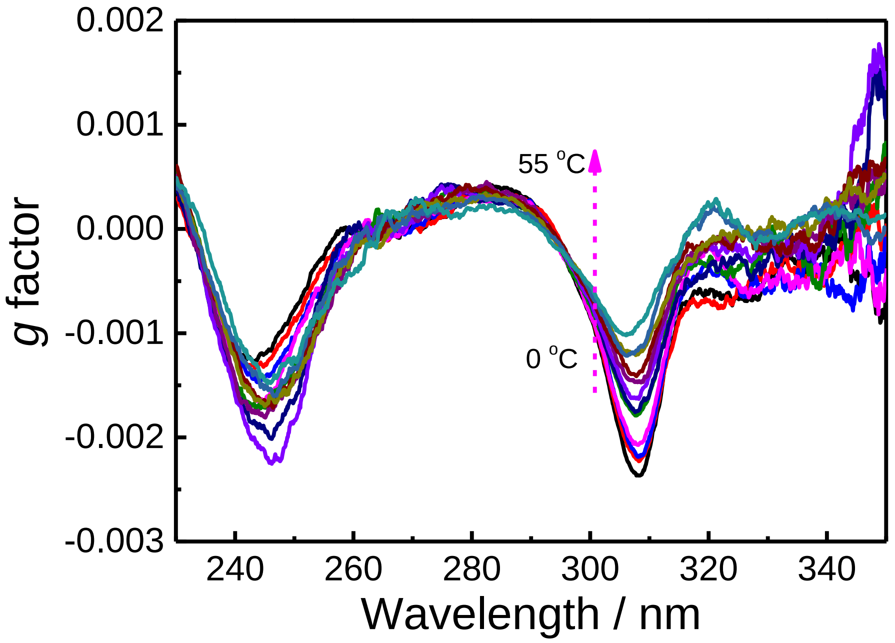

Supplementary Fig. 146 | V-T *g* factor changes of (*in*-R_p/*out*-S_p)-**MUJ1** in the PSS at 365 nm in a mixed solvent (tetrahydrofuran/ acetonitrile = 19 : 1).

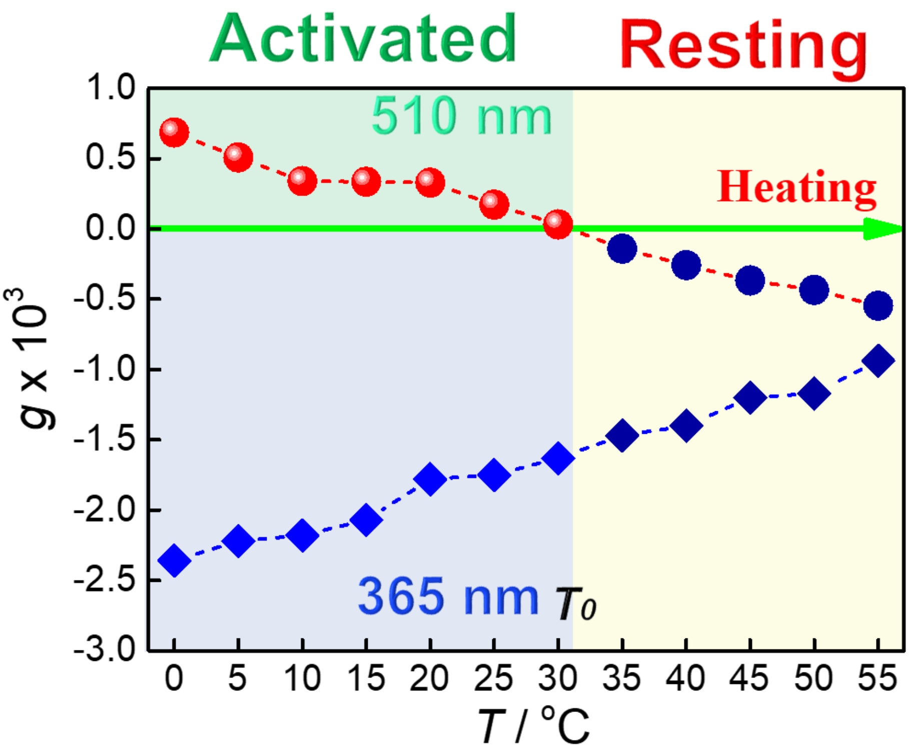

Supplementary Fig. 147 │ V-T anisotropy factor (*g*) changes of (*in*-R_p/*out*-S_p)-**MUJ1** in the PSS at 365 nm (diamond) and 510 nm (circle) detected at 308 nm in a mixed solvent (tetrahydrofuran/ acetonitrile = 19 : 1). (T_0 = 31.0 °C)

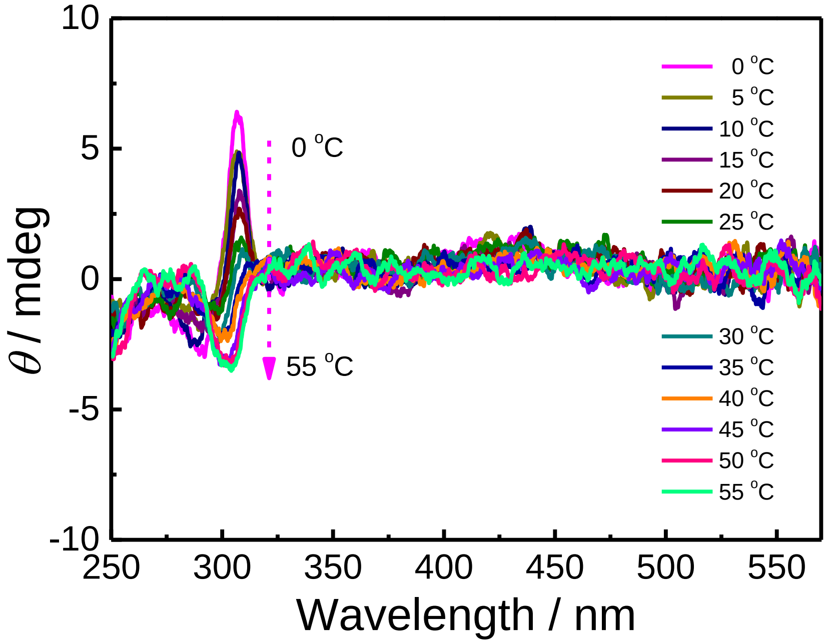

Supplementary Fig. 148 │ V-T CD spectra of (*in*-R_p/*out*-S_p)-**MUJ1** in the PSS at 510 nm in a mixed solvent (tetrahydrofuran/ acetonitrile = 1 : 19).

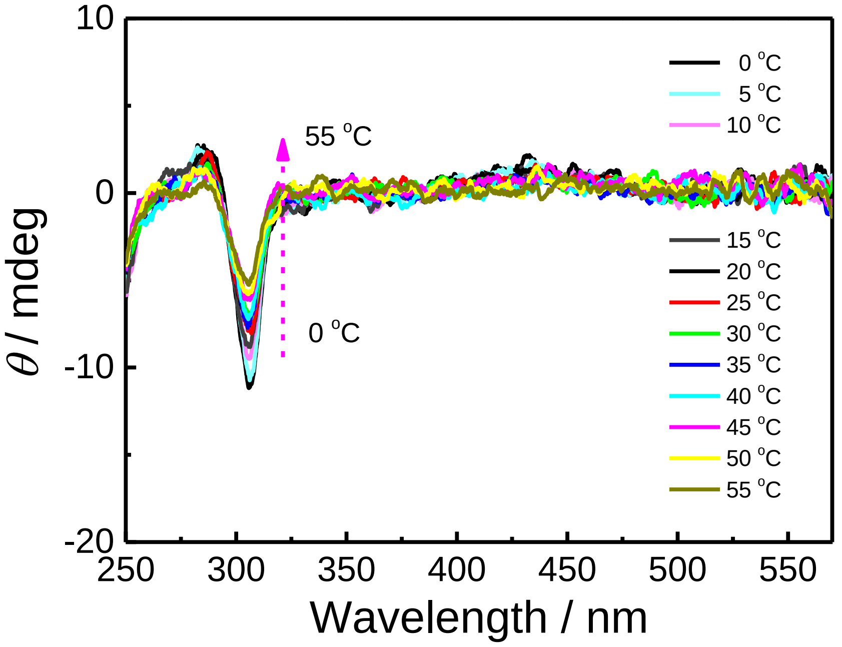

Supplementary Fig. 149 │ V-T CD spectra of (*in*-R_p/*out*-S_p)-**MUJ1** in the PSS at 365 nm in a mixed solvent (tetrahydrofuran/ acetonitrile = 1 : 19).

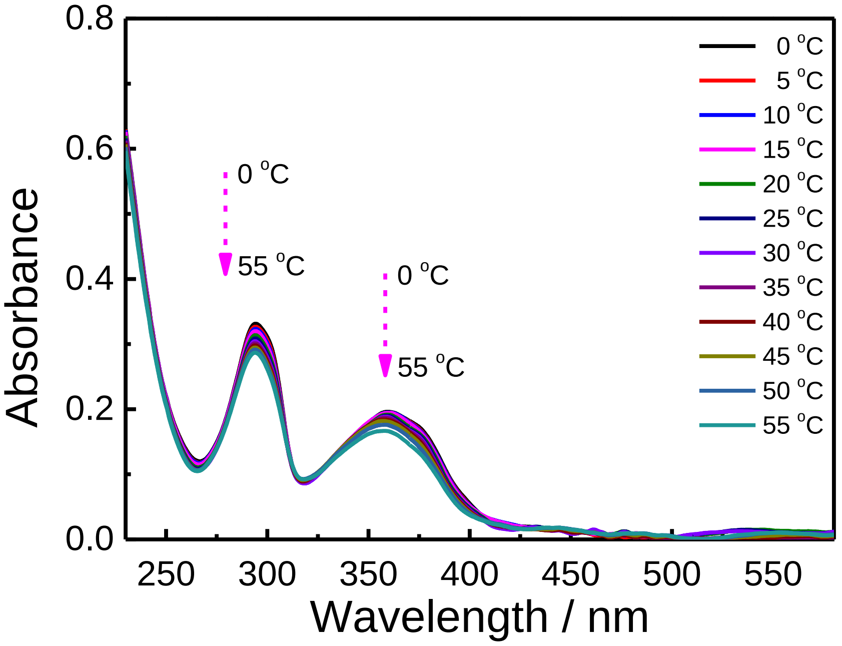

Supplementary Fig. 150 │ V-T absorption spectra of (*in*-R_p/*out*-S_p)-**MUJ1** in the PSS at 510 nm in a mixed solvent (tetrahydrofuran/ acetonitrile = 1 : 19).

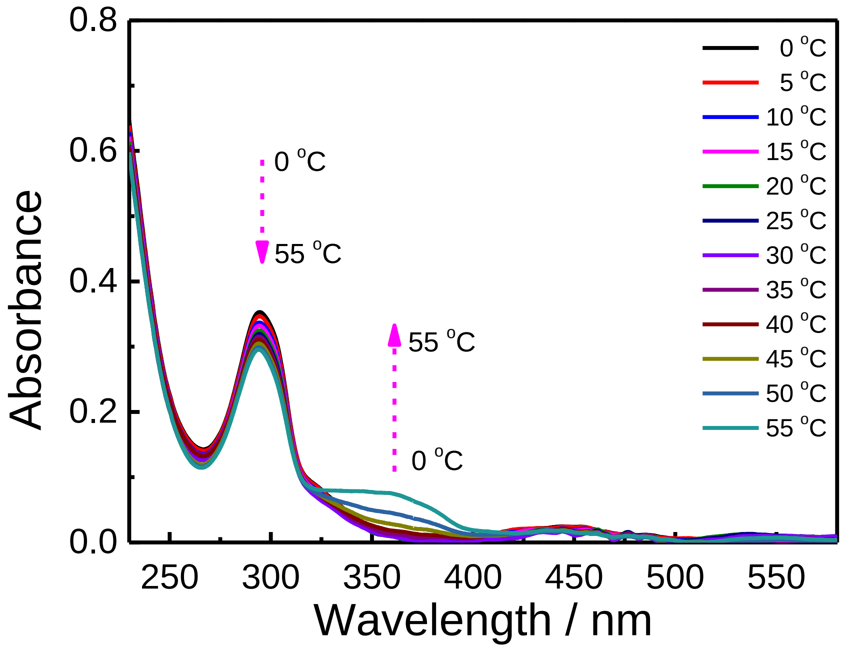

Supplementary Fig. 151 │ V-T absorption spectra of (*in-R_p/out-S_p*)-**MUJ1** in the PSS at 365 nm in a mixed solvent (tetrahydrofuran/ acetonitrile = 1 : 19).

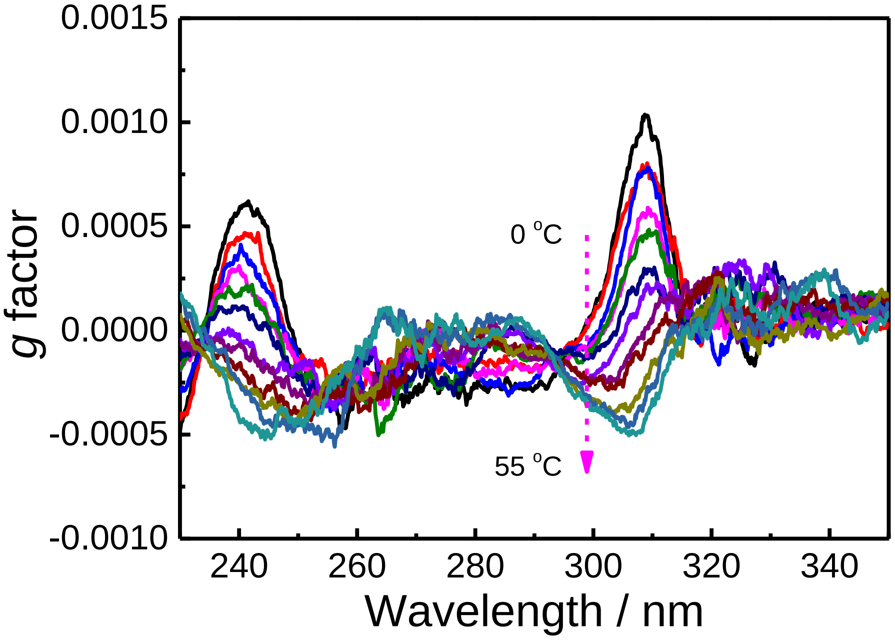

Supplementary Fig. 152 │ V-T *g* factor changes of (*in-R_p/out-S_p*)-**MUJ1** in the PSS at 510 nm in a mixed solvent (tetrahydrofuran/ acetonitrile = 1 : 19).

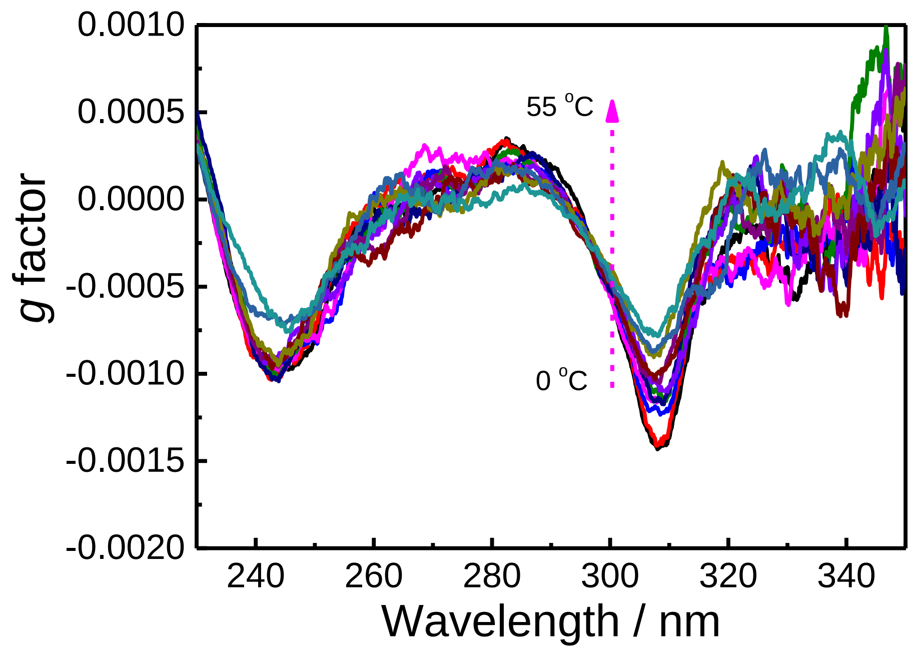

Supplementary Fig. 153 | V-T *g* factor changes of (*in-R*$_p$/*out-S*$_p$)-**MUJ1** in the PSS at 365 nm in a mixed solvent (tetrahydrofuran/ acetonitrile = 1 : 19).

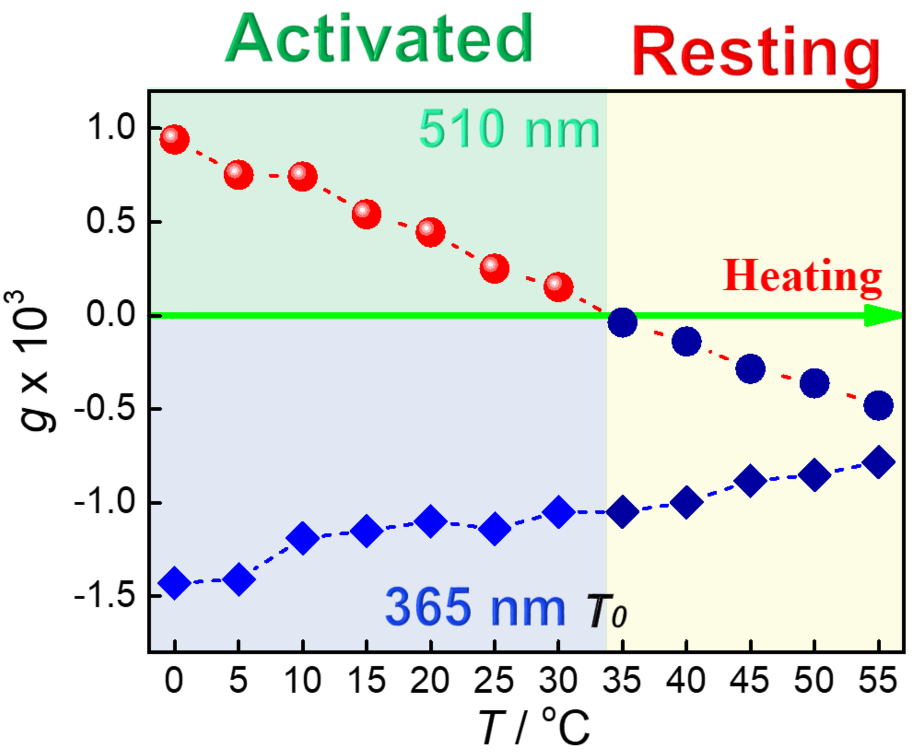

Supplementary Fig. 154 | V-T anisotropy factor (*g*) changes of (*in-R*$_p$/*out-S*$_p$)-**MUJ1** in the PSS at 365 nm (diamond) and 510 nm (circle) detected at 308 nm in a mixed solvent (tetrahydrofuran/ acetonitrile = 1 : 19). (T_0 = 34.0 °C)

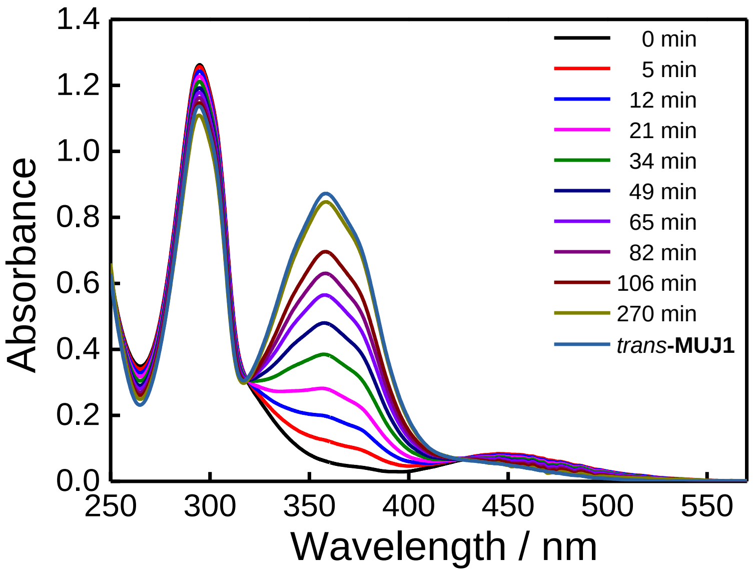

Supplementary Fig. 155 │ CD spectra of (*in*-R_p/*out*-S_p)-**MUJ1** in the PSS at 365 nm (0.042 mM, tetrahydrofuran) after being kept at 50 °C for different times.

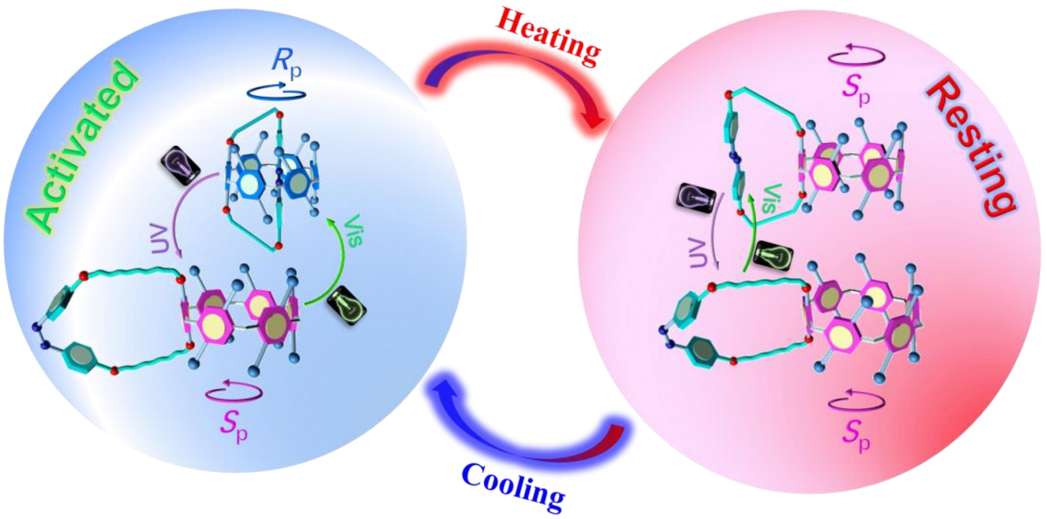

Supplementary Fig. 156 │ Suppositional mechanism of light-driven chirality switching of (*in*-R_p/*out*-S_p)-**MUJ1**.

17. Calculated CD Spectra of MUJ1

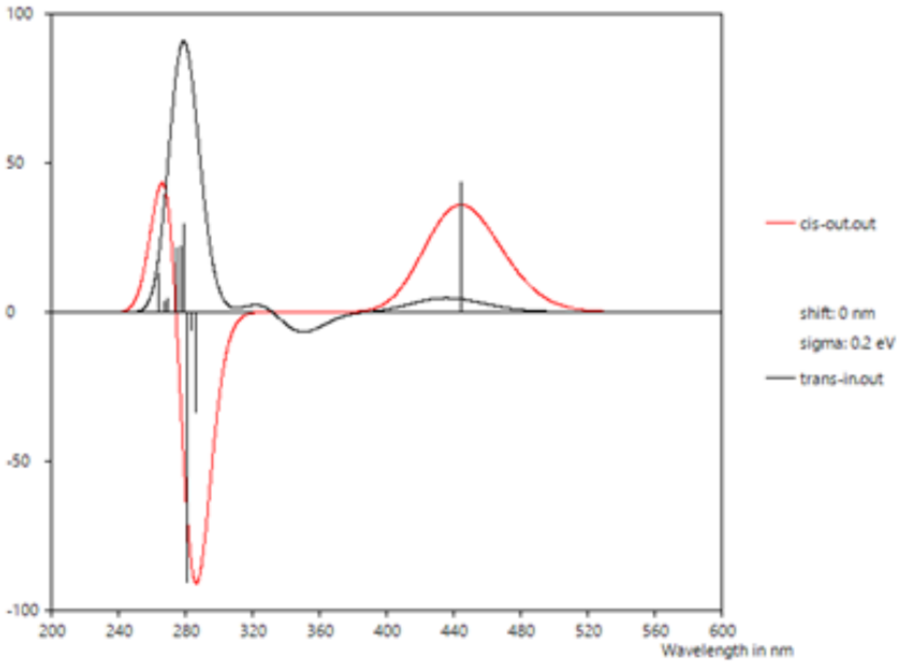

Supplementary Fig. 157 | Calculated CD spectra of *trans*-*in*-(R_p)-**MUJ1** (black line) and *cis*-*out*-(S_p)-**MUJ1** (red line).

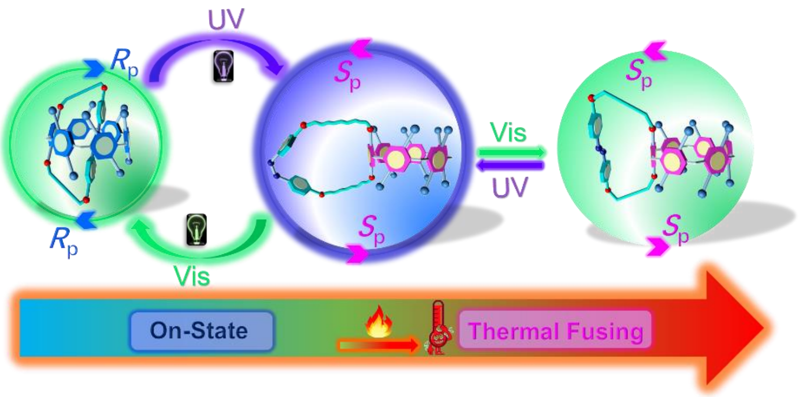

Supplementary Fig. 158 | Table of contents of overtemperature-protection intelligent molecular photoswitches. Switching of the chiroptical properties of an intelligent molecular photoswitch by wavelength-selective photoirradiation that is regulated by the temperature to achieve overtemperature protection.

References

1. Ogoshi, T. *et al.* Synthesis and conformational characteristics of alkyl-substituted pillar[5]arenes. *J. Org. Chem.* **75**, 3268-3273 (2010).
2. Tao, H. *et al.* Synthesis and host-guest properties of pillar[6]arenes. *Sci China Chem* **55**, 223-228 (2011).
